# Effects of hospital facilities on patient outcomes after cancer surgery: an international, prospective, observational study

**DOI:** 10.1016/S2214-109X(22)00168-1

**Published:** 2022-05-24

**Authors:** GlobalSurg Collaborative and NIHR Global Health Research Unit on Global Surgery, Stephen R Knight, Catherine A Shaw, Riinu Pius, Thomas M Drake, Lisa Norman, Adesoji O Ademuyiwa, Adewale O Adisa, Maria Lorena Aguilera, Sara W Al-Saqqa, Ibrahim Al-Slaibi, Aneel Bhangu, Bruce M Biccard, Peter Brocklehurst, Ainhoa Costas-Chavarri, Kathryn Chu, Anna Dare, Muhammed Elhadi, Cameron J Fairfield, J Edward Fitzgerald, Dhruv Ghosh, James Glasbey, Mark I. van Berge Henegouwen, J.C. Allen Ingabire, T Peter Kingham, Marie Carmela Lapitan, Ismaïl Lawani, Bettina Lieske, Richard Lilford, Janet Martin, Kenneth A McLean, Rachel Moore, Dion Morton, Dmitri Nepogodiev, Faustin Ntirenganya, Francesco Pata, Thomas Pinkney, Ahmad Uzair Qureshi, Antonio Ramos-De la Medina, Aya Riad, Hosni Khairy Salem, Joana Simões, Richard Spence, Neil Smart, Stephen Tabiri, Hannah Thomas, Thomas G Weiser, Malcolm West, John Whitaker, Ewen M Harrison, Arben Gjata, Maria Marta Modolo, Sebastian King, Erick Chan, Sayeda Nazmun Nahar, Ade Waterman, Dominique Vervoort, Ismaïl Lawani, Alemayehu Ginbo Bedada, Bernardo De Azevedo, Ana Gabriela Figueiredo, Manol Sokolov, Venerand Barendegere, Gerald Ekwen, Arnav Agarwal, Anna Dare, Qinyang Liu, Juan Camilo Correa, Kalisya Luc Malemo, Jacques Bake, Jakov Mihanovic, Kamila Kunčarová, Julius Orhalmi, Hosni Salem, Jyri Teras, Aristotelis Kechagias, Alexis P Arnaud, Judith Lindert, Stephen Tabiri, Vasileios Kalles, Maria-Lorena Aguilera-Arevalo, Gustavo Recinos, Zsolt Baranyai, Basant Kumar, Harish Neelamraju Lakshmi, Sanoop Koshy Zachariah, Philip Alexander, Sunil Kumar Venkatappa, C Pramesh, Radhian Amandito, Christina Fleming, Luca Ansaloni, Francesco Pata, Gianluca Pellino, Ahmed M. Altibi, Ibrahim Nour, Intisar Hamdun, Muhammed Elhadi, Ali M. Ghellai, Donatas Venskutonis, Tomas Poskus, Justas Zilinskas, John Whitaker, Precious Malemia, Yong Yong Tew, Elaine Borg, Sarah Ellul, Antonio Ramos-De la Medina, fatima Zahraa Wafqui, David W Borowski, Anne Sophie van Dalen, Cameron Wells, Harissou Adamou, Adesoji Ademuyiwa, Adewale Adisa, Kjetil Søreide, Ahmad Uzair Qureshi, Ibrahim Al-Slaibi, Sara Al Saqqa, Osaid Alser, Haya Tahboub, Helmut Alfredo Segovia Lohse, Sebastian Shu Yip, Marie Carmela Lapitan, Piotr Major, Joana Simões, António Sampaio Soares, Matei Razvan Bratu, Andrey Litvin, Armen Vardanyan, JC Allen Ingabire, Ainhoa Costas-Chavarri, Ahmad Gudal, Naif Albati, Jovan Juloski, Bettina Lieske, Miran Rems, Sarah Rayne, Stephanie Van Straten, Yoshan Moodley, Kathryn Chu, Rachel Moore, Irene Ortega Vázquez, Jaime Ruiz-Tovar, Kithsiri Janakantha Senanayake, Sujeewa Priyantha Bandara Thalgaspitiya, Omer Abdelbagi Omer, Anmar Homeida, Yucel Cengiz, Daniel Clerc, Muhammad Alshaar, Hanen Bouaziz, Yuksel Altinel, Matthew Doe, Maryna Freigofer, Ella Teasdale, Rakan Kabariti, Joshua Michael Clements, Stephen Richard Knight, Ahsan Ashfaq, Ijeoma Azodo, Gabriela Wagner, Ivan Trostchansky, Mayaba Maimbo, David Linyama, Helidon Nina, Amanda Zeko, Claudio Gabriel Fermani, Maria Marta Modolo, Santiago Villalobos, Federico Carballo, Pablo Farina, Sebastian Guckenheimer, Marilla Dickfos, Ankit Ajmera, Chester Chong, Ralph Gourlay, Sikandar Hussaini, Yi Jia Lee, Adeeb Majid, Peter Martin, Rebecca Miles, Owen James Morris, Jamie Phua, William Ridley, Tarunpreet Saluja, Ryan Renxin Tan, Jen Teh, Anna Wells, Bharti Arora, Qaasim Dollie, Debbie Ho, Yanru Ma, Omattage Mahasha Perera, Anthony Truong, Amanda Caroline Dawson, Bryan Lim, Upuli Pahalawatta, Jacqueline Phan, Xiao-Ming Sarah Woon-Shoo-Tong, Andrea Yeoh, Lillian Charman, Andrew Drane, Sharon Laura, Charmaine Chu Wen Lo, Amy Mozes, Rita Poon, Hao Han Tan, Ellen Wall, Prakshi Chopra, Jasmine De Giovanni, Bal Dhital, Brian Draganic, Alexander Duller, Jonathan Gani, Yao Kuan Goh, Jun Young Jeong, Brendan McManus, Prakash Nagappan, Peter Pockney, Anya Rugendyke, Mahsa Sarrami, Stephen Smith, Vanessa Wills, Hsu Ven Wong, Geoffrey Ye, Geoffrey Zhang, Ethan Brooker, Daniel Feng, Bonnie Lau, Carlin Ngai, Sarah Birks, David Gyorki, Jaime Otero de Pablos, Ali Abbosh, Chris Gillespie, Ahmed Mahmoud, Bianca Kwan, Joshua Lawson, Andrea Warwick, Janne Bingham, Andrew J Cockbain, Nagendra Naidu Dudi-Venkata, Jordan Ellaby-Hall, Ben Finlay, Emily Humphries, Jade Pisaniello, Monique Pisaniello, Salma Salih, Tarik Sammour, Haidar Hadri Abd Wahab, April De Silva, Nicola Hayward, Kartik Iyer, Guy Maddern, Gian Andrea Prevost, Naga Annapureddy, Krishna Pranathi Settipalli, Jeremy Yeo, Lucy Hempenstall, Lily Pham, Shaun Purcell, Cherry Talavera, Ashish I Vaska, Gurpreet Chaggar, Phillip Chrapko, Annelise Cocco, Sarah Michelle Crystal Jade Coulter-Nile, Grahame Ctercteko, James French, Houchen Gong, Martijn Gosselink, Thuvarahan Jegathees, Ivan Jin, Michelle Kalachov, Kathryn Kiefhaber, Katherine Lee, Jason Luong, Steven Phan, Henry Pleass, Kelly Veale, Zhi Zeng, Angela Au, Ashe DeBiasio, Idy Deng, Jananee Myooran, Amrita Nair, Peter Stewart, Anton Stift, Lukas Walter Unger, Kerstin Wimmer, Nabila Ahmed, Syed Hasan, Saber Rahman, Margaret O'Shea, Greg Padmore, Adrian Peters, Pietro Perduca, Guenda Pulcina, Nicolas Tinton, Frederic Buxant, Elsa Dabin, Giulia Garofalo, Francis Dossou, Ismaïl Lawani, Freddy Houehanou Rodrigue Gnangnon, Yacoubou Imorou Souaibou, Alemayehu Ginbo Bedada, Pako Motlaleselelo, Omphile Tlhomelang, Igor Lima Buarque, Gustavo Mendonça Ataíde Gomes, Aldo Vieira Barros, Ilia Batashki, Nikolai Damianov, Vladislav Stoyanov, Dragomir Dardanov, Svilen Maslyankov, Plamen Petkov, Manol Sokolov, George Todorov, Evgeni Zhivkov, Aygulya Akisheva, Miguel Angel Castilla Moreno, Geno Genov, Ivelina Ilieva, Tsvetomir Ivanov, Martin Karamanliev, Azhar Khan, Emil Mitkov, Tsanko Yotsov, Boyko Atanasov, Nikolay Belev, Mihail Slavchev, Carlos Nsengiyumva, Elgan Jones, Simon Stock, Gerald Ekwen, Steve Kyota, James Brown, Tresor Mabanza K., Lemery Nigo Samuel, Chidi Otuneme, Ngwang Prosper, Franklin Umenze, Marylise Boutros, Natasha Caminsky, Sinziana Dumitra, Richard Garfinkle, Dominique Morency, Ebram Salama, Alexander Banks, Lorenzo Ferri, Haitian He, Amit Katz, Alexander Sender Liberman, Sarkis Meterissian, Allison Pang, Elena Parvez, Arnav Agarwal, Anna Dare, Usmaan Hameed, Fahima Osman, Sangita Sequeira, Natalie Coburn, Anna Dare, Alisha Jaffer, Paul Karanicolas, Matthew Mosseler, Reilly Musselman, Xinyuan Liu, Ching Wan Yip, Juan Sebastian Garces-Otero, Carolina Guzman, Sebastian Sierra, Andres Uribe Valencia, Paulo Andrés Cabrera Rivera, Saul Camelo, Andrea Gonzalez, Alejandro González-Orozco, Manuel Santiago Mosquera Paz, Carlos J- Perez Rivera, Felipe Gonzalez, Andres Isaza-Restrepo, Laura Nino- Torres, Natalia Arias Madrid, Maria Clara Mendoza Arango, Sebastian Sierra, Jacques Bake, Justin Tsandiraki, Damir Jemendžić, Branislav Kocman, Oliver Šuman, Renata Canic, Darko Jurišić, Ivana Karakas, Ana Krizanovic Rupcic, Vlatka Pitlovic, Josip Samardžić, Mario Kopljar, Ivan Bacic, Edgar Domini, Robert Karlo, Jakov Mihanovic, Danijela Miljanić, Andrea Simic, Mariam Ahmed, Majdi Al Nassrallah, Rabiya Altaf, Talal Amjad, Ruba Eltoum, Heba Haidar, Alhassan Hassan, Omar Khalil, Marwan Qasem, Rommel Ramesh, Gautham Sajith, Maham Wisal, Jan Žatecký, Michele Bujda, Katerina Jirankova, Ales Paclik, Aya Abdallah, Mariam Abdulgawad Almogy, Esraa Ayman El-sawy, Ahmed Moustafa ElFayoumy, Nourhan Elghareeb, Nourhan Ahmed Esmat, Ahmed Fadel, Abdullah Habater, Heba Hamdy, Amr Hefni, Marwa Kamal, Norhan Mohamed Abobakr, Ahmed Sayed, Nancy Shaker, Ehab Taha, Hoda Tharwat, Omar Zakaria, Ibrahem Abdelmotaleb, Ali Al-Dhufri, Hamza S. Al-Himyari, Enas El sheikh, Asmaa Eldmaty, Aya Elkhalawy, Ahmed M.Elkhashen, Kithara Magdy, Safa Mostafa, Habib Doutoum Sadia, Mohamed mahmoud Saleh, Dina Samir, Mohamed Yahia Mohamed Ali, Mahmoud A. Nassar, Samar Abdelhady, Aly Abdelrazek, Israa Abdelsalam, Aya El-Sawy, Eman Essam, Mohamed Gadelkarim, Khaled Ghaly, Mohamed Hassabalnaby, Rana Masarani, Nourhan Mohamed Shaaban, Ahmed Sabry, Menatalla Salem, Nourhan Akram Soliman, Diaaaldin Zahran, Moustafa Ramadan Abou El.soud, Esraa Tarek Badr, Hala Borham, Nehal Elmeslemany, Mohammad Elsayed, Fawzia Elsherif, Sara Eslam, Gehad Gaber, Sondos Ibrahim, Yara Kamh, Abdelrahman Mahmoud, Shimaa gamal Mohamed, Eman Morshedy, Cinderella Omar, Fatima Salem Soliman, Shaza Abdelkawy, Naglaa Abdelmohsen, Mahmoud Abdelshakour, Ahmed Dahy, Norhan Gamal, Mohammed Gamal, Ahmad Hasan, Helal Hetta, Nehad Mousa, Mohamed Omar, Somia Rabie, Mahmoud Saad, Bakeer Saleh, Marwa Sayed Mohamed, Muhammad Shawqi, Heba Abdelhady Mousa, Mostafa Alnoury, Mohamed Elbealawy, Ahmed Elshafey, Muhammad Essam Ibrahim El Desouki Muhammad Ahmed, Mennatullah Ghonaim, Fawzy Hgag, Mohamed Ibrahim, Mahmoud Morsy, Mohamed Reda Loaloa, Ahmed Refaat, Hadeer Samir, Fatma Shahien, Mohamed Sobhy, Fathy Sroor, Esraa Abdellatif, Marina Adel, Amr Abdelghani Afifi, Eman Afifi, Marco Antaky, Amr Dawoud, Naira El Zoghby, Amira El-remaily, Ali Abdelazez Elzanfaly, Ahmed Gadallah, Fatma Alzahraa Gamal, Omar Hashem, Shrouk Medhat Youssef, Aliaa Muhammad Attyah, Malak Munir, Omar Shazly, Esraa Taha, Karim Wilson, Sawsan Adel, Asmaa Ali, Esraa Eid, Esraa Elhelow, Marwa Elmahdy, Bassant Elshatby, Amany Hossam el-din Zakaria, Ahmad Hossny, Eman Ibrahim, Ahmed M.Yonis, Maram Metwalli, Basant Yousry, Esraa Zid, Mina A Yacoub, Ahmed Abdelhakim, Nervana Abouelsoad, Mo'min Alkhatib, Ahmed Ashraf, Alaa Ashraf, Yasmin Elazab, Mahmoud Elfanty, Osama Elkabir, Mai Elsayed, Ahmed Elshimy, Hager Elsobky, John Eskander, Ahmed Gad, Ward Hamsho, Noura Khaled Abdelwahed, Menna Magdy, Dalia Moharam, Abeer Osama, Shereen Ramadan, Radwa Roum, Taqwa Sayed, Tarneem Shehada, Ahmed Mohy Zidan, Khalid Abbas, Amr Ali, Mohamed Attia, Mohamed Balata, Ayman El Nakeeb, Mohamed Ibrahim Elsayed Elewaily, Ahmed Elfallal, Hossam Elfeki, Ahmed Elkhadragy, Sameh Emile, Helmy Ezzat, Hasnaa Hosni, Islam Mansour, Waleed Omar, Gehad Othman, Kareem Sadek, Mostafa Shalaby, Noura Shehab-Eldeen, Rawda Anas khalifa, Helmy Badr, Mostafa Eldeep, Ahmed Eldeep, Amany Eldoseuky mohammed, Salwa Khallaf, Eman Magdy Hegazy, Rokia Mahmoud, Pola Mikhail, Mahmoud Morsi, Sara Mowafy, Dina Raafat, Amina Safy, Marwa Sera, Ahmed shible Sera, Mostafa Salim Mohamed AbdAllah, Muhammad Abdelkader, Abdulrahman Osama Abdou, Ahmedgaber Ahmed, Shireen Gaafar, Fatma Ibrahim negm, Mina Lapic, Ahmed Maher, Hagar Mahmoud, Ahmed Mostafa, Mohamed Samir, Fatma Samy, Nourhan Semeda, Hind I. Shalaby, Alaa El-taweel, Ahmed Galal Elnagar, Ahmed Gamal Hemidan, Mohamed Hussein, Ahmed.A. Kandil, Mf Moawad, Ayat Allah Nasser Hamamah, Mostafa Soliman, Mohamed Abdelkhalek, Noura Abdelmaksoud Tawakel, Ahmed Mohamed Abdelwahed, Alrawy Abdou, Khalid Atallah, Mohammed Yasser Elsherbeny, Eman Emara, Mohamed Hamdy, Omar Hamdy, Amira Haron, Salma Ismail, Islam Hany Metwally, Nihal Mohamed Hamed Elgaml, Ahmed Nassar, Basel Refky, Mirna Sadek, Mahmoud Saleh, Asmaa Yunes, Mai Zakaria, Mohammed Zuhdy, Notila Fayed, Mohammed Mustafa Hassan Mohammed, Sander Kütner, Priit Melnik, Indrek Seire, Jyri Teras, Toomas Ümarik, Eppu Ainoa, Verner Eerola, Hanna Koppatz, Laura Koskenvuo, Ville Sallinen, Sini Takala, Jevgeni Katunin, Aristotelis Kechagias, Arto Turunen, Niki Christou, Muriel Mathonnet, Vincent Lavoue, Krystel Nyangoh Timoh, Lucie Soulabaille, Romain Lesourd, Aude Merdrignac, Laurent Sulpice, Benoît André, Elodie Chantalat, Charlotte Vaysse, Bertrand Dousset, Sebastien Gaujoux, Gregory Martin, Octavian Clonda, Domantas Juodis, Klaus Kienle, Andras Mravik, Samuel Palmer, Gabor Szabadhegyi, Anita Eseenam Agbeko, Solomon Gyabaah, Frank Enoch Gyamfi, Nuhu Naabo, Atta Owusu senior, Joseph Yorke, Frank Owusu, Francis Abantanga, Theophilus Teddy Kojo Anyomih, Abdul-Jalilu Mohammed Muntaka, Emmanuel Owusu Abem, Mohammed Sheriff, Stephen Tabiri, Paul M. Wondoh, Dimitrios Balalis, Dimitrios Korkolis, Georgios Gkiokas, Eirini Pantiora, Theodosios Theodosopoulos, Argyrios Ioannidis, Konstantinos Konstantinidis, Sofia Konstantinidou, Nikolaos Machairas, Anna Paspala, Anastasia Prodromidou, Christos Chouliaras, Konstantinos Papadopoulos, Ioannis Baloyiannis, Ioannis Mamaloudis, George Tzovaras, Ioanna Akrida, Maria-Ioanna Argentou, Stylianos Germanos, Evangelos Iliopoulos, Ioannis Maroulis, George Skroubis, George Theofanis, Christos Chatzakis, Orestis Ioannidis, Lydia Loutzidou, Vasileios Kalles, Panagiotis Karathanasis, Nikolaos Michalopoulos, Charalampos Theodoropoulos, Dimitrios Theodorou, Tania Triantafyllou, Zoe Garoufalia, Natasha Hasemaki, Michalis Kontos, Gregory Kouraklis, Stylianos Kykalos, Theodore Liakakos, Eustratia Mpaili, Alexandros Papalampros, Dimitrios Schizas, Athanasios Syllaios, Ekaterini Christina Tampaki, Antonios Tsimpoukelis, Maria Ioanna Antonopoulou, Eirini Deskou, Dimitrios K. Manatakis, Dimitrios Papageorgiou, Menelaos Zoulamoglou, Christos Anthoulakis, Michalis Margaritis, Nikolaos Nikoloudis, Veronica Campo, André Ceballos, Mario-Andrés Flores, Waleska Giron, Donghyun Ko, Gabriel Martinez, Gustavo Recinos, Verónica Rivera Lara, Nataly Rueda, Andres Sanchez, Jorge Carlos Guillermo Tejeda Garrido, Maria-Lorena Aguilera-Arevalo, Alvaro Eduardo Alvarez Rivera, Elvis Benjamin Bamaca Ixcajoc, Lilian Elizabeth Barreda Zelaya, Patricia Chacòn-Herrera, Ligia Margarita Corea Ruiz, Guillermo Echeverria-Davila, Mario Garcia, Danilo García, Edgar Fernando Gutiérrez Mayen, Noriega José, Nery Mazariegos, Diego Méndez, Michael Paniagua Espinoza, Zsolt Baranyai, David Bardos, Marton Benke, Kristof Illes, Balint András Kokas, Réka Szabó, Akhila Appukuttan, Anjitha Asok, Vijaykumar D.k, Kapil Malik, Praveen Ravishankaran, Ritesh Tapkire, Guru Moorthy, Joyner Abraham, Ramesh Muthuvel, John Alapatt, Abhay Kattepur, Nizamudheen Pareekutty, Mebanshanbor Garod, Caleb Harris, Cliff Wanniang, Ashish Gupta, Deepak Nehra, Sanjeev Parshad, Rajgopal Acharya, Rajendra Badwe, Manish Bhandare, Urvashi Jain, Karishma Kirti, Nita Nair, Shailesh Shrikhande, Purvi Thakkar, Premkumar Anandan, Archana C S, Arun Holenarasipur Narasannaiah, Tejaswi Jagarlamudi, Sunil Kumar Venkatappa, Rashmi M R, Mallikarjuna Manangi, Abhishek Raghavendra, K. Seshagiri Rao, Vinay S, Vinay Sajjan, Aneesh Shenoy, Santhosh Shivashankar Chikkanayakanahalli, Kavya Tharanath, Sushmita V, Peter Adidharma, Raksheeth Agarwal, Radhian Amandito, Phebe Anggita Gultom, Ghafur Rasyid Arifin, Matthew Billy, Zatira Elfizri, Alessa Fahira, Devi Felicia, Triana Hardianti Gunardi, Nadya Johanna, Nadia Rahmadiani Nugrahadi, Sonar Soni Panigoro, Siti Rahmayanti, Retta Catherina Sihotang, Santi Yuanita Brata, Hadi Winoto, Nastaran Barati, Manoochehr Karami, Hamidreza Khorshidi, Homa Naderifar, Mazin A. Abdulla, Maggie Coleman, Ronan J Doherty, Rob Hannon, Brenda Murphy, Aine Stakelum, Des Winter, Lylas Aljohmani, Richard Farnan, Yeshey Seldon, Tanna Tan, Shriya Varghese, Mohammad Alherz, Muaaz Ather, Mohammad Bajilan, Vivien Graziadei, Isobel Pilkington, Omar Quidwai, Paul Ridgway, Haaris Shiwani, Abd al-Rahman Tahir, Eimear Blunnie, Daniel Burke, Niall Kennedy, Kate Macdonagh, Maeve O'Neill, Siobhan Rooney, Giuseppe Falco, Guglielmo Ferrari, Simone Mele, Gabriela Elisa Nita, Lara Ugoletti, Maurizio Zizzo, Gianmaria Confalonieri, Giovanni Pesenti, Fulvio Tagliabue, Gianluca Baronio, Deborah Ongaro, Giacomo Pata, Bruno Compagnoni, Renato Salvadori, Lucio Taglietti, Nicola D'Alessandro, Pierpaolo Di Lascio, Giovanni Pascale, Luca Bortolasi, Tommaso Campagnaro, Massimo Carlini, Giorgio Lisi, Davide Lombardi, Corrado Pedrazzani, Domenico Spoletini, Giulia Turri, Paola Violi, Donato Francesco Altomare, Fabrizio Aquilino, Nicola Musa, Vincenzo Papagni, Arcangelo Picciariello, Leonardo Vincenti, Dario Andreotti, Savino Occhionorelli, Matteo Tondo, Stefano Maria Massimiliano. Basso, Paolo Ubiali, Riccardo Cirelli, Marco Enrico Mario Maino, Guglielmo Niccolò Piozzi, Emanuele Picone, Rosa Scaramuzzo, Giovanni Sinibaldi, Alfonso Amendola, Lorenzo Anastasio, Luigi Bucci, Emanuele Caruso, Antonio Castaldi, Sara Di Maso, Vincenza Paola Dinuzzi, Giovanni Esposito, Maria Gaudiello, Mariano Cesare Giglio, Paola Antonella Greco, Gaetano Luglio, Andrea Manfreda, Ester Marra, Federica Mastella, Gianluca Pagano, Roberto Peltrini, Vincenzo Pepe, Michele Sacco, Viviana Sollazzo, Giovanni Spiezio, Ettore Cianchetti, Nunzia Menduni, Michele Maria Carvello, Francesca Di Candido, Antonino Spinelli, Fabio Corsi, Luca Sorrentino, Fabio Marino, Emanuele Luigi Giuseppe Asti, Luigi Bonavina, Emanuele Rausa, Martina Asta, Andrea Belli, Francesco Bianco, Carmela Cervone, Paolo Delrio, Armando Falato, Andrea Fares Bucci, Rita Guarino, Ugo Pace, Daniela Rega, Emilia De Luca, Gaetano Gallo, Giuseppe Sammarco, Giuseppe Sena, Giuseppina Vescio, Letizia Santandrea, Giampaolo Ugolini, Davide Zattoni, Nicola Chetta, Gaetano Logrieco, Serafino Vanella, Gianluca Garulli, Nicola Zanini, Andrea Bondurri, Francesco Cammarata, Francesco Colombo, Diego Foschi, Giulia Maria Beatrice Lamperti, Anna Maffioli, Gianluca Matteo Sampietro, Al'ona Yakushkina, Gloria Zaffaroni, Luca Ansaloni, Enrico Cicuttin, Maria Grazia Sibilla, Harmony Impellizzeri, Marco Inama, Gianluigi Moretto, Sylvie Mochet, Elisa Ponte, Antonella Usai, Stefano Mancini, Andrea Sagnotta, Luigi Solinas, Elisa Bolzonaro, Nicolò Tamini, Gianluca Curletti, Raffaele Galleano, Michele Malerba, Sofia Campanella, Gianfranco Cocorullo, Francesco Colli, Paolino De Marco, Nicolò Falco, Tommaso Fontana, Leonel jospin Kamdem Mambou, Antonella La Brocca, Leo Licari, Brenda Randisi, Giovanna Rizzo, Giulia Rotolo, Giuseppe Salamone, Roberta Tutino, Paolina Venturelli, Stefano Malabarba, Alessandro Sgrò, Ivan Vella, Bruno Cirillo, Daniele Crocetti, Giorgio De Toma, Pierfrancesco Lapolla, Andrea Mingoli, Paolo Sapienza, Angela Belvedere, Stefania Bianchini, Margherita Binetti, Arianna Birindelli, Valeria Tonini, Mauro Podda, Fabio Pulighe, Michele De Rosa, Lorenzo Bono, Felice Borghi, Paolo Geretto, Maria Carmela Giuffrida, Corrado Lauro, Alessandra Marano, Luca Pellegrino, Paola Salusso, Diego Sasia, Michela Campanelli, Alberto Realis Luc, Mario Trompetto, Roberto Cardia, Nicola Cillara, Antonio Nicola Giordano, Antonio Costanzo, Mario Alessandro Giovilli, Luca Turati, Silvestro Canonico, Gianluca Pellino, Guido Sciaudone, Francesco Selvaggi, Lucio Selvaggi, Nader Albsoul, Ahmad AlBsoul, Ala'a Aldeen Alkhatib, Osama Alsallaq, Justin Z. Amarin, Rami Ayoub, Isam Bsisu, M S El Muhtaseb, Mohammad Jabaiti, Jamal Melhem, Ibrahim Nour, Yasmeen Z. Qwaider, Mohammad Hasan Salameh, Ahmad Suleihat, Haya H. Suradi, Mohammad Alammarin, Almoutuz Aljaafreh, Mohammad Bani hani, Zeina Bani hani, Farah Bani Hani, Toqa Fahmawee, Shadi Hamouri, Cyrine Katanani, Ra'fat Tawalbeh, Tamara Tawalbeh, Hassan Zawahrah, Mohamad K. Abou Chaar, Lana Abusalem, Mahmoud Al-Masri, Hani Al-Najjar, Lutfi Barghuthi, Zahra Ahmed, Adnan Maulana, Omar Ngotho, Charbel Kamau, Aruyaru Stanley Mwenda, Fridah Bosire, Elizabeth Mwachiro, Robert Parker, Ian Simel, Kimutai Sylvester, Abdulmunem Ahmed Mustafa Althini, Sofian Elbarouni, Aya Elseed Elbeshina, Ahmed Gwea, Ans Malek, Wedad Albashir Masoud Farag, Abdulwahab Abdalei, Abu Baker Abdel Malik, Areej Abo-khammash, Ma'aly Abuhlaiga, Nour Adnan, Marwa Albaggar, Asma Alfitory, Asma Aljanfi, Fakhruddin Almuzghi, Zohoor Altumei, Fatima Alzabti, Hana Ashoushan, Mohamed Assalhi, Joma Azzubia, Sondos Bnhameida, Malik Delhen, Houssein Elshafei, Hana Elteir, Fatima Esbaga, Abdel Aziz Gobbi, Fatma Hamouda, Hamdan Hilan, Rania Ismail, Fieruz Jebran, Muataz Kasbour, Galia Maderi, Saja Mohammad, Burooj Mohammed, Habib Murtadi, Hamassat Mustafa, Mohamed Rajab, Sarah Trenba, Mariam Wafaa, Eman Al Sagheir, Alabas Almigheerbi, Ahmed Alzahaf, Sumayyah Ghayth Bahroun, Najah Ben Dallah, Mahmoud Elshaibani, Haitem Eswaye, Maha Karar, Samah Omar, Eman Younes, Maha Younes, Dafer Zreeg, Saleh Abujamra, Firas Ashour, Mala Elgammudi, Wesal Omar F. Aljadidi, Enas Saddouh, Randa Sharif, Aya Alabuzidi, AbdulMawlay Alwerfally, Sarra Aribi, Fatma Bibas, Taha Elfaituri, Yasmine Elhajjaji, Ala Khaled, Wegdan Khalil, Tesneem Layas, Enas Soula, Ahmed Tarek, Muad fathi khalleefah Abu hallalah, Saleh Abujamra, Hazem Abdelkarem Ahmed, Tagwa Alsharef, Abdulsalam Ali Ben Saoud, Tasnim El Gharmoul, Ahmed Elhadi, Safa Elrais, Abdulhalim Shebani, Heba Zarti, Asaid Zeiton, Marijus Ambrazevicius, Nerijus Kaselis, Migle Stakyte, Oleg Aliosin, Agne Cizauskaite, Sarunas Dailidenas, Vitalijus Eismontas, Migle Kybransiene, Vitalija Nutautiene, Narimantas Samalavicius, Dainius Simcikas, Algirdas Slepavicius, Albinas Tamosiunas, Nerijus Ubartas, Paulius Zeromskas, Saulius Bradulskis, Edvinas Dainius, Juozas Juočas, Egle Kubiliute, Juozas Kutkevičius, Aurimas Opolskis, Audrius Parseliunas, Andrejus Subocius, Donatas Venskutonis, Egle Virbickaite, Diana Zuikyte, Algirdas Bogusevicius, Kristina Buzaite, Daiva Čepuliené, Ieva Cesleviciene, Vaidotas Cesna, Jolanta Gribauskaite, Povilas Ignatavicius, Mantas Jokubauskas, Monika Liugailaitè, Ernest Margelis, Ruta Mazelyte, Lina Pankratjevaitè, Matas Pažusis, Agne Rackeviciute, Justina Saladyte, Monika Škimelytè, Vygintas Šlenfuktas, Monika Sudeikyte, Algimantas Tamelis, Tomas Vanagas, Žygimantas Žumbakys, Aivaras Atkociunas, Audrius Dulskas, Justas Kuliavas, Justas Birutis, Sigitas Paškevičius, Mindaugas Šatkauskas, Donatas Danys, Matas Jakubauskas, Lina Jakubauskiene, Marius Kryzauskas, Vytautas Lipnickas, Gabija Makūnaitè, Fanjandrainy Rasoaherinomenjanahary, Herizo Rasolofonarivo, Luc Hervé Samison, Bitiel Banda, Precious Malemia, Vanessa Msosa, Ahmad Imran Ahmad Izzuddin, Andre Das, Ying Yee Gan, Tan Shong Sheng, Jia yng Siaw, Mohd Fadliyazid Ab Rahim, Dyg Zahratul Hamrak Abang Jamari, Nurfariza Che Husin, Muhd Yusairi Kamarulzaman, Yi Ping Lim, Nil Amri Mohamed Kamil, Mohd Razeen Mohd Hassan, Saidah Mohd Sahid, Johari Mustafa, Elaine Hui Been Ng, Wan Khamizar Wan Khazim, Ng Chang Ern, P.g. Lingeshan, Syariz Ezuan Sulaiman, Sue Ean Ang, Muhammad Navid Bin Mohamad Sithik, Yih Jeng Cheong, Mahadevan Deva Tata, Law Jia Xian, Aravinthan Kadravello, I-Ern Koh, Li-Yen Ng, Yuki Julius Ng We Yong, Kandasami Palayan, Chi Xuan Sam, Phuah Siow Jin, Jeremy Tan Ern Hwei, Yita Tang, Alvin Zubin Ter, Michael Pak-Kai Wong, Andee Dzulkarnaen Zakaria, Zaidi Zakaria, Fitjerald Henry, Thyivya Kalaiselvan, Muhammad Fairuz Shah Abd Karim, Mohamed Rezal Abdul Aziz, Nora Abdul Aziz, Tak Loon Khong, Peng Choong Lau, Hiong Chin Lim, April Camilla Roslani, Jonathan Chen Ken Seak, Sui-Weng Wong, Lai Fen Wong, Leow Yeen Chin, Mercy Chinemerem Anyanwu, Elaine Borg, Zachary Busuttil, Thomas Calleja, Kurt Lee Chircop, Ruth Cutajar, Andrew Michael Dimech, Sarah Ellul, Joseph Galea, Kiara Gascon Perai, Ruth Gatt, Lisa Kelman, Elizabeth Micallef, Favour Nwolu, Kim Sammut, Joanna Thompson, Sean Warwicker, Matthew Zammit, Fernando Cordera, Efraín Cruz González, Jorge Sánchez-García, Francisco José Barbosa Camacho, Francisco Javier Barrera López, Carlos Jose Zuloaga Fernandez del Valle, Eric Acosta, Iván Romarico González Espinoza, Perla Moreno, Ana Olivia Cortes-Flores, Clotilde Fuentes Orozco, Alejandro Gonzalez Ojeda, Samantha Corro Díaz González, Laura Martinez, Antonio Ramos-De la Medina, Bonifacio Mosqueda Amador, Armando Novoa, Dennet Arturo Olazo Espejo, Alejandro Jimenez, Federico Lopez Rosales, Elva Gabriela Vanoye, Luis Alberto Garcia Gonzalez, Roberto Carlos Miranda-Ackerman, Manuel Solano-Genesta, Alethia Alvarez-Cano, Hector Hugo Romero-Garza, Heriberto Medina-Franco, Lorelí Mejía-Fernández, Noel Salgado-Nesme, Omar Vergara-Fernandez, Guadalupe Montserrat Gutiérrez-Mota, Francisco Xavier Hernandez Vera, Anabella Llantada Lopez, Gilberto Morgan Villela, Felipe de Jesus Ramirez Padilla, Walezka Tapia Marin, Mónica Martínez Maldonado, Ramses Sánchez Suárez, José Manuel Troche, Chaymae Benyaiche, Oumaima Outani, Souadka Amine, Amine Benkabbou, Anass Mohammed Majbar, Raouf Mohsine, Ali Rafik, Thida Oung, Moe Moe Tin, David W Borowski, Philipp Plarre, David W Borowski, Philipp Plarre, Anna Alberga, Nina Sluiter, Jurriaan Tuynman, Robin Blok, Didem Cömert, Roel Hompes, Marianne Kalff, Merel Elisabeth Stellingwerf, Pieter Tanis, Mark van Berge Henegouwen, Elise Maria van Praag, Daan Wisselink, Michael Gerhards, Josephine Lopes Cardozo, Emma Westerduin, Joske de Jonge, Aaw van Geloven, Kaz van Schilt, Frank den Boer, Simone Stoots, Stijn Vlek, Jamie Adams, Ibrahim S. Al-Busaidi, Gabrielle Budd, Seung il Choi, Michael Jen Jie Chu, Anurag Ganugapati, Lucy McKinstry, Rebecca Pascoe, Simon Richards, Kenrick Rosser, Annie Stevenson, Rebecca White, Shebani Farik, Jin Kwun, Ahmed Murad, Sarah Cowan, Timothy Hall, Michael Hayton, Laminou Malam Sani, Souleymane Oumarou Garba, Harissou Adamou, Ibrahim Amadou Magagi, Oumarou Habou, Halima Aliyu, Muhammad Daniyan, Tunde T. Sholadoye, Lawal Abdullahi, Lofty-John Anyanwu, Aminu Mohammad Mohammad, Abubakar Bala Muhammad, Abdurrahman Abba Sheshe, Ibrahim Suleiman, Alaba Adesina, Ajibola Awolowo, Clement Onuoha, Omotayo Salami, Ogechukwu Taiwo, Agboola Taiwo, Stephen Kache, Jerry Godfrey Makama, Danjuma Sale, Olajide Abiola, Akinlabi Ajao, Anthony Ajiboye, Amarachukwu Etonyeaku, Julius Olaogun, Ademola Adebanjo, Opeoluwa Adesanya, Michael Olatunji Afolayan, Olanrewaju Balogun, Ayomide Makanjuola, Samuel Nwokocha, Rufus Wale Ojewola, Thomas Olagboyega Olajide, Adewale Aderounmu, Abdul-Rashid Adesunkanmi, Adewale Adisa, Augustine Agbakwuru, Adeleke Akeem Aderogba, Olusegun Isaac Alatise, Olukayode Arowolo, Oladejo Lawal, Tajudeen Mohammed, Chinedu Ndegbu, Olalekan Olasehinde, Funmilola Wuraola, Akinbolaji Akinkuolie, Amarachukwu Etonyeaku, Arinzechukwu Mosanya, Omobolaji Ayandipo, Peter Elemile, Taiwo Akeem Lawal, Samuel Ali SANI, Stephen Garba, Rebecca Hauwa SANI, Samson Olori, Henry Onyebuashi, Ifeanyi Umoke, Adedire Adenuga, Ademola Adeyeye, Olufemi Habeeb, Bashir Lawal, Abdulrasheed Nasir, Eirik Kjus Aahlin, Didrik Kjønås, Elisabeth Myrseth, Jibran Abbasy, Abdul Alvi, Omair Saleem, Asma Afzal, Anam Nazir, Muhammad Farooq, Ayesha Liaqat, Syed Asghar Naqi, Ali Raza, Muzna Sarfraz, Muhammad Sarwar, Muntaha Banglani, Ambreen Munir, Rahmat Sehrish, Bushra Ayub, Raza Sayyed, Amna Altaf, Saima Ayub, Ahmad Uzair Qureshi, Komal Saeed, Bilal Syed, Sana Amir Akbar, Abdul Wahid Anwer, Ruqayya Naheed Khan, Amina Iqbal Khan, Shahid Khattak, Sameen Mohtasham, Muhammad Asad Parvaiz, Aamir Ali Syed, Abdul Basit Ansari, Noman Shahzad, Tanwir Khaliq, Isbah Rashid, Shahzad Hussain Waqar, Hasan Abu Al-saleem, Amjad Abu Alqumboz, Mohammad Alqadi, Adham Amro, Rawan Assa, Eman Awesat, Rawan Ayyad, Mohammed Hammad, Ayat Haymony, Bassel Hijazi, Bara Hmeidat, Rowaa Lahaseh, Aseel Qawasmi, Alaa Rajabi, Mohammed Shehada, Sundus Shkokani, Yasmine Yaghi, Nadine Yaghi, Mohammad AlZohour, Mohammad Farid, Yousef Mahmoud Habes, Wesam Juba, Yanal Nubani, Abdelrahman Rabee, Mohammad Sa'deh, Saeed Abed, Iyad Al basos, Mohammad Alswerki, Dina Ashour, Israa Awad, Samar Diab, Alaa El Jamassi, Sahar El-Kahlout, Somaya Elhout, Ahmed N K Hajjaj, Doaa Hasanain, Baraa Nabil hajjaj, Mohammed Obaid, Eman Saikaly, Ahmed Salhi, Hiba Al-Tammam, Murad Almasri, Muath Baniowda, Doha Beshtawi, Ali Horoub, Rami Misk, Bayan Mohammad, Rami Qasrawi, Tasnim Sholi, Samar Abu-Nimeh, Abrar Abu-srour, Sadi A. Abukhalaf, Samer Adawi, Barah Alsalameh, Kholoud Ayesh, Muawiyah Elqadi, Ahmad Hammouri, Fatima Karim Mustafa, Natalie Marzouqa, Shatha Melhem, Dima Miqdad, Balqees Mohamad, Mhammed Rawhi, Ayman B. Abu Ahammala, Ahmed Abu Ataya, Israa Abu Jayyab, Samar Al-Shwaikh, Othman Alagha, Mohammed Alasttal, Haneen Awadallah, Mahmood Elblbessy, Jehad Fares, Akram Jarbou, Ibtisam Mahfouz, Moath A. Albahnasawi, Asmaa' Abo mahadi, Hasan Abuelhatal, Ayham Abuelqomboz, Abdelrahman Almoqayyad, Abdallah Alwali, Reem Balaawi, Mahmoud Hamouda, Mohammed Humeid, Abdullah Jedyan, Tasneem Mahmoud Abu hamam, Ghadeer Matar, Ali Salem, Tahani Samra, Nureddin Shaheen, Karam Shihada, Ayoob A.Nemer, Mahmoud Abu Al Amrain, Abdulwhhab Abu Alamrain, Najlaa Abu Jamie, Mohammed R. Abu-Rous, Nada Alfarra, Mohammed AlTaweel, Noor Alwhaidi, Ramadan Hamed, Bader Saqqa, Ahmad Shaheen, Dana Aljaber, Loay Aljaberi, Malak Alwaheidi, Assef Jawaada, Hani Khaldi, Rami Qahoush, Jalil Qari, Rana Saadeh, Ahlam Salim, Aseel Yacoub, Abbas Abbas, Rana Abu shua'ib, Baraa Abu Zainah, Mahmoud AbuSirrees, Basheer Babaa, Ola Barhoush, Asef Belal qadomi, Laith Daraghmeh, Reema Haji, Alaa Khatatbeh, Lana Khatib, Salsabeel Qarariah, Yara Quzmar, Khalil Safadi, Roqaya Salameh, Mohammad Hassan, Shifaa Herzallah, Loai Massad, Ahmed Nazzal, Ranin Nazzal, Dennis Escobar, Gustavo Miguel Machain V, Agustin Rodriguez Gonzalez, Jorge Emerson Chachaima Mar, Nathaly Olga Chinchihualpa Paredes, Vicente Cuba, Walter Lopez, Maria Milagros Niquen Jimenez, Nestor Alberto Sanchez Bartra, Olenka Sapallanay Ojeda, Diego Sequeiros, Andrea Toscano Pacheco, María Vergara, Sol Abarca, Rodrigo Alcorta, Giuliano Borda-Luque, Ivan Edward Eusebio Zegarra, Claudia Luján López, Mirella Marrufo, Cinthya Mogrovejo, Andrea Nomura, Yamile Rodríguez Angeles, Maitza Rosario Vidal Meza, Gabriela Zavala, José Neiser Castillo Arrascue, Jomara Caroline Hidrogo Cabrera, José Julio Mariano Larrea vera, Miguel Osorio, Edgar Alcides Ylatoma Díaz, Mark Anthony Fontanilla, Joseph Roy Fuentes, Anna Leah Salazar, Genieve Dominguez, Marc Paul Lopez, Shiela Macalindong, Mark Augustine Onglao, Arjel Ramirez, Marie Dione Sacdalan, Mayou Martin Tampo, Gemma Leonora Uy, Jeremiah Mangahas, Kenneth Yabut, Joannes Paul Cañete, Bernalynn Eris Cansana, Ernes John Castro, Maria Kaiserin Lipana, Manuel Francisco Roxas, Vlu Jean Zara, Maciej Chroł, Paula Franczak, Michał Orłowski, Piotr Budzyński, Andrzej Budzyński, Pawel Bury, Agata Czerwińska, Jadwiga Dworak, Jacek Dziedzic, Michał Kisielewski, Jan Kulawik, Anna Lasek, Piotr Major, Piotr Małczak, Marcin Migaczewski, Michał Pędziwiatr, Magdalena Pisarska, Dorota Radkowiak, Mateusz Rubinkiewicz, Anna Rzepa, Tomasz Skoczylas, Maciej Stanek, Katarzyna Truszkiewicz, Mateusz Wierdak, Marek Winiarski, Piotr Zarzycki, Anna Zub-Pokrowiecka, Piotr Kowalewski, Rafał Roszkowski, Maciej Walędziak, Miguel Tomé, Sara Patrocinio, Ines Guerreiro, Filipe Almeida, Xavier de Sousa, Nuno Monteiro, Maria Teresa Costa Santos, Daniela de Oliveira, Marta Lopes Serra, Daniela Morgado, Christian Neves, Ana Carolina Oliveira, Alice Pimentel, Sofia Silva, Márcia Carvalho, Lúcia Carvalho, Joana Magalhães, Leonor Matos, Tânia Monteiro, Carlota Ramos, Vanessa Santos, José Barbosa, Jose Costa-Maia, Vítor Devezas, Ana Fareleira, Cristina Fernandes, Diana Gonçalves, Henrique Mora, Marina Morais, Fabiana Silva de Sousa, Sara Catarino Santos, Ana Logrado, André Tojal, Edgar Amorim, Miguel F. Cunha, Ana Fazenda, João Pedro Melo Neves, Inês Isabel Sampaio da Nóvoa Gomes Miguel, Diogo Veiga, José Azevedo, Hugo Cardoso Louro, Mariana Leite, José Azevedo, Maria Bairos Menezes, Bárbara Gama, Diana Brito, Marta Cristina Cruz Martins, André Graça e Magalhães, Ana Catarina Longras, Rita Lourenço, Diana Matos, Luis Castro, Filipa Policarpo, Joana Romano, Mariana Leite, Cristina Monteiro, Diogo Pinto, Marina Duarte, Sónia Fortuna Martins, Mariline Oliveira, Diogo Galvão, Lisandra Martins, Anaisa Silva, Viorel Taranu, Bárbara Vieira, Jessica Neves, Simone Oliveira, Hugo Ribeiro, Margarida Cinza, Rosa Felix, Arnaldo Machado, Joana Oliveira, Joana Patrício, Rita Pedroso de Lima, Mário Pereira, Miguel Rocha Melo, Cristina Velez, Alberto Abreu da Silva, Mariana Claro, Daniel Costa Santos, Andreia Ferreira, Hugo Capote, Daniela Rosado, Filipa Taré, Oriana Nogueira, Miguel Ângelo, José Miguel Baiao, Andreia Guimarães, João Marques, Miguel Nico Albano, Marta Silva, Ana Valente da Costa, Teresa Vieira Caroço, Sara Almeida Braga, Ines Capunge, Marta Fragoso, João Guimarães, Bruno Pinto, João Ribeiro, Miguel Angel, Guilherme Fialho, Monica Guerrero, Filipa Campos Costa, Diogo Cardoso, Vasco Cardoso, Magda Alves, Inês Estalagem, Tiago Louro, Cláudia Marques, Rita Martelo, Miguel Morgado, Rita Canotilho, Ana Margarida Correia, Pedro Martins, Mariana Peyroteo, João Gomes, Rita Monteiro, Manuela Romano, Daniela Macedo Alves, Rita Peixoto, Catarina Quintela, Maria João Jervis, Débora Melo, André Pacheco, Valter Paixão, Vera Pedro, Joana Pimenta, João Pimenta de Castro, Ana Rocha, Mircea Beuran, Matei Razvan Bratu, Cezar Ciubotaru, Bogdan Diaconescu, Sorin Hostiuc, Ionut Negoi, Bogdan Stoica, Evgeny Anokhin, Georgy Kuznetsov, Giorgi Oganezov, Fedor Paramzin, Ekaterina Romanova, Valeryan Rutkovskii, Vasilii Rutkovskii, Mikhail Shushval, Mikhail Zabiyaka, Khasan Dzhumabaev, Valerii Ivanov, Zaman Mamedli, Sergey Achkasov, Artem Balkarov, Elnur Nabiev, Marat Nagudov, Evgeny Rybakov, Karina Saifutdinova, Oleg Sushkov, Armen Vardanyan, Ainhoa Costas-Chavarri, Lule Joseph, Isaac Ndayishimiye, JC Allen Ingabire, Ntirenganya Faustin, Alphonse Zeta Mutabazi, Jean Paul Mvukiyehe, Vizir J.P Nsengimana, Carine Uwakunda, Mohammad Monir Abbas, Nouf Akeel, Murad Aljiffry, Kholoud Awaji, Ali Farsi, Ghader Jamjoum, Ahmad Khoja, Ashraf Maghrabi, Nadim Malibary, Mohammed Nassif, Abdulaziz Saleem, Abdullah Sultan, Wail Tashkandi, Hanaa Tashkandi, Nora Trabulsi, Mouhamadou Bachir Ba, Adja Coumba Diallo, Abdourahmane Ndong, Vladica Cuk, Uroš Janković, Jovan Juloski, Sharon Zhiling Koh, Frederick Koh, Kuok Chung Lee, Kai Yin Lee, Sean Lee, Wei Qi Leong, Bettina Lieske, Su Ann Lui, Prajwala Prakash, Jan Grosek, Gregor Norcic, Ales Tomazic, Nicolas Fitchat, Robert Jaich, Devorah Wineberg, Modise Zacharia Koto, Daniella Baiocchi, Damian Clarke, Christina Johanna Steenkamp, Stephanie Van Straten, Sharon Bannister, Adam Boutall, Galya Chinnery, Anna Coccia, Angela Dell, Parveen Karjiker, Christo Kloppers, Nicholas Loxton, Tumi Mabogoane, Francois Malherbe, Eugenio Panieri, Shreya Rayamajhi, Richard Spence, Tirsa van Wyngaard, Claire Warden, T E Madiba, Yoshan Moodley, Nivashen Pillay, Savannah Brooks, Charlise Kruger, Lisa Hannah Van Der Merwe, Ferhana Gool, Maahir Kariem, Heather Bougard, Kathryn Chu, Nazmie Kariem, Fazlin Noor, Reantha Pillay, Leandi Steynfaardt, Lucía González González, José Miguel Marín Santos, Paula Martín-Borregón, Javier Martínez Caballero, Cristina Nevado García, Pastora Rodriguez Fraga, Gonzalo De Castro Parga, Maria Pilar Fernández Veiga, Lucía Garrido López, Hugo Infante Pino, Irene Lages Cal, Marta López Otero, Manuel Nogueira Sixto, Marta Paniagua García Señorans, Laura Rodríguez Fernández, Alejandro Ruano Poblador, Erika Rufo Crespo, Raquel Sanchez-Santos, Vincenzo Vigorita, Ester Alonso Batanero, Dorisme Asnel, Isabel Cifrian Canales, Elisa Contreras Saiz, Irene De Santiago Alvarez, Tamara Díaz Vico, Sebastian Fernandez Arias, Daniel Fernández Martínez, Carmen García Bernardo, Luis Joaquín García Flórez, Carmen Garcia Gutierrez, Manuel García Munar, Carlos Alberto Márquez Zorrilla Molina, Marta Merayo, José Luis Michi Campos, Maria Moreno Gijon, Jorge L. Otero-Diez, Jose Luis Rodicio Miravalles, Lorena Solar-Garcia, Aida Suárez Sánchez, Nuria Truan, Cristina Alejandre Villalobos, Yurena Caballero Díaz, Marta Jimenez, Dacil Montesdeoca, Antonio Navarro-Sánchez, Victor Vega, Juan Beltrán de Heredia, Zahira Gómez, Carlos Jezieniecki, Ana Patricia Legido Morán, Mario Montes-Manrique, Mario Rodriguez-Lopez, María Ruiz Soriano, Jeancarlos Trujillo Díaz, Andrea Vazquez Fernandez, Nuria Argudo, Miguel Pera, Laia Torrent Jansà, Melody García Domínguez, Ignacio Goded, Marta Roldón Golet, Issa Talal El-Abur, Alejandra Utrilla Fornals, Vanesa Zambrana Campos, Maria Del Mar Aguilar Martinez, Marina Bosch, Luis García-Catalá, Luis Sánchez-Guillén, Eva Artigau, Nuria Gomez Romeu, David Julià Bergkvist, Beatriz Espina Perez, Olga Morató, Carles Olona, Beatriz Diéguez, Alexander Forero-Torres, Manuel Losada, Segundo Gomez-Abril, Paula Gonzálvez, Rosario Martinez, Sergio Navarro Martínez, Carmen Payá-Llorente, Álvaro Pérez Rubio, Sandra Santarrufina Martinez, Juan Carlos Sebastián Tomás, Ramon Trullenque Juan, Alberto Gegúndez Simón, Paloma Maté, Maria Isabel Prieto-Nieto, Ines Rubio-Perez, Aitor Urbieta, Marina Vicario Bravo, David Abelló, Matteo Frasson, Alvaro Garcia-Granero, Alfredo Abad Gurumeta, Ane Abad-Motos, Elena Lucena-de Pablo, Beatriz Nozal, Javier Ripollés-Melchor, Rut Salvachúa, Esther Ferrero, Luis Garcia-Sancho Tellez, Irene Ortega Vázquez, Antonio L. Picardo, Jose Alberto Rojo López, Laura Patricia Zorrilla Matilla, Carmen Cagigas Fernandez, Sonia Castanedo Bezanilla, José Estevez Tesouro, Maria Jose Fernandez-Diaz, Juan García Cardo, Marcos Gomez Ruiz, Erik Gonzalez-Tolaretxipi, Jaime Jimeno Fraile, Cristobal Poch, Montserrat Rodriguez-Aguirre, Noemí Troche Pesqueira, Maria Soledad Trugeda-Carrera, Javier de la Torre, Ruth Blanco-Colino, Eloy Espin-Basany, Martin Espinosa-Bravo, Clara Morales Comas, Eduardo Reyes Afonso, Joaquín Rivero Déniz, Christian Siso Raber, Mireia Verdaguer Tremolosa, Pramodh Chandrasinghe, Sumudu Kumarage, Nimeshi Wijekoon Arachchilage, Kithsiri Janakantha Senanayake, Ahmed Abdalla Ahmed Elkamel, Mohammed A. Adam, Mahmoud Saleh, Nina Blomme, Anders Thorell, Fredrik Wogensen, Andreas Älgå, Dhirar Ansarei, Fuat Celebioglu, Göran Heinius, Linda Nigard, Emil Pieniowski, Sandra Ahlqvist, Ida Björklund, Yucel Cengiz, Andreas Frånberg, Martina Håkansson, Karin Adamo, Oskar Franklin, Malin Sund, Rebecca Wiberg, Yvette Andersson, Abbas Chabok, Maziar Nikberg, Alexander Kugelberg, Claudia Canonica, Dimitrios Christoforidis, Fabrizio Fasolini, Paolo Gaffuri, Mauro Giuliani, Francesco Meani, Sotirios Georgios Popeskou, Silvia Pozza, Wiebke Wandschneider, Lorenz Peterer, Lukas Werner Widmer, Bernd Zimmermann, Panagiotis Bakoleas, Iris Chanousi, Lydia Charalampidou, Lukasz Filip Grochola, Franziska Heid, Sotirios Ntaoulas, Michail Outos, Georgios Peros, Hanna Podolska-Skoczek, Katharina Beate Reinisch, Christian Zielasek, Daniel Clerc, Nicolas Demartines, Jérôme Gilgien, Amaniel Kefleyesus, Pénélope St-Amour, Arnaud Toussaint, Maryam Alhimyar, Bayan Alsaid, Amr Alyafi, Ahmad Alkhaledi, Basel Kouz, Ahmad Omarain, Yusra Al-Sabbagh, Haya Alkhatib, Samer Sara, Ahmad Alhaj, Aghyad Danial, Lama Kadoura, Sarah Maa Albared, Yamen Monawar, Louei Nahas, Barook Abd, Ahmad Saad, Habib Wakkaf, Hanen Bouaziz, Hatem Bouzaiene, Montassar Ghalleb, Elif Akaydin, Ata Cem Akbaba, Onur Atakul, Ege Baltaci, Sevval Besli, Gökçen Burgu, Ulukan Cenal, Cansu de Muijnck, Hasan Can Demirkaya, Alper Dogruoz, Zeynep Ipek Gezer, Yasemin Gündoğdu, Merve Kara, Hasan Kürşad Korkmaz, Gökalp Kağan Kurtoğlu, Volkan Ozben, Berk Baris Ozmen, Ahmet Murat Pektaş, Eda Kübra Sel, Nilüfer Yenidünya, Fuat Baris Bengur, Berke Mustafa Oral, Tahir Koray Yozgatli, Seymur Abdullayev, Mehmet Emin Gunes, Nuri Alper Sahbaz, Tuba Banaz, Kübra Kargici, Omer Faruk Kuyumcu, Erkan Yanikoğlu, Merve Yeşilsancak, Duygu Yilmaz, Melik Kagan Aktas, Ahmet Rencuzogullari, Arda Isik, Sezai Leventoğlu, Ali Yalçinkaya, Osman Yüksel, Mustafa U Kalayci, Yasin Kara, Inanc Samil Sarici, Alp Akin, Gökçe nur Alemdağ, Ekin Arslan, Bahadir Emre Baki, Muhammed Selim Bodur, Adnan Calik, Bahar Candas Altinbas, Irem Cihanyurdu, Oğuz Erkul, Burak Gül, Ali Guner, Beyza Köse, Anil Semiz, Şule Sevim, Serkan Tayar, Kadir Tomas, Ozan yavuz Tüfek, Serdar Türkyilmaz, Mehmet Uluşahin, Arif Usta, Reyyan Yildirim, Sertaç Ata Güler, Ozan Can Tatar, Ecenur Varol, Busenur Kirimtay, Muhammed Uysal, Alp Yildiz, Emin Kose, Ahmet Burak Ciftci, Elif Çolak, Huseyin Eraslan, Gultekin Ozan Kucuk, Kürşat Yemez, Herman Lule, Mumbere Bienfait, Herman Lule, Emmanuel Bua, Matthew Doe, Noella Okalany, Arianna Birindelli, Maksym Basarab, Oleksii Bielosludtsev, Maryna Freigofer, Kateryna Kolhanova, Kateryna Perepelytsia, Kateryna Romanukha, Dmytro Savenkov, Stanislav Siryi, Maksym Tereshchenko, Nezamai Viacheslav, Anton Volovetskyi, Andrey Kebkalo, Yegor Tryliskyy, Volodimir Tyselskiy, Eilidh Bruce, Bing Lun Chow, Emma Iddles, Sarah McGuckin, Nicola Newall, George Ramsay, Parivrudh Sharma, Caitlin Stewart, Jeremy Wong, Abdul Badran, Michael Bath, Fanny Belais, Eman Butt, Kaustuv Joshi, Milan Kapur, Mike Shaw, Adam Townson, Christopher Yee Khang Williams, Timothy Gray, Robert Greig, Mansoor Husain, Elspeth Murray, Ahmed Mustafa, Ashar Asif, Arya Gokul, Max Shah, Mabel Temisanren Akitikori, Alexandros Charalabopoulos, Sophie Davidson, Sinead McNally, Shamil Rupani, Fatema Juma, Sarah Catherine Mills, Laura Muirhead, Kate Sellars, Una Walsh, Oliver Warren, Alice Chambers, Richard Hunt, Ella Teasdale, Stephen Boyce, Hannah Cornwall, Isabel Tol, Eleftherios Orestis Argyriou, Nicola Eardley, Meical Povey, Joanna M S Aithie, Ahmer Irfan, Mari-Claire McGuigan, Robert Starr, Craig Russell Warren, Jess Archibald, Georgia Kirby, Ivan Kisyov, Chun Kheng Khoo, Rachel Lee, Dana Photiou, Rowan Davis, Uday Prasad, P Zichu Yang, Jonathan Bird, Edmund Leung, Virginia Summerour, Chelise Currow, Jianshen Kiam, Gerald Jack Soon Tan, Anitha Muthusami, Ibifunke Pegba-Otemolu, Tomas Urbonas, Joseph Nunoo-Mensah, Edgaras Smolskas, Alex Boddy, Gianpiero Gravante, David Hunter, David Andrew, Amanda Koh, Amari Thompson, Lawrence Adams, Hollie A Clements, Kasun De Silva, Ogbonnia Ekpete, Seraj Haque, Scott Henderson, Bilal Ibrahim, Thummini Jayasinghe, Jennifer Livie, Keir Mailley, Gopikrishnan Nair, Daniel Tan, Caitlin Baggaley, Aleksander Dawidziuk, Bartosz Szyszka, Charlotte Barter, Nirav Gandhi, Karen Hassell, Samantha Hitchin, Jennett Kelsall, Eva Nagy, Ashrafun Nessa, Lisa Whisker, Fady Yanni, Mahmoud Ali, Deeksha Arora, Sunanda Hediwattege, Navam Kumarasinghe, Munir Rathore, Athula Tennakoon, Syed Mustafa Ali Ahmad, Oreoluwa Bajomo, Fahema Nadira, Valerio Celentano, Aneel Bhangu, James Glasbey, Ewen Griffiths, Rama Santhosh Karri, Jason Kei Chak Mak, Dmitri Nepogodiev, Michelle Pipe, Muhammad Iqbal Bhatti, Mohamed Rabie, Connor Boyle, David Hamilton, Aishath Mihuna, James Chean Khun Ng, Gary Nicholson, Agata Oliwa, Robert Pearson, Anna Rose, Shun Qi Yong, Catherine Boereboom, Michael Hanna, Catherine Walter, Thomas Samuel Greensmith, Rachel Mitchell, Eimear Monaghan, James Crawford, Susan Moug, James Blackwell, Hannah Boyd-Carson, Philip Herrod, Omar Al-Allaf, Miriam Beattie, Cameron Bullock, Shivang Burman, Gemma Clark, Nicolas Flamey, Oliver Flannery, Alexander Harding, Ben Kodiatt, Samuel Lawday, Shivani Mahapatra, Navin Mukundu Nagesh, Michael Ng, Dupinderjit Rye, Andrel Yoong, Laura Clark, Chris Deans, Monisha Edirisooriya, Cameron J Fairfield, Ewen M Harrison, Emma Victoria Carrington, Tsz Lun Ernest Wong, Baasil Yusuf, Carla Chamberlain, Kathryn Duke, Elizabeth Kmiotek, Azel Botes, Natalie Condie, Timothy Schrire, Reena Shah, Iolo Thomas-Jones, Charlotte Yates, Natasha Anthony, Edward Matthews, Kapil Sahnan, James Tankel, Sally Tucker, Jasmine Winter Beatty, Paul Ziprin, William Duggan, Anastasia Kantartzi, Shruthi Sridhar, Rachel Alys Khaw, Prakhar Srivastava, Charlotte Underwood, Homero Alves do Canto Brum, Sharat Chopra, Laura Davis, Rebecca Hughes, Joshua Tulley, Justin Alberts, Thomas Athisayaraj, Mojolaoluwa Olugbemi, Kasim Ahmad, Claudia Chan, Gavin Chapman, Hannah Fleming, Benjamin Fox, Julia Grewar, Kate Hulse, Duncan Rutherford, Mackay Sinead, Scott Smith, Doug Speake, Peter G Vaughan-Shaw, Natasha Christodoulides, Simrit Kudhail, Matthew Welch, Syed Muhibullah Husaini, Simon Lambracos, Chikamuche Anyanwu, Rishi Suresh, Jimmy Scott Thomas, Elizabeth Gleeson, Rebecca Platoff, Areeba Saif, Zachary Enumah, Eric Etchill, Alodia Gabre-Kidan, Mitchell Bernstein, Francesco Maria Carrano, Joseph Connors, Patricio Lynn, Marcovalerio Melis, Elliot Newman, Deshka S Foster, Kenneth Perrone, Ashley Titan, Thomas G Weiser, Sarwat Ahmad, Andrea Chao M.D. Bafford, Marco Dal Molin, Nader Hanna, Syed Nabeel Zafar, Mark Hemmila, Lena Napolitano, Jane J Wong, Julia Chandler, Lauren Wood, Sherry Wren, Taylor Ottesen, Lucia You, Kristin Yu, María del pilar Arciénega Yañez, Martin Ferreira Fernandes, Daniel González, Santiago Cubas, María Catalina González, Vanessa Zubiaurre, Rodrigo Demolin, Nicolas Giroff, Pablo Sciuto, Maite Campos, Gabriela Rodríguez Cantera, Gabriela Wagner, Garg Deepika, Mayaba Maimbo, Elliot Simuchimba, Anadi Bulaya, Chali Chibuye, Bright Chirengendure, Mary-Rose Kabale, Kizito Kabongo, David Linyama, James Munthali, Oliver Mweso, Francis Pikiti, James Otieno, Erick Chan, Log Tung Lai, Brighid Blackman, Sophie Richards, Suren Subramaniam, Rafid Karim, Nathan Kok, Yanni Dion Lee, Shabina Ali, Aanjaneya Sinha, Robert Corrigan, Nicole Barnes, Florence Wong, Grace Dennis, Julia Jedamzik, Emil Phillips, Wivine Piette, Marie Van hentenryck, Houenoukpo Koco, Souliath Lawani, Mamo Woldu Kassa, Tainá Santos Bezerra, Petar Gribnev, Dobromir Dimitrov, Panche Krastev, Sovannarith Oum, Divine Tim Bonghaseh, Maryam Al Farsi, Nourah Alsharqawi, Arnav Agarwal, Veronica Acevedo, Andrea Carolina Castillo Barbosa, Felipe Giron, Jimmy Paul Leon Rodriguez, Darko Kučan, Damir Rosko, Neven Barsic, Domagoj Župan, Amgad Hegazi, Vendula Trunčíková, Vladimir Fryba, Mostafa Mohamed, Ahmed Sultan, Ahmed Nagi, Abdallah Rashad Temerik, Mohamed Elemam Elshawy, Moustafa Ibrahim Mahmoud, Shrouk Omar, Mohamed Anwar, Tarek Rageh, Aya Elmokadem, Khaled Gaballa, Sandra Teppo, Antti Turunen, Pasi Pengermä, Quentin Ballouhey, Damien Bergeat, Ariane Weyl, Elisabeth Hain, Adam Gyedu, Edwin Yenli, Dorcas Osei-Poku, Vaia-Aliki Rompou, Athanasios Zoikas, Apostolos Gaitanidis, Georgios Koukis, Konstantinos Perivoliotis, Panagiotis Tavlas, Konstantinos Galanos-Demiris, George Zografos, Ioannis Karavokyros, Georgia Xanthopoulou, Eirini Iordanidou, Fernanda Ayau, Allan Garcia, Pekli Damján, Deepender Wason, Ashika B L, Ervandy Rangganata, Prerna Kamath, Donal B O'Connor, Margherita Pinto, Fabrizio Perrone, Francesca Paola Tropeano, Francesca Troilo, Daniela Bossi, Dario Scala, Lucrezia Pulitanò, Marcella Carella, Andrea Pietrabissa, Alice Gori, Giorgio Giraudo, Veronica De Simone, Alfio Alessandro Russo, Bartolomeo Braccio, Raed Al-Taher, Sarah Athamneh, Andrea Parker, Adnan Sawiee, Amina Kattia, Malik Salem, Osama Tababa, Zuhour Shaeeb, Vilius Syminas, Jonas Jurgaitis, Gytè Damulevičienè, Saulius Svagzdys, Tomas Poskus, Narindra Njarasoa Mihaja Razafimanjato, Ling Chieng Loo, Ing Ching Tiong, Wan Farahiyah Wan Muhmad, Harinthiran Vijeyan, Teoh Li Ying, Gabriella Grech, Rodrigo Arrangoiz, Vania Brickelia Jimenez Ley, Daniel Arizpe, Vania Brickelia Jimenez Ley, Elizabeth Lagunes Lara, Elizabeth Victoria Castro López, Jose Eaazim, Marije Gordinou de Gouberville, Vivian Bastiaenen, Simone Rottier, Fouad Nahab, Maria Yeonhee Ji, Mohammed Seyoji, Callistus Nwachukwu, Okechukwu Emeghara, Sayyid Egbunu Muhammed, Ayodeji Idowu, Olamiposi Sowemimo, Olakayode Ogundoyin, Oluwatosin Akande, Alexander Lott, Maliha Nadeem, Ahsan Ali Laghari, Asif Loya, Hassan Mushtaq, Muhammad Tariq Abdullah, Baseel Abuhilal, Mohammad Atawneh, Hamdan Hamdan, Belal Alhabil, Abedelrahman Srour, Ibrahim Mousa, Luis Da Silva Medina, Marie Dione Sacdalan, Marie Carmela Lapitan, Marie Dione Sacdalan, Marie Dione Sacdalan, Katarzyna Bartosiak, Pedro Ferreira, Vítor Francisco, Ricardo Lemos, Luísa Frutuoso, Sara Fernandes, Telma Fonseca, Jorge Pereira, Juan Rachadell, Ana Torre, Filipe Madeira Martins, Ana Cristina Carvalho, Joana Rodrigues Ferreira, Bruno Ribeiro da Silva, Helena Devesa, Ana Vieira, Inês Mónica, Margarida Amaro, Diogo Sousa, Marta Reia, João Louro, Ana Martins, Joaquina Dominguez, Inês Santos, Nuno Miguel Freitas Oliveira, José Carlos Pereira, Pedro Silva-Vaz, Ligia Freire, Ricardo Escrevente, Valentina Madalina Negoita, Dmitry Shakhmatov, Yves Nezerwa, Radosav Radulovic, Rachel Moore, Gareth Obery, Francois Viljoen, Tome Mendes, Antonio Suarez, Enrique Moncada, Maria Fernandez-Hevia, Carolina Curtis Martínez, Julia Maria Gil Garcia, Mariana González Zunzarren, Tarig Idris, Karolina Eklöv, Oskar Grahn, Leila Amin, Malin Blomqvist, Costanza Ajani, Rebecca Kraus, Nico Seeger, Melissa Willemin, Fadi Rayya, Mohammad Ayash, Raneem Msouti, Israa Kannas, Eias Abazid, Asil Esper, Skander Slim, Akil Serdar Kavcar, Erman Aytac, Ahmet Cem Dural, Ayse Ilker, Ismail Cem Eray, Eray Kurnaz, Saygin Altiner, Mustafa Deniz Tepe, Can Şahin, Evrim Savli, Aryon Innocent, Lilian Babirye, Andrii Diachenko, Vladislav Hordoskiy, Heather Curry, Charlene Yat Che Chau, Harry Robertson, Arin Mahmoud, Hannah Lennon, Lynette Loi, Emily Kirkham, Cameron McCann, Daniel Watts, Binay Gurung, Michael Wilson, Thomas Tribedi, Eleonora Garofalo, Baryab Zahra, Scott MacDonald, Ian Daniels, Nathan Ng, Shivun Khosla, James Olivier, Sum Yu Pansy Yue, Gayathri Suresh, Jack Wellington, Emmanuel Lorejo, Mafdi Mossaad, Yegor Tryliskyy, Madison Crutcher, Marjan Alimi, Ioana Baiu, Hossam Abdou, Alison Conway, Connor Peck, Gabriela Wagner, Mauro Andres Perdomo Perez, Ivan Trostchansky, Stanley Zulu, Mildred Nakazwe, Stephen R Knight, Thomas M Drake, Dmitri Nepogodiev, J Edward Fitzgerald, Adesoji Ademuyiwa, Philip Alexander, J.C. Allen Ingabire, Sara W Al-Saqqa, Bruce M Biccard, Giuliano Borda-Luque, David W Borowski, Sule Burger, Kathryn Chu, Damian Clarke, Ainhoa Costas-Chavarri, Justine Davies, Rachel Donaldson, Chikwendu Ede, O James Garden, Dhruv Ghosh, James Glasbey, T Peter Kingham, Hosni Khairy Salem, Theophilus Teddy Kojo Anyomih, Modise Zacharia Koto, Marie Carmela Lapitan, Ismaïl Lawani, Chiapo Lesetedi, Maria-Lorena Aguilera-Arevalo, Charles Mabedi, Mayaba Maimbo, Laura Magill, Felix Makinde Alakaloko, Alex Makupe, Janet Martin, Antonio Ramos-De la Medina, Mark Monahan, Rachel Moore, Vanessa Msosa, Soloman Mulira, Alphonse Zeta Mutabazi, Elmi Muller, Jospeh Musowoyo, Adewale O Adisa, Jean Léon Olory-Togbe, Riinu Pius, Ahmad Uzair Qureshi, Sarah Rayne, Tracey Roberts, Marie Dione Sacdalan, Catherine A Shaw, Neil Smart, Martin Smith, Richard Spence, Stephanie Van Straten, Stephen Tabiri, Viki Tayler, Thomas G Weiser, John Windsor, Joseph Yorke, Raul Yepez, Richard Lilford, Dion Morton, Aneel Bhangu, Sudha Sundar, Ewen M Harrison, Emmy Runigamugabo, Azmina Verjee, José Chen, Leonid Daya, Nouhaila El Aroussi, Valeria Farina, Tchianze Gnintedeme Olivier, Mauricio Gonzales Nacarino, Aamr Hammani, Sarah Honjo, Rebecca Jacobs, Hitomi Kimura, Andrey Litvin, Mugisha Nkoronko, Ibrahim Nour, Jasson Javier Oscullo Yepez, Gianluca Pagano, Francesco Pata, Wei Pin Hung, Ankit Raj, Alina Romani Pozo, Muna Rommaneh, Samuel Chimbioputo Sassamela Fabiano, Camila Milagros Shiroma Gago, Sebastian Shu Yip, Abhishekh Srinivas, Chia-Yen Sung, Aswan Tai, Yener Cristyell Valle Aranda, Sara Venturini, Dominique Vervoort, Jean Wilguens Lartigue

## Abstract

**Background:**

Early death after cancer surgery is higher in low-income and middle-income countries (LMICs) compared with in high-income countries, yet the impact of facility characteristics on early postoperative outcomes is unknown. The aim of this study was to examine the association between hospital infrastructure, resource availability, and processes on early outcomes after cancer surgery worldwide.

**Methods:**

A multimethods analysis was performed as part of the GlobalSurg 3 study—a multicentre, international, prospective cohort study of patients who had surgery for breast, colorectal, or gastric cancer. The primary outcomes were 30-day mortality and 30-day major complication rates. Potentially beneficial hospital facilities were identified by variable selection to select those associated with 30-day mortality. Adjusted outcomes were determined using generalised estimating equations to account for patient characteristics and country-income group, with population stratification by hospital.

**Findings:**

Between April 1, 2018, and April 23, 2019, facility-level data were collected for 9685 patients across 238 hospitals in 66 countries (91 hospitals in 20 high-income countries; 57 hospitals in 19 upper-middle-income countries; and 90 hospitals in 27 low-income to lower-middle-income countries). The availability of five hospital facilities was inversely associated with mortality: ultrasound, CT scanner, critical care unit, opioid analgesia, and oncologist. After adjustment for case-mix and country income group, hospitals with three or fewer of these facilities (62 hospitals, 1294 patients) had higher mortality compared with those with four or five (adjusted odds ratio [OR] 3·85 [95% CI 2·58–5·75]; p<0·0001), with excess mortality predominantly explained by a limited capacity to rescue following the development of major complications (63·0% *vs* 82·7%; OR 0·35 [0·23–0·53]; p<0·0001). Across LMICs, improvements in hospital facilities would prevent one to three deaths for every 100 patients undergoing surgery for cancer.

**Interpretation:**

Hospitals with higher levels of infrastructure and resources have better outcomes after cancer surgery, independent of country income. Without urgent strengthening of hospital infrastructure and resources, the reductions in cancer-associated mortality associated with improved access will not be realised.

**Funding:**

National Institute for Health and Care Research.

## Introduction

Of the 15·2 million individuals diagnosed with cancer in 2015, 80% required surgery.^1^ For many common, high-burden cancers, including breast, colorectal, and gastric cancers, surgery often offers the best chance of cure, particularly in early-stage disease. 45 million surgical procedures are estimated to be needed worldwide each year to treat cancer, yet fewer than 25% of patients with cancer have access to safe, affordable, and timely surgery.^2^

To address the growing cancer burden in low-income and middle-income countries (LMICs), investments will need to be made in the entire cancer care continuum. This includes surgical treatment for cancer and the services that support high-quality surgical care, such as diagnostic imaging, pathology, perioperative care, and the training of personnel. Investing in cancer care can yield substantial health and economic benefits if investments are closely aligned with country needs.^3^ Although a compelling rationale for investing in the global scale-up of cancer care exists, these data are predominantly based on simulation and extrapolation.^1^, ^3^, ^4^ Little is known about the type or quality of surgical care that patients with cancer receive for common, high-burden cancers around the world, nor the impact of surgical care on survival outcomes. These knowledge gaps make it difficult for countries to identify areas of need and make informed investments in their cancer systems in order to maximise health gains.

We previously showed^5^ that patients in LMICs have higher mortality after cancer surgery **c**ompared with those in high-income countries, but the impact of hospital facilities on patient outcomes was not explored. Structural characteristics such as case volume, facility availability, and the presence of specialised services are known to affect surgical outcomes in high-income settings.^6^, ^7^, ^8^ Improving hospital facilities through additional infrastructure and resources, translating to greater capacity, is thought to affect clinical outcomes in lower-income settings. Estimates suggest that poor-quality health systems cause 8 million deaths per year in LMICs.^9^

**Research in context**

**Evidence before this study**

Excess mortality after cancer surgery in low-income and middle-income countries (LMICs) has been described previously, but the effects of hospital facilities on early patient outcomes are unknown. Identifying the type and extent of these effects after cancer surgery worldwide is important to broaden understanding, guide further research, and inform national surgical plans. We reviewed the evidence for hospital infrastructure and resource availability on early outcomes following cancer surgery. We searched PubMed, MEDLINE, Google Scholar, and ClinicalTrials.gov for articles published between Jan 1, 1990, and May 10, 2021, using the terms “cancer” OR “malignancy” AND “surgery” AND “hospital” OR “characteristics” OR “facilities” AND “outcomes”, without language restrictions. The studies identified by our search largely focused on single tumour types and compared outcomes within single high-income countries. No studies explored the impact of hospital characteristics on outcomes after cancer surgery across different income settings.

**Added value of this study**

This study provides comprehensive data across income settings on the effect of hospital facilities on early outcomes in patients undergoing surgery for three common cancers. Even after case-mix adjustment, patients treated in hospitals with lower levels of hospital infrastructure and resources had higher postoperative mortality, despite similar complication rates. Excess mortality after surgery in these hospitals could be explained by the absence of these hospital facilities, which aid early identification and treatment of postoperative complications. The presence of five key hospital facilities is associated with a hospital's ability to perform safe elective operations for a broad range of cancers, highlighting their importance for access to high-quality, effective global surgical cancer care.

**Implications of all the available evidence**

We estimate that one to three early surgical deaths per 100 patients undergoing cancer surgery in LMICs can be prevented with improvements to hospital infrastructure and resources. These estimates could help policy makers to develop national cancer plans that include scaling up hospital cancer care facilities, together with the current focus on improving access to cancer services.

Using a systems-based approach, we aimed to describe critical surgical oncology services available worldwide and to investigate whether hospital facilities are associated with improved outcomes after cancer surgery worldwide, particularly in low-income settings, and the potential effects of improving these resources.

## Methods

### Study design and participants

A collaborative, international, multicentre, prospective, observational cohort study was conducted according to a prespecified, published protocol.^10^ The collaborative network methodology has been described elsewhere.^11^ Briefly, any hospital worldwide providing surgical services for breast, colorectal, or gastric cancer was eligible to take part, with centres collecting observational data on consecutive patients undergoing primary emergency or elective surgery for breast, gastric, or colorectal cancer between April 1, 2018, and Jan 31, 2019. Case ascertainment and data accuracy were high.^5^

The survey design followed a system-based approach, adapting the framework for comprehensive cancer centres in LMICs.^12^ Hospital infrastructure and process resources identified as core clinical service components to ensure access to high quality cancer care were captured, such as the presence of imaging modalities, oncology services, surgical treatment, and perioperative care (appendix pp 1–5). The ability of hospitals to perform elective operations for 11 globally prevalent cancers was also ascertained.^3^ 20 surgical experts across nine LMICs reviewed multiple survey iterations, with specific criteria to ensure included hospital facilities had relevance in low-income settings.

Definitions for each hospital facility were taken from WHO,^13^ if available, or the National Health Service data dictionary^14^ or American Association of Clinical Oncology^15^ (appendix pp 1–5). Members listed within the tumour board structure were taken from National Institute for Health and Care Excellence guidelines.^16^

Beta testing at two LMIC hospital sites was performed to ensure survey clarity before formal release across all collaborating hospitals. Collaborators at hospitals who had entered patient-level data for GlobalSurg 3 were invited to complete the hospital-level survey via a secure online link and entered directly onto the REDCap database. Collaborators were provided with a data extraction sheet to aid completion. The survey remained open for 8 weeks, until April 23, 2019, with reminders sent every 4 weeks if the survey remained incomplete. Hospitals were divided into clusters according to income group, with differential sampling across upper-middle and low to lower-middle clusters, where wide variation in hospital characteristics has been described.^9^ Independently collected patient-level observational data were then linked to hospital infrastructure and process data in this multimethod analysis.

A UK National Health Service Research Ethics proportionate review considered this study exempt from formal research registration (South East Scotland Research Ethics Service, reference NR/161AB6) because it was deemed a clinical audit. Individual centres obtained their own audit or institutional approval, together with ethical approval as per local regulations. This study is reported according to the Strengthening the Reporting of Observational Studies in Epidemiology (STROBE) guidelines.^17^

### Outcomes

The primary outcome measures were 30-day mortality and 30-day major complication, as defined by Clavien-Dindo grade III, IV, or V.^18^ Death was included in the definition of major complication and therefore was not a competing risk. Capacity to rescue was defined as the absolute risk difference of death in patients sustaining a major complication of surgery. Secondary outcome measures, as previously defined in the protocol,^10^ were selected as potential surrogate measures for patient safety and cancer care quality within hospitals. These included use of surgical safety checklists, negative resection margin rates, length of in-patient stay, readmission rates, use of a multidisciplinary tumour board meeting to discuss patient management, and follow-up method.^19^, ^20^ Patients were assessed at 30 days to determine postoperative outcomes, with follow-up done in person, by telephone, or by review of medical or readmission records, depending on local practices. Due to the differences in morbidity and mortality seen in the surgical management of breast cancer, a subanalysis including only patients with colorectal and gastric cancer was also performed.

### Statistical analysis

11 hospital facilities were selected a priori on the basis of their potential to directly or indirectly affect patient outcomes after cancer surgery.^3^, ^6^, ^7^, ^12^, ^21^, ^22^ These facilities were categorised into four areas potentially representing structure and process measures within the hospital that support the management of surgical patients at high risk:^6^, ^12^ imaging modalities (ultrasound and CT scan); oncological service organisation (oncologist, pathologist, and tumour board); perioperative care organisation (postoperative recovery area, opioid analgesia, palliative care, and critical care unit [high dependency unit, intensive care unit, or both]); and specialist cancer services (specialist hospital and ability to perform elective oesophagecetomy). The relations between elective oesophagectomy, facility availability, service complexity, and mortality are well described in high-income settings.^6^, ^7^, ^21^

Variable selection was performed to select hospital facilities associated with 30-day mortality using the Akaike information criterion, as described by Moons and colleagues.^23^ All hospital facilities were included as explanatory variables within this model, with the exclusion of patient-level data. Only main interactions were included to avoid overfitting. As a sensitivity analysis, a bootstrap procedure (n=5000) was performed to investigate variability in hospital facility selection. To obtain adjusted outcomes at hospitals with different numbers of facilities, we created an ordinal variable from selected variables, which represented the number of facilities at each hospital. Hospitals were then categorised into tertiles by patient distribution to define different facility levels.

Variation across different international health settings was assessed by stratifying countries by World Bank country group classifications. Differences between groups were tested with the Pearson χ^2^ test for categorical variables and with the Kruskal-Wallis test for continuous variables. To characterise the relation between hospital facilities and mortality, generalised estimating equations were constructed to account for income group, case mix (patient and disease factors), and operative characteristics known to be associated with worse outcomes after cancer surgery,^5^ with population stratification by hospital.

Adjusted outcomes were calculated as predicted probabilities from a generalised estimating equation logistic regression model, including potential confounders (patient age, sex, American Society of Anesthesiologists grade, Eastern Cooperative Oncology Group performance status, disease stage, and operative urgency) across income group and cancer type. We obtained 95% CIs and a p value for trend by fitting the generalised estimating equation logistic regression model with facility capability.

Sensitivity analyses for adjusted outcome rates were performed by imputing the average number of available hospital facilities by nearest neighbour human development index rank for missing hospitals. As an additional comparison, adjusted outcomes were also calculated using all 11 hospital facilities (ordinal value 0–11) across included hospitals using the same method.

The association between hospital facility level and 30-day mortality was calculated from logistic regression models for different covariate levels (patient and disease characteristics). Absolute risk differences and 95% CIs were calculated using bootstrap resampling (5000 draws). The number needed to treat to benefit was defined as the reciprocal of the absolute risk difference.

All p values were two-sided and were considered statistically significant if the p value was less than 0·05. All analyses were done using R (version 4.1.1), using finalfit, tidyverse, geepack, epitools, and bootStepAIC.

This study was prospectively registered with ClinicalTrials.gov, NCT03471494.

### Role of the funding source

The funder of the study had no role in study design, data collection, data analysis, data interpretation, or writing of the report.

## Results

**Figure 1:**
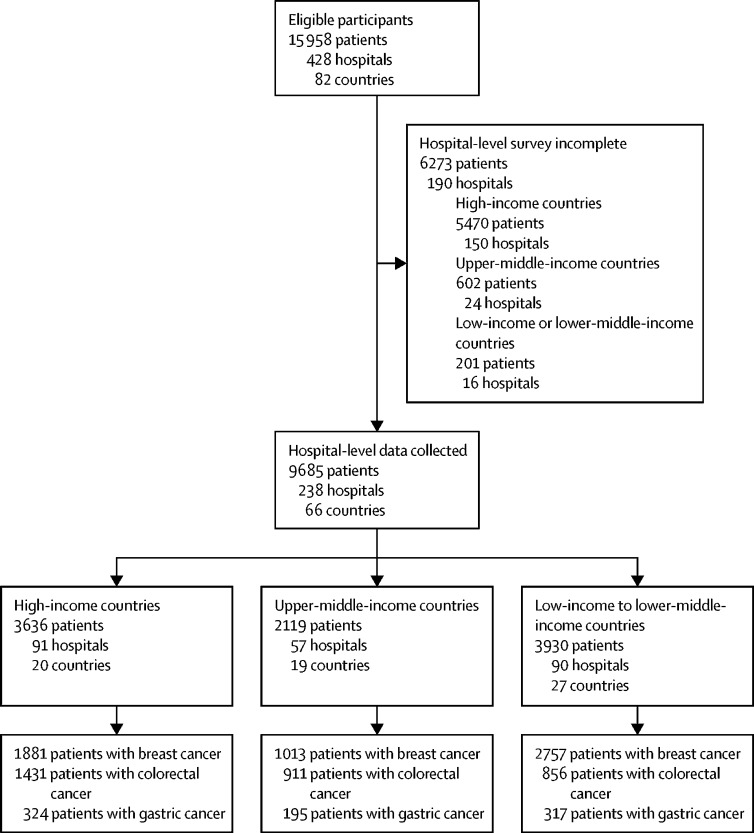
Study flowchart

Between April 1, 2018, and April 23, 2019, hospital-level data were collected with differential sampling across LMICs for 238 hospitals in 66 countries that surgically treated 9685 patients with cancer (91 hospitals in 20 high-income countries [3636 patients]; 57 hospitals in 19 upper-middle-income countries [2119 patients]; and 90 hospitals in 27 low-income or lower-middle-income countries [3930 patients]; figure 1). Incomplete surveys were due to non-responses across all income groups, rather than incomplete data submission. The characteristics of included hospitals by income group are summarised in table 1. Hospital facilities varied by income group except for the presence of ultrasound, pathology services, and performance of elective oesophagectomy.

**Table 1:** Distribution of hospital facilities by country income group

		**High (n=91)**	**Upper middle (n=57)**	**Low or lower middle (n=90)**	**Total (n=238)**	**p value**
Tumour board availability		89 (98%)	53 (93%)	71 (79%)	213 (89%)	0·0001
Oncologist available in hospital		85 (93%)	46 (81%)	63 (70%)	194 (82%)	0·0002
Palliative care available in hospital		68 (75%)	28 (49%)	37 (41%)	133 (56%)	<0·0001
Opioid medication available		84 (92%)	48 (84%)	47 (52%)	179 (75%)	<0·0001
Ultrasound available		77 (85%)	52 (91%)	75 (83%)	204 (86%)	0·38
CT scan available		87 (96%)	48 (84%)	54 (60%)	189 (79%)	<0·0001
Postoperative care facilities		86 (95%)	45 (79%)	62 (69%)	193 (81%)	<0·0001
Critical care bed available		84 (92%)	44 (77%)	60 (67%)	188 (79%)	0·0001
Pathology available in hospital		66 (73%)	46 (81%)	62 (69%)	174 (73%)	0·29
Hospital type						
	Non-referral hospital	25 (27%)	3 (5%)	5 (6%)	33 (14%)	0·0001
	Referral hospital	56 (62%)	46 (81%)	73 (81%)	175 (74%)	..
	Specialist cancer hospital	10 (11%)	8 (14%)	12 (13%)	30 (13%)	..
Elective oesophagectomy available		44 (48%)	34 (60%)	46 (51%)	124 (52%)	0·40

Data are n (%), unless indicated otherwise.

Elective procedures were similar across all income groups, with the exceptions of liver, pancreas, and rectal surgery (appendix p 6). The distribution of elective procedures stratified by the ability of a hospital to surgically treat breast, colorectal, and gastric cancer is shown in the appendix (p 6). A stepwise increase in all hospital facilities was seen as the total number of available facilities within a hospital increased (appendix p 7). Across colorectal and gastric cancer, unadjusted mortality rates reduced as overall hospital facility count increased (appendix p 8). For hospitals where hospital-level data were not available, case volume and adjusted mortality rates were found to be similar to rates in hospitals with hospital-level data available stratified by income group and cancer type (appendix pp 9–10).

**Figure 2:**
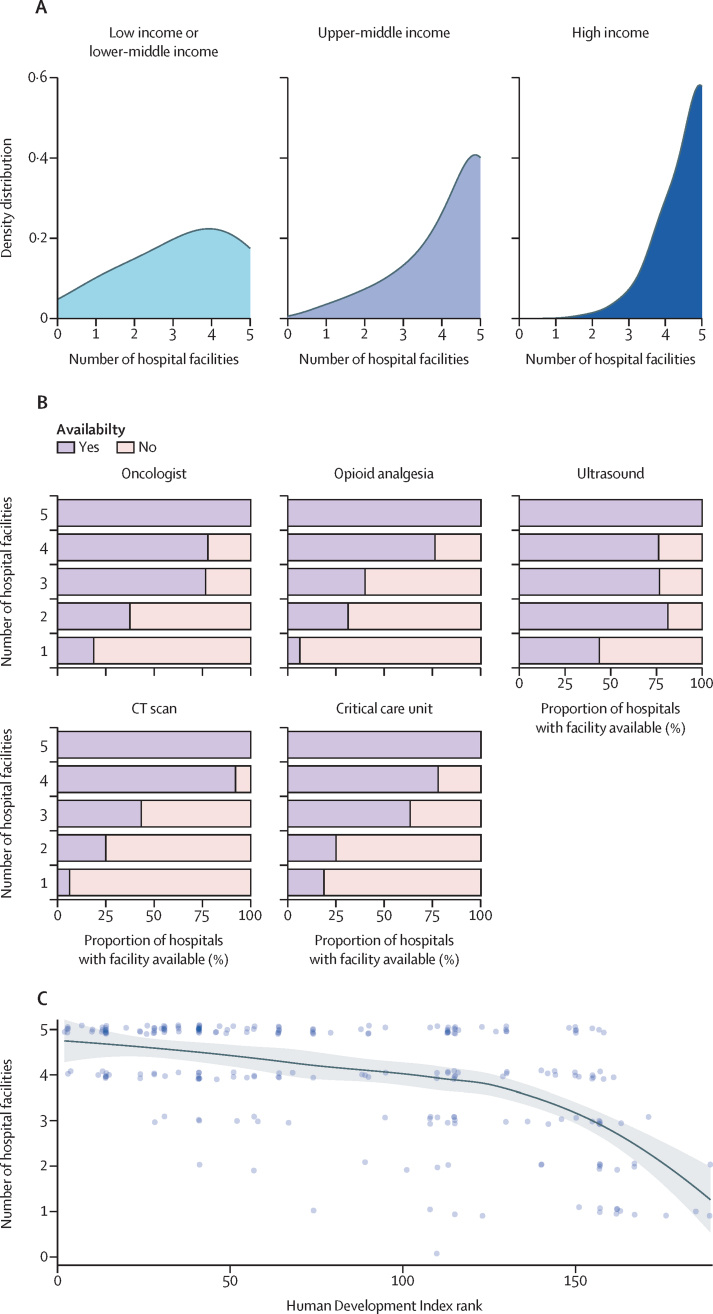
Distribution of hospital facilities by World Bank income group (A), individual hospital facility (B), and human development index rank (C)

Five hospital facilities were inversely associated with 30-day mortality and covered a broad range of resources (ultrasound, CT scanner, oncologist, opioid analgesia, and critical care unit; appendix p 11). The same five facilities were identified in a sensitivity analysis using bootstrap resampling (appendix p 12). Of the 238 hospitals included, 113 (47%) had all five of these hospital facilities present (figure 2). The number of available hospital facilities declined with worsening human development index rank, particularly in countries with a rank of more than 150 (figure 2C).

After categorisation by patient distribution, three hospital facility levels were identified (113 hospitals with five facilities available; 63 hospitals with four facilities; and 62 hospitals with three or fewer facilities). Patient distribution across the three hospital facility levels is shown in the appendix (pp 13–14). Patients at hospitals with three or fewer facilities were more likely to be from low-income settings and to present with colorectal or gastric cancer. These patients had poorer performance status, more advanced disease, and were more likely to require emergency surgery, with higher rates of postoperative surgical site infection (appendix p 15).

Hospitals with three or fewer facilities were less likely to use the surgical safety checklist (73·6% *vs* 83·7% for hospitals with more than three facilities; p<0·0001), to have a negative resection margin (87·5% *vs* 90·8%; p=0·0005), to review patients in clinic after discharge (45·6% *vs* 75·9%; p<0·0001), and to discuss patient management through a multidisciplinary tumour board (31·3% *vs* 78·3%; p<0·0001), and they had longer in-patient stays (5 days [IQR 3–9] *vs* 3 days [1–7]; p<0·0001; appendix pp 16–17). The availability of surgical treatment for several common cancer types was also reduced in hospitals with three or fewer facilities (appendix p 18).

After adjusting for patient and disease factors, 30-day mortality rates were higher in hospitals with three or fewer facilities across all cancers (3·7% *vs* 1·0% in hospitals with five facilities; OR 3·85 [95% CI 2·58–5·75]; p<0·0001; appendix p 19). No difference in adjusted mortality rates was seen in hospitals with four facilities available compared with those with five. A sub-analysis showed a similar finding in patients with colorectal and gastric cancer (6·9% *vs* 4·1%; 1·73 [1·18–2·52]; p=0·0063; appendix p 20).

Adjusted 30-day major complication rates were higher in hospitals with three or fewer facilities across all three cancers (11·8% *vs* 9·3% in hospitals with five facilities; OR 1·30 [95% CI 1·06–1·58]; p=0·011) and for patients with colorectal and gastric cancer (18·0% *vs* 13·5%; 1·40 [1·11–1·78]; p=0·0076; appendix p 21). After the development of a major complication, the capacity to rescue patients was significantly lower in hospitals with three or fewer facilities across all cancers (63·0% *vs* 82·7% in hospitals with five facilities; OR 0·35 [0·23–0·53]; p<0·0001; table 2) and for patients with colorectal and gastric cancer only (56·4% *vs* 71·5%; 0·51 [0·33–0·80]; p=0·0044). All effects persisted in a sensitivity analysis using an imputed dataset (appendix pp 23–26).

**Table 2:** Capacity to rescue patients after a major complication after case-mix adjustment, by number of hospital facilities

	**Hospitals, n (%)**	**Patients, n (%)**	**Adjusted capacity to rescue, % (95% CI)**	**Odds ratio (95% CI)**	**p value**
**All cancers (n=170)**					
Five facilities	86 (51%)	569 (65%)	82·7% (81·1–84·4)	1 (ref)	..
Four facilities	43 (25%)	173 (20%)	77·9% (74·6–81·3)	0·74 (0·49–1·13)	0·18
Three or fewer facilities	41 (24%)	134 (15%)	63·0% (58·4–67·6)	0·35 (0·23–0·53)	<0·0001
**Colorectal and gastric cancer (n=148)**					
Five facilities	73 (49%)	320 (58%)	71·5% (69·3–73·7)	1 (ref)	..
Four facilities	41 (28%)	119 (22%)	69·5% (65·5–73·5)	0·92 (0·58–1·45)	0·72
Three or fewer facilities	34 (23%)	110 (20%)	56·4% (51·8–60·9)	0·51 (0·33–0·80)	0·0044

Adjusted rates of capacity to rescue after major complication were calculated using generalised estimating equations to account for clustering of patients in hospital and for potential confounders (World Bank tertile, age, sex, cancer type, Eastern Cooperative Oncology Group performance status, American Society of Anesthesiologists grade, disease stage, and surgical urgency). 95% CIs and p values for trend were fitted using the multilevel logistic regression model with the number of available hospital facilities and all confounders as covariates.

**Figure 3:**
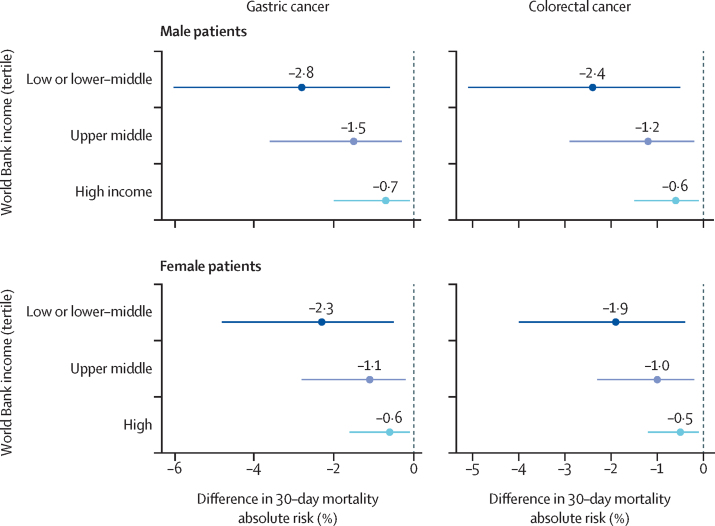
Absolute risk of 30-day mortality associated with four or more hospital facilities within each income group, stratified by cancer type and sex Estimates are shown for a patient of age 60 years, Eastern Cooperative Oncology Group performance status 1, American Society of Anesthesiologists grade 2, cancer stage III, and elective surgery. The grey dashed line represents three or fewer hospital facilities available and bars represent absolute risk of 30-day mortality with 95% CIs.

The absolute risk differences for 30-day mortality across hospital facility level were examined for common patient covariates in patients with colorectal and gastric cancer (figure 3; appendix p 26). The presence of four or more hospital facilities was associated with fewer deaths in the low-income to lower-middle-income group (two to three fewer deaths per 100 operations, number needed to treat 33–50), the upper-middle-income group (one to two fewer deaths per 100 operations, number needed to treat 50–100), and the high-income group (one fewer death per 100 operations, number needed to treat 100). Absolute differences across the three hospital facility levels are shown in the appendix (pp 27–29).

In a post-hoc analysis, we determined the absolute risk for 30-day mortality for higher-risk surgical patients, using common patient covariates for patients with an American Society of Anesthesiologists grade of 3 or higher (appendix pp 30–31). An increase in absolute risk difference was found across different levels of hospital facility in the low-income to lower-middle-income group (four to five fewer deaths per 100 operations, number needed to treat 20–25), the upper-middle-income group (two to three fewer deaths per 100 operations, number needed to treat 33–50), and the high-income group (one fewer death per 100 operations, number needed to treat 100).

## Discussion

In this prospective study of patients undergoing cancer surgery in 238 hospitals from 66 countries, higher availability of specific hospital infrastructure and resources was associated with improved outcomes. Hospitals that were well resourced had less than half the postoperative mortality rate, showing an improved ability to prevent death after the development of postoperative complications, with up to three fewer deaths per 100 operations performed. Of note, these findings were independent of country income group. The availability of hospital resources has long been thought to affect clinical outcomes in lower-income settings; the magnitude of this effect is now clear.

Despite the overall mortality benefit seen in hospitals with more resources and strong processes, many patients do not have access to such hospital infrastructure, particularly in low-income settings.^24^ Improvements to hospital facilities are known to be cost-effective,^3^ but the absence of high-quality data limits interpretability, and the effects of specific hospital facilities on outcomes and cancer surgery worldwide were previously unclear. Strategic planning requires detailed and accurate information to allocate appropriate resources, prioritise quality improvement, and evaluate effects. Determining the effectiveness of hospital infrastructure can guide future investment and provide a platform for continued assessment of hospital performance.

Our results offer a concrete approach by focusing on specific infrastructure and resources in hospitals worldwide. Such hospitals perform significantly better than others without them; in the 62 hospitals with three or fewer facilities, mortality rates were three times higher than in the 113 hospitals with all five facilities present. This difference is likely to be explained by a 50% increase in the capacity to rescue patients after the development of a major complication. These findings were robust in a sensitivity analysis and a similar trend was identified when all 11 hospital facilities were included. These results show that a strategy of expanding system capabilities at hospitals, particularly in low-income and middle-income settings, could markedly improve outcomes and patient access to safe, effective surgical care.

Previous studies have reported similar associations between key hospital facilities and mortality. Funk and colleagues^6^ found that the presence of complex medical oncology services and specific radiology services were important for lowering mortality in patients undergoing oesophagectomy. Similarly, Joseph and colleagues^25^ found that several institutional characteristics had a stronger effect on operative mortality after pancreatic resection than hospital volume. However, differences in major morbidity after surgery are often undescribed.^6^, ^21^

To our knowledge, this is the first global analysis to assess the impact of hospital facilities on short-term outcomes in cancer surgery. The synergistic effect of scaling up of imaging, treatment modalities, and quality in low-income settings on oncological outcomes has been shown in studies from 2021.^3^, ^4^ In particular, investments in imaging modality availability are a critical component for comprehensive improvement in global cancer survival.^3^

However, our results must be interpreted with caution. We suspect that these facilities are proxies for the expertise, resources, and complex processes of care required to facilitate surgery, including the optimisation of preoperative, intraoperative, and postoperative care for patients undergoing surgery for cancer. The presence of a CT scanner is unlikely to directly improve patient outcomes without associated investment in additional supportive capacity, such as health-care workers and technical support. The five key facilities that were included in our multivariable models are likely to be indirect markers for other structural and process measures that are also closely related to outcomes after cancer surgery. For example, we found that hospitals with more resources were more likely to use the WHO surgical safety checklist and have negative resection margins, potentially reflecting related organisational processes associated with these facilities. A similar pattern in outcomes was shown in models including all 11 of the hospital facilities originally assessed, suggesting that the five facilities identified in our analysis might also reflect further development of additional hospital services.

Higher levels of hospital facility were also associated with increased access to surgical care for a broad range of cancer types. The majority of hospitals with all five facilities present were able to perform elective operations for 11 different cancers, which represent 60% of all incident cancers and 70% of cancer deaths worldwide over the next 10 years.^3^ Patients also presented with earlier stage disease, suggesting hospital facility improvement could be associated with concurrent investment in early detection programmes and strengthening of health-care systems. Similar outcomes were found between hospitals with four or five key facilities, which could suggest a ceiling effect between expanding system capabilities and outcome improvement.

Centres providing cancer care worldwide vary in size, scale, and structure. Designated cancer centres, referral networks, and standardised cancer pathways are underdeveloped or absent in many LMICs.^26^ The centralisation of services into comprehensive cancer centres, supported by our analysis, is likely to improve quality of care, particularly in resource-constrained environments. However, centralisation can unintentionally reduce access to safe and effective cancer care, secondary to geographical and financial barriers for patients, particularly in the absence of robust referral mechanisms.^12^ Therefore, selection of a geographical location to serve the greatest number of patients, while defining the minimum requirements of a comprehensive cancer centre, is crucial.^26^ Efforts to improve the quality of cancer care must occur alongside efforts to increase access to care, to maximise health gains and develop equitable cancer systems.

Our study has important limitations. We have detailed hospital-level data for 55% of hospitals within the primary study, with a lower response rate from high-income hospitals. However, we covered 87% of patients in LMIC settings, where the majority of all cancer deaths occur.^27^ Furthermore, case volume and adjusted mortality rates of non-included hospitals were similar, and a sensitivity analysis indicated robust findings across all measured outcomes. Therefore, an association between missing responses and measured outcomes is unlikely. Despite including validated measures of overall patient health, we were unable to account for detailed patient comorbidity across income group within the adjusted models due to the burden of additional data collection, particularly in low-resource settings.

The five hospital facilities identified could represent additional, unmeasured structural and complex care processes. Despite capturing a broad range of hospital infrastructure and resources, we are unable to extrapolate our results to all the additional resources that a hospital might contain. However, as the number of hospital facilities increased, an increase in the capacity to rescue patients was shown. Therefore, investment and improvement in overall hospital capability is likely to greatly improve early patient outcomes after cancer surgery. However, in countries without universal health care, additional investment in hospital facilities must avoid unaffordable increases in total costs to patients for safe surgical care. Further work validating our findings and exploring the effect of specific combinations, particularly in LMIC settings, is required.

Additionally, we were unable to follow up patients beyond 30 days after surgery. Little is known about longer-term outcomes, such as cancer-free survival, in resource-limited settings.^1^, ^3^ Nevertheless, postoperative complications after major surgery can affect longer-term outcomes, including patient survival and disability.^22^ Longer-term disease and overall survival after surgery might be lower in LMICs, particularly because patients presented with later stage disease. The impact of delayed surgery in life-years lost for stage I–III disease is well described in high-income countries,^28^ but knowledge gaps exist globally. Furthermore, only patients undergoing primary surgery for breast, colorectal, and gastric cancers were included, and therefore our conclusions might not translate across other globally prevalent cancers. The current study will be extended to capture longer-term outcomes and other cancers in the future, which should add substantially to knowledge of the impact of hospital infrastructure and resources on global surgical outcomes.

Finally, we did not have information on surgeon volume or nurse-to-bed ratio, which are both known mediators in the association between hospital facilities and mortality.^25^ Debates are ongoing as to whether hospital volume versus hospital process is the primary reason for lower perioperative mortality in cancer surgery,^25^, ^29^ particularly because available clinical resources often increase with hospital volume.^25^ Additional studies are required to determine their effects on hospital mortality globally.

In conclusion, the number of patients undergoing surgery in hospitals with reduced resources and weak processes of care is higher in low-income and middle-income settings, putting these patients at additional risk. Although early mortality after cancer surgery is known to be increased in LMICs, the improvement of facilities, processes, and quality of care can dramatically reduce perioperative mortality in these settings. A more comprehensive study of systems strengthening and improvement interventions to reduce postoperative mortality would provide important information on mechanisms to improve cancer surgery outcomes for the large numbers of patients who receive care at these institutions.

## Data sharing

The dataset can be explored using an online visualisation application (https://cancer.globalsurg.org). Hospital-level data can be shared by application to the corresponding author. For analyses of patient-level identifiable data within our trusted research environment, please contact the corresponding author.

## Declaration of interests

MIvBH reports personal fees from Mylan, Alesi Surgical, Johnson and Johnson, and Medtronic; grants and non-financial support from Stryker; and grants from Olympus, outside the submitted work; all fees were paid to their institution. PB reports grants and personal fees from the Medical Research Council; grants from National Institute for Health Research (NIHR) Health Technology Assessment and the Wellcome Trust; and personal fees from AG Biotest, outside the submitted work. TPK reports personal fees from Olympus Surgical, outside the submitted work. All other authors declare no competing interests.
