# Supplementary material for: Effects of hospital facilities on patient outcomes after cancer surgery: an international, prospective, observational study
Source: Lancet Glob Health. 2022 May 24;10(7):e1003–11. doi: 10.1016/S2214-109X(22)00168-1 (PMC9210173; doi:10.1016/S2214-109X(22)00168-1)
Supplement: Supplementary appendix [file mmc1.pdf]

THE LANCET
Global Health

Supplementary appendix

This appendix formed part of the original submission and has been peer reviewed. We post it as supplied by the authors.

Supplement to: GlobalSurg Collaborative and NIHR Global Health Research Unit on Global Surgery. Effects of hospital facilities on patient outcomes after cancer surgery: an international, prospective, observational study. *Lancet Glob Health* 2022; published online May 24. https://doi.org/10.1016/S2214-109X(22)00168-1.

Supplementary material

Table of Contents

Appendix 1. Hospital-level survey and definitions 1
Appendix 2. Proportion of hospitals performing elective operations for each cancer by income group 6
Appendix 3. Proportion of hospitals performing elective operations for each cancer stratified by included cancers in GlobalSurg 3 6
Appendix 4. Relationship between hospital facilities and country income group 7
Appendix 5. Distribution of outcomes across number of available hospital facilities 8
Appendix 6A. Case volume stratified by hospital inclusion 9
Appendix 6B. Adjusted mortality rates stratified by hospital inclusion 10
Appendix 7. Hospital facility selection using backward elimination and AIC 11
Appendix 8. Hospital facility selection sensitivity analysis 12
Appendix 9. Patient characteristics by hospital facility level 13
Appendix 10. Relationship between hospital facility level and postoperative complication rates 15
Appendix 11A. Relationship between hospital facility level and patient safety / quality of cancer care metrics 16
Appendix 11B. Relationship between hospital facility level and patient safety / quality of cancer care metrics 17
Appendix 12. Proportion of hospitals performing elective operations for each cancer across hospital facility level 18
Appendix 13. Adjusted mortality rate across hospital facility level 19
Appendix 14. Adjusted mortality rate for colorectal and gastric cancer across hospital facility level 20
Appendix 15. Adjusted major complication rates across hospital facility level 21
Appendix 16. Sensitivity analysis using imputed dataset 22
Appendix 17. Sensitivity analysis - adjusted outcome rates across all eleven hospital facilities 24
Appendix 18. Adjusted major complication rates across hospital facility level 24
Appendix 19. Capacity to rescue patients following major complication following case-mix adjustment 25
Appendix 20. Absolute risk for 30-day mortality associated with four or more hospital facilities within each income group stratified by cancer type and sex 26
Appendix 21. Absolute risk for 30-day mortality in hospitals with more than three facilities within each income group stratified by cancer type and sex 27
Appendix 22. Absolute risk for 30-day mortality in hospitals with more than three facilities within each income group stratified by cancer type and sex 28
Appendix 19. Absolute risk for 30-day mortality associated with four or more hospital facilities within each income group stratified by cancer type and sex for higher risk surgical patients (ASA grade ≥3) 30
Appendix 20. Absolute risk for 30-day mortality associated with four or more hospital facilities within each income group stratified by cancer type and sex for higher risk surgical patients (ASA grade ≥3) 31
Authorship 32
Data Validators 44

Appendix 1. Hospital-level survey and definitions

Hospital characteristics

1. Please select your hospital (dropdown list)

2. What is the approximate size of population for which your hospital provides cancer care for?

   <50,000 | 50,000-199,999 | 200,000-499,999 | 500,000-999,999 | 1,000,000-1,999,999 | 2,000,000+
   How certain are you of this figure: Total guess | Uncertain | Reasonably sure | Certain

3. What type of hospital is your centre? *Please select the most appropriate description*
   - Non-referral hospital
     Receive referrals only from community clinics and general practitioners
   - Referral hospital
     Receive referrals from other trained surgeons, as well as from community clinics and general practitioners
   - Specialist cancer hospital
     A hospital which solely treats patients with cancer

Diagnosis

For the following questions please answer for the last six months for patients undergoing surgery for cancer

4. If I want to request an ultrasound for my patient, either free or by payment, it is usually:
   - Not available at all
   - On site and always available
   - On site but not working / available all the time
   - Available at another hospital (patients are transferred)
   - No

5. If I want to request a computed tomography scan during standard working hours (0800 to 1700), either free or by payment, it is usually:
   - Not available at all
   - On site and always available
   - On site but not working / available all the time
   - Available at another hospital (patients are transferred)

6. If I want to request pathology services, either free or by payment, for cancer biopsies or resected specimens it is usually: (please s*elect the most commonly encountered situation)*
   - Not available
   - On site and always available
   - On site but only available intermittently
   - Available at another hospital
   - Available in another country

7. Branching logic: If pathology for a cancer specimen is requested on a routine (non-urgent) basis, on average, how long on average does it take for this result to be available (in weeks)?

8. Branching logic: In the last six months, what proportion of patients received a pathology result following their cancer resection (for all cancers treated at your hospital)?
   Slider: 0 – 100
   How certain are you of this figure: Total guess | Uncertain | Reasonably sure | Certain

Patient journey

9. Do you have a Multidisciplinary Team (MDT) meeting to discuss treatment options for patients with breast, gastric and/or colorectal cancer at your site or another hospital?

   Definition of MDT: Meeting of a group of professionals from two or more clinical disciplines who together make decisions regarding cancer treatment or care of individual patients[1]

   - No
   - Yes – only for some cancers at my hospital
   - Yes – for all cancers (breast, colorectal & gastric)

10. Branching logic: For the last 10 patients with breast, gastric and/or colorectal cancer who received elective (non-emergency) surgery, how many were discussed in the multi-disciplinary team (MDT) meeting?
    None | 1-2 | 3-4 | 5-7 | 8-9 | All 10 patients
    These were: Breast cancer only | GI cancer only | Breast and GI cancer (if only some cancers)

11. Branching logic: Please select the appropriate response for each professional
    Not available | Available in hospital but attends <75% MDTs | Available in hospital and attends >75% MDTs

    - Oncologist (including radiotherapy / chemotherapy specialists or both)
    - Radiologist
    - Pathologist
    - Specialist cancer nurse / clinical nurse specialist
    - Palliative care specialist
    - Surgeon

    Oncologist definition: Healthcare professional trained in and provides medical treatment for cancer (radiotherapy, chemotherapy, hormone therapy)[2]

    Specialist cancer nurse definition: A registered nurse with additional education/training, skill and specialisation in cancer care[3,4]

    Palliative care specialist definition: a person who organises care and aims to improve quality of life of patients and their families facing the problem of a life-threatening illness[5]

12. Branching logic (if no MDT is present): Is there an oncologist at your hospital? *(select the most appropriate response for your hospital)*

    Oncologist definition: Healthcare professional (or surgeon) trained in and provides medical treatment for cancer (radiotherapy, chemotherapy, hormone therapy)[1]

    - No
    - Yes – a clinician who provides cancer care as well as care of non-cancer conditions
    - Yes – a clinician who only provides cancer care

13. Branching logic (if no MDT is present): Do you have a trained doctor or healthcare professional in palliative medicine?

    Defined as a Person who organises care and aims to improve quality of life of patients and their families facing the problem of a life-threatening illness.[3] *This includes*

    - No
    - Yes

Oncology treatment

For the following questions please answer for the last six months for cancers surgically treated at your hospital (unless otherwise specified)

14. Is radiotherapy usually available for your patients (either free or by payment)?
    - No
    - Yes - at the same hospital
    - Yes - at another hospital (within 10 km)
    - Yes - at another hospital (10 - 50 km)
    - Yes - at another hospital (> 50 km away)
    - Yes - in another country

Distance measured using road network and shortest distance (not straight line distance)

15. Branching logic: What type of radiotherapy machine is available?
    - Cobalt accelerator
    - Linear accelerator

16. Are chemotherapy drugs usually available for your patients (either free or by payment)?
    - No
    - Yes - at the same hospital
    - Yes - at another hospital (within 10 km)
    - Yes – at another hospital (10 - 50 km)
    - Yes – at another hospital (>50 km away)
    - Yes - in another country

17. Do patients at your hospital make out of pocket (cash) payments for their surgical care (i.e. their surgery, postoperative care, medications, consumables)? (*Please select the most appropriate)*
    - No patients make out of pocket payments
    - Yes - patients who do not have insurance make out of pocket payments for at least some part of their care

- Yes - those patients that are able to afford to do so are asked to make out of pocket payments for at least some part of their care
- Yes - all patients make out of pocket payments for at least some part of their care

18. Do you have a designated area for post-operative care at your hospital?

    Post-operative care area: Defined as continuous patient monitoring of vital signs, including the use of a pulse oximeter, immediately after surgery by a designated carer for immediate intervention if required)[6]

    - No
    - Yes - sometimes
    - Yes - all of the time

19. What is the highest level of bed available for patients receiving cancer surgery at your hospital (if required)?
    - Post-operative ward bed only
    - Specialist ward(s) providing intensive treatment and monitoring (HDU / ITU)

20. Do your patients have access to opiate pain medication within the first 24 hours of admission and following surgery?

    Opiate analgesia defined as Pethidine, Fentanyl or Morphine[4]

    - No
    - Yes – but not always available
    - Yes – available all of the time

Surgical management

21. For the following operations for cancer, have you performed any of these in the past three months?

    For each cancer: No | This cancer is referred to another hospital routinely | Yes, as an emergency only | Yes, as a planned elective procedure (with or without emergency procedures)

    - Breast: wide local excision / mastectomy
    - Oesophageal cancer (including oesophogastric junctional tumours): oesophagectomy
    - Lung: lobectomy / pneumonectomy / laryngectomy
    - Stomach (including GISTs): total/partial gastrectomy / gastrojejunostomy
    - Colon (excluding rectum): colonic resection
    - Rectum: anterior resection / abdomino-peroneal excision
    - Liver: hepatectomy
    - Pancreas: pancreatectomy (all types)
    - Kidney: nephrectomy
    - Bladder cancer: cystectomy
    - Prostate: prostatectomy
    - Cervical and uterine cancer: hysterectomy / trachelectomy
    - Ovarian: oophrectomy
    - Thyroid: hemi-thyroidectomy / total thyroidectomy
    - Lip and oral cavity: wide local excision / Mohs micrographic surgery / glossectomy
    - Malignant melanoma: surgical excision

References

1. Multidisciplinary Team Meeting [Internet]. [cited 2022 May 01]. Available from: https://www.datadictionary.nhs.uk/data_dictionary/nhs_business_definitions/m/multidisciplinary_team_meeting_de.asp?showнav=1
2. Types of Oncologists | American Society of Clinical Oncology (ASCO) [Internet]. [cited 2022 May 01]. Available from: https://www.cancer.net/navigating-cancer-care/cancer-basics/cancer-care-team/types-oncologists
3. National Cancer Action Team | Excellence in Cancer Care: The contribution of the Clinical Nurse Specialist [Internet]. Macmillan. [cited 2022 May 01]. Available from: https://www.macmillan.org.uk/documents/aboutus/commissioners/excellenceincancercarethecontributionoftheclinicalnursespecialist.pdf
4. Clinical Nurse Specialist [Internet]. [cited 2022 May 01]. Available from: https://datadictionary.nhs.uk/nhs_business_definitions/clinical_nurse_specialist.html?hl=clinical%2Cnurse%2Cspecialist
5. WHO | WHO Definition of Palliative Care [Internet]. WHO. [cited 2022 May 01]. Available from: https://www.who.int/cancer/palliative/definition/en/
6. WHO | WHO Postoperative note and orders [Internet]. WHO [cited 2022 May 01]. Available from: https://www.who.int/surgery/publications/Postoperativecare.pdf

Appendix 2. Proportion of hospitals performing elective operations for each cancer by income group

Operation	High (n = 91)	Upper middle (n = 57)	Low/lower middle (n = 90)	p
Breast	75 (82.4)	50 (87.7)	84 (93.3)	0.080
Oesophagus	44 (48.4)	34 (59.6)	46 (51.1)	0.397
Lung	42 (46.2)	30 (52.6)	38 (42.2)	0.467
Gastric	76 (83.5)	53 (93.0)	73 (81.1)	0.132
Liver	50 (54.9)	42 (73.7)	35 (38.9)	<0.001
Pancreas	52 (57.1)	44 (77.2)	42 (46.7)	0.001
Renal	72 (79.1)	44 (77.2)	66 (73.3)	0.649
Colorectal	87 (95.6)	56 (98.2)	85 (94.4)	0.531
Rectum	83 (91.2)	53 (93.0)	73 (81.1)	0.045
Cervical	72 (79.1)	44 (77.2)	64 (71.1)	0.433
Ovarian	71 (78.0)	49 (86.0)	65 (72.2)	0.148

Appendix 3. Proportion of hospitals performing elective operations for each cancer stratified by included cancers in GlobalSurg 3

	All hospitals				LMIC hospitals			
Operation	Breast only (n = 29)	Colorectal and gastric (n = 21)	All cancers (n = 180)	p	Breast only (n = 17)	Colorectal and gastric (n = 8)	All cancers (n = 117)	p
Oesophagus	0 (0.0)	8 (38.1)	115 (63.9)	<0.001	0 (0.0)	2 (25.0)	77 (65.8)	<0.001
Lung	1 (3.4)	6 (28.6)	102 (56.7)	<0.001	0 (0.0)	3 (37.5)	65 (55.6)	<0.001
Liver	1 (3.4)	11 (52.4)	114 (63.3)	<0.001	1 (5.9)	5 (62.5)	70 (59.8)	<0.001
Pancreas	1 (3.4)	12 (57.1)	125 (69.4)	<0.001	1 (5.9)	6 (75.0)	79 (67.5)	<0.001
Renal	14 (48.3)	17 (81.0)	151 (83.9)	<0.001	10 (58.8)	7 (87.5)	93 (79.5)	0.127
Rectum	18 (62.1)	19 (90.5)	170 (94.4)	<0.001	9 (52.9)	7 (87.5)	108 (92.3)	<0.001
Cervical	18 (62.1)	12 (57.1)	149 (82.8)	0.003	13 (76.5)	5 (62.5)	89 (76.1)	0.685
Ovarian	19 (65.5)	12 (57.1)	153 (85.0)	0.001	13 (76.5)	5 (62.5)	95 (81.2)	0.422

Appendix 4. Relationship between hospital facilities and country income group

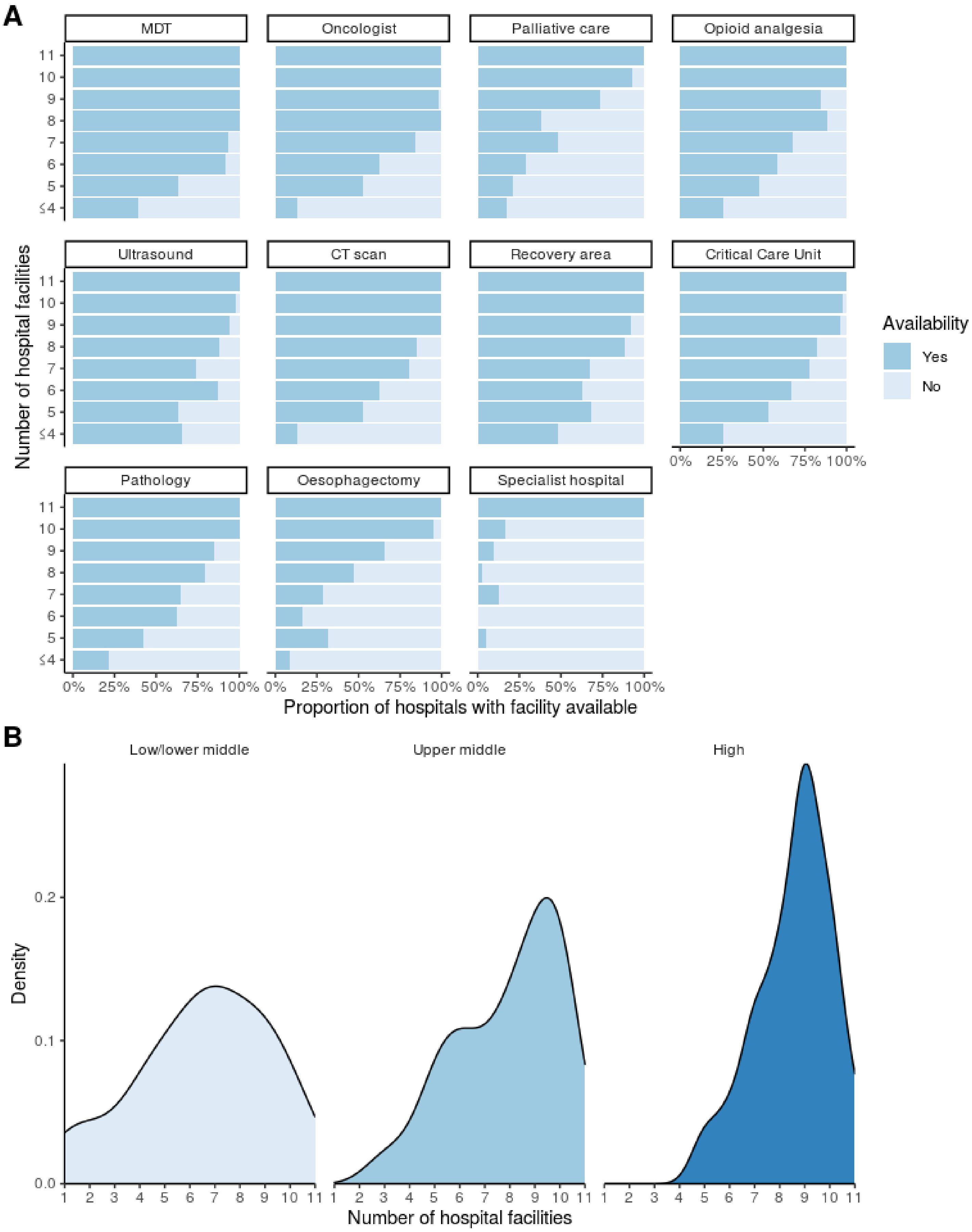

Appendix 5. Distribution of outcomes across number of available hospital facilities

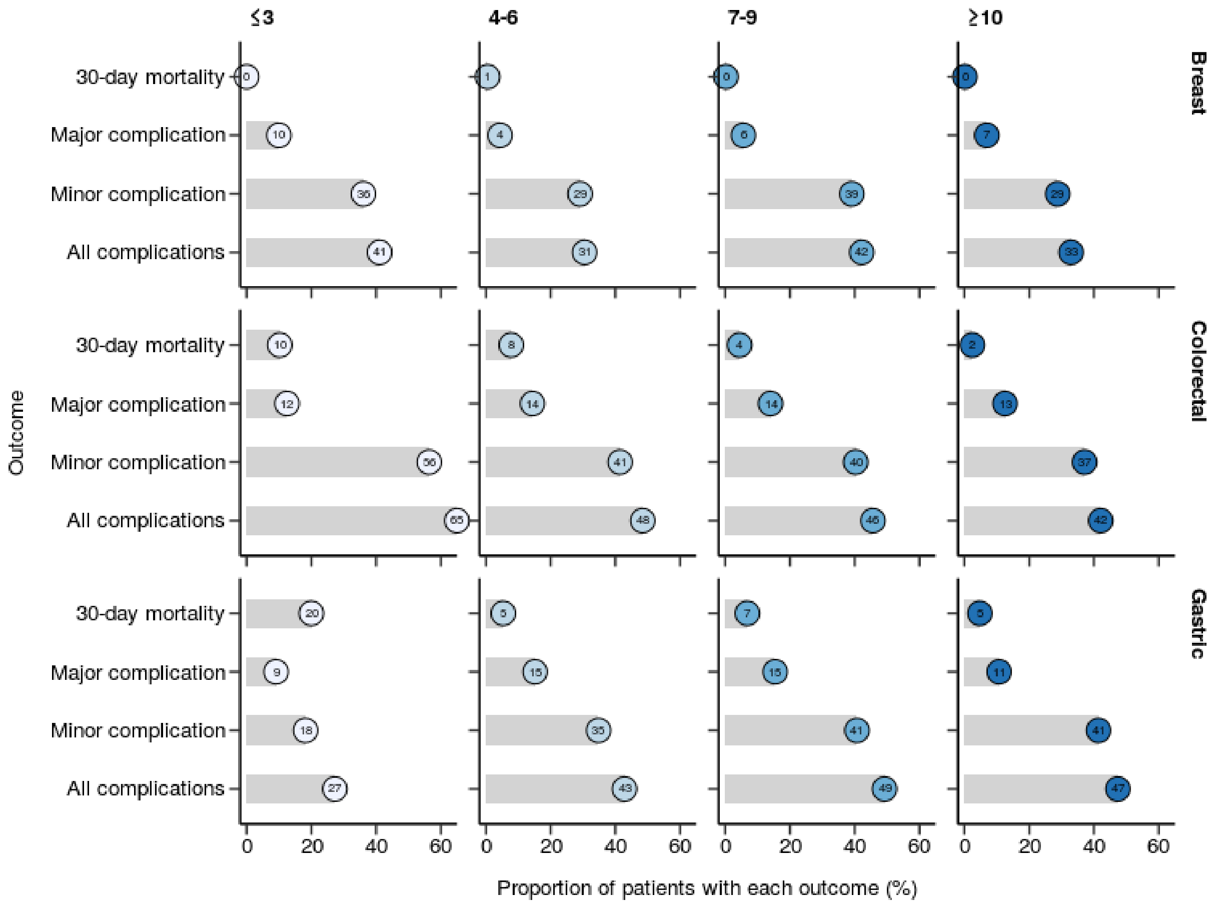

Minor complication - Clavien-Dindo grade I or II; Major complication - Clavien-Dindo grade III or IV; All complications – Clavien-Dindo grade I-V

Appendix 6A. Case volume stratified by hospital inclusion

Case volumes were calculated as the median number of patients recruited across each hospital during a 28-day data collection period

		Hospital-level data available		
Cancer type	WB tertile	Yes	No	P value
Breast	High	9.0 (4.0 to 19.8)	10.0 (5.0 to 18.0)	0.655
	Upper middle	5.0 (2.0 to 8.5)	2.0 (1.5 to 5.5)	0.182
	Low/lower middle	3.0 (1.0 to 6.0)	2.0 (2.0 to 3.0)	0.358
Colorectal	High	6.0 (4.0 to 11.0)	8.0 (5.0 to 12.0)	0.095
	Upper middle	4.0 (2.0 to 7.0)	2.0 (1.0 to 3.5)	0.052
	Low/lower middle	2.0 (1.0 to 4.8)	2.0 (1.0 to 4.0)	0.524
Gastric	High	2.0 (1.0 to 3.0)	2.0 (1.0 to 3.0)	0.884
	Upper middle	2.0 (1.0 to 3.0)	1.5 (1.0 to 3.5)	0.755
	Low/lower middle	1.0 (1.0 to 2.0)	1.0 (1.0 to 2.0)	0.980

Appendix 6B. Adjusted mortality rates stratified by hospital inclusion

Adjusted mortality rates were calculated using generalised estimating equations (GEE) to account for potential confounders (WB tertile, age, sex, cancer type, ECOG performance status, ASA grade, disease stage, and operative urgency). Confidence intervals (CIs) and a P value for trend were fitted using the multilevel logistic regression model with all confounders as covariates

WB tertile	Hospital-level data available	Hospital number	Patient number	Adjusted mortality (95% CI)	Odds ratio	P value
High	Yes	90	3202	1.4 (1.3 to 1.5)	Ref	
	No	150	5038	1.4 (1.3 to 1.5)	0.98 (0.67 to 1.42)	0.92
Upper middle	Yes	57	2011	2.2 (2 to 2.4)	Ref	
	No	24	563	2.2 (1.8 to 2.6)	0.97 (0.51 to 1.86)	1.00
Low/lower middle	Yes	91	3655	2.5 (2.3 to 2.7)	Ref	
	No	16	186	4.3 (3.3 to 5.4)	1.76 (0.84 to 3.68)	0.15

Appendix 7. Hospital facility selection using backward elimination and AIC

Hospital facility	Coefficient	Standard error	Z value	P value	Final model
Intercept	-1.79387	0.37231	-4.818	<0.001	-
MDT	0.16836	0.44184	0.381	0.7032	-
Oncologist	-0.30738	0.13607	-2.259	0.0239	Included
Palliative care	0.06800	0.17714	0.384	0.7011	-
Opioid analgesia	-0.53143	0.20977	-2.533	0.0113	Included
Ultrasound	-0.49329	0.24598	-2.005	0.0449	Included
CT scan	-0.70332	0.23303	-3.018	0.0025	Included
Recovery area	-0.30676	0.20158	-1.522	0.1281	-
Critical Care unit	-0.30738	0.13607	-2.259	0.0239	Included
Pathology	0.01728	0.25915	0.067	0.9468	-
Hospital type	-0.57405	0.30024	-1.912	0.0559	-
Oesophagectomy	-0.11290	0.16960	-0.666	0.5056	-

Appendix 8. Hospital facility selection sensitivity analysis

Performed using bootstrap procedure (n=5000)

Hospital facility	Selected (%)	Deviance	Residual degrees of freedom	Residual deviation	AIC	Final model
CT scan	91.65	-	-	-	-	Included
Opioid analgesia	84.80	-	-	-	-	Included
Ultrasound	83.35	-	-	-	-	Included
Oncologist	71.80	-	-	-	-	Included
Critical Care unit	69.90	-	-	-	-	Included
Hospital type	38.35	0.001	9555	1843.525	1869.525	-
Recovery area	33.90	0.004	9556	1843.53	1867.530	-
Oesophagectomy	29.70	0.551	9560	1844.437	1860.437	-
Palliative care	26.60	0.145	9558	1843.679	1863.679	-
MDT	24.30	0.207	9559	1843.886	1861.886	-
Pathology	22.65	0.004	9557	1843.534	1865.534	-

Appendix 9. Patient characteristics by hospital facility level

Numbers are n (%) or mean (SD). High income included 20 countries and 91 hospitals. Upper-middle income included 19 countries and 57 hospitals. Lower-middle income or low income included 27 countries and 90 hospitals. The total column therefore includes 66 countries and 238 hospitals. ASA=American Society of Anesthesiologists. ECOG=Eastern Cooperative Oncology Group.

Hospital facility level		5 (n = 6378)	4 (n = 2013)	≤3 (n = 1294)	Total (n = 9685)	p
Distribution of patients across WB income (tertile)						
	High	2669 (41.8)	867 (43.1)	100 (7.7)	3636 (37.5)	<0.001
	Upper middle	1375 (21.6)	251 (12.5)	493 (38.1)	2119 (21.9)	
	Low/lower middle	2334 (36.6)	895 (44.5)	701 (54.2)	3930 (40.6)	
Cancer type						
	Breast	3834 (60.1)	1192 (59.2)	625 (48.3)	5651 (58.3)	<0.001
	Colorectal (colon or rectum)	2010 (31.5)	654 (32.5)	534 (41.3)	3198 (33.0)	
	Gastric (stomach)	534 (8.4)	167 (8.3)	135 (10.4)	836 (8.6)	
Age (years)		57.9 (14.4)	58.5 (14.2)	56.2 (13.8)	57.8 (14.3)	<0.001
Sex (%)						
	Male	1489 (23.3)	465 (23.1)	379 (29.3)	2333 (24.1)	<0.001
	Female	4886 (76.6)	1546 (76.8)	914 (70.6)	7346 (75.8)	
	(Missing)	3 (0.0)	2 (0.1)	1 (0.1)	6 (0.1)	
ECOG performance status						
	0	3668 (57.5)	1007 (50.0)	612 (47.3)	5287 (54.6)	<0.001
	1	1520 (23.8)	519 (25.8)	356 (27.5)	2395 (24.7)	
	2	750 (11.8)	226 (11.2)	157 (12.1)	1133 (11.7)	
	3/4	198 (3.1)	94 (4.7)	115 (8.9)	407 (4.2)	
	(Missing)	242 (3.8)	167 (8.3)	54 (4.2)	463 (4.8)	
ASA						
	I	1385 (21.7)	387 (19.2)	382 (29.5)	2154 (22.2)	<0.001
	II	3469 (54.4)	1099 (54.6)	668 (51.6)	5236 (54.1)	
	III	1191 (18.7)	359 (17.8)	148 (11.4)	1698 (17.5)	
	IV	89 (1.4)	32 (1.6)	22 (1.7)	143 (1.5)	
	V	6 (0.1)	11 (0.5)	3 (0.2)	20 (0.2)	
	(Missing)	238 (3.7)	125 (6.2)	71 (5.5)	434 (4.5)	
Stage						
	0	192 (3.0)	51 (2.5)	11 (0.9)	254 (2.6)	<0.001
	I	2631 (41.3)	812 (40.3)	334 (25.8)	3777 (39.0)	
	II	593 (9.3)	216 (10.7)	146 (11.3)	955 (9.9)	
	III	2456 (38.5)	777 (38.6)	608 (47.0)	3841 (39.7)	
	IV	453 (7.1)	146 (7.3)	179 (13.8)	778 (8.0)	
	(Missing)	53 (0.8)	11 (0.5)	16 (1.2)	80 (0.8)	
Urgency						
	Elective	6081 (95.3)	1850 (91.9)	1180 (91.2)	9111 (94.1)	<0.001

	Emergency	295 (4.6)	161 (8.0)	114 (8.8)	570 (5.9)	
	(Missing)	2 (0.0)	2 (0.1)	0 (0.0)	4 (0.0)	
30-day mortality						
	Alive	6210 (97.4)	1947 (96.7)	1216 (94.0)	9373 (96.8)	<0.001
	Dead	97 (1.5)	42 (2.1)	56 (4.3)	195 (2.0)	
	(Missing)	71 (1.1)	24 (1.2)	22 (1.7)	117 (1.2)	
Major complication						
	Yes	603 (9.5)	192 (9.5)	143 (11.1)	938 (9.7)	0.178
	No	5744 (90.1)	1800 (89.4)	1136 (87.8)	8680 (89.6)	
	(Missing)	31 (0.5)	21 (1.0)	15 (1.2)	67 (0.7)	

Appendix 10. Relationship between hospital facility level and postoperative complication rates

	Hospital facility level		5<br>(n = 3834)	4<br>(n = 1192)	≤3<br>(n = 625)	p
Breast	Surgical site infection	No	387 (10.1)	125 (10.5)	100 (16.0)	<0.001
	Postoperative haemorrhage	Yes	118 (3.1)	40 (3.4)	20 (3.2)	0.864
	Seroma	No	738 (19.2)	231 (19.4)	147 (23.5)	0.030

	Hospital facility count	5<br>(n = 2544)	4<br>(n = 821)	≤3<br>(n = 669)	p
Colorectal and gastric	Surgical site infection	384 (15.1)	115 (14.0)	189 (28.3)	<0.001
	Intra-abdominal abscess	125 (4.9)	44 (5.4)	34 (5.1)	0.835
	Anastomotic leak	125 (4.9)	49 (6.0)	40 (6.0)	0.303
	Postoperative haemorrhage	105 (4.1)	39 (4.8)	28 (4.2)	0.730

Appendix 11A. Relationship between hospital facility level and patient safety / quality of cancer care metrics

Hospital facility level		>3 (n = 8391)	≤3 (n = 1294)	p
Surgical safety checklist used		6872 (83.7)	910 (73.6)	<0.001
Anastomosis performed		2553 (77.5)	467 (72.9)	0.013
Negative margin		7015 (90.8)	948 (87.5)	0.001
Length of stay (days)		3.0 (1.0 to 7.0)	5.0 (3.0 to 9.0)	<0.001
Readmission		397 (4.8)	68 (5.4)	0.404
Method of follow-up at 30 days	Still inpatient or readmitted	250 (3.0)	79 (6.2)	<0.001
	Clinic review	6273 (75.9)	584 (45.6)	
	Telephone review	1414 (17.1)	578 (45.2)	
	Community/home review	27 (0.3)	1 (0.1)	
	Discharged before 30 days and not contacted again	298 (3.6)	38 (3.0)	
Radiotherapy available		6620 (78.9)	588 (45.4)	<0.001
Chemotherapy available		7808 (93.1)	958 (74.0)	<0.001
Multidisciplinary tumour board available for all cancers treated in hospital		6568 (78.3)	405 (31.3)	<0.001

Appendix 11B. Relationship between hospital facility level and patient safety / quality of cancer care metrics

		5 (n = 6378)	4 (n = 2013)	≤3 (n = 1294)	p
Surgical safety checklist used		5160 (83.1)	1712 (85.4)	910 (73.6)	<0.001
Anastomosis performed		1949 (77.9)	604 (76.2)	467 (72.9)	0.024
Negative margin		5403 (90.9)	1612 (90.7)	948 (87.5)	0.002
Length of stay (days)		3.0 (1.0 to 7.0)	3.0 (1.0 to 7.0)	5.0 (3.0 to 9.0)	<0.001
Readmission		309 (4.9)	88 (4.5)	68 (5.4)	0.490
Method of follow-up at 30 days	Still inpatient or readmitted	168 (2.7)	82 (4.1)	79 (6.2)	<0.001
	Clinic review	4978 (79.4)	1295 (64.9)	584 (45.6)	
	Telephone review	882 (14.1)	532 (26.7)	578 (45.2)	
	Community/home review	21 (0.3)	6 (0.3)	1 (0.1)	
	Discharged before 30 days and not contacted again	219 (3.5)	79 (4.0)	38 (3.0)	
Radiotherapy available		5280 (82.8)	1340 (66.6)	588 (45.4)	<0.001
Chemotherapy available		6091 (95.5)	1717 (85.3)	958 (74.0)	<0.001
Multidisciplinary tumour board available for all cancers treated in hospital		5269 (82.6)	1299 (64.5)	405 (31.3)	<0.001

Numbers are n (%) or median (IQR). Negative resection margins were defined according to National Institute for Clinical Excellence Guidance and guidelines. MDT – multidisciplinary team

Appendix 12. Proportion of hospitals performing elective operations for each cancer across hospital facility level

Operation	5 (n = 113)	4 (n = 63)	≤3 (n = 62)	p
Breast	101 (89.4)	53 (84.1)	55 (88.7)	0.575
Oesophagus	76 (67.3)	28 (44.4)	20 (32.3)	<0.001
Lung	73 (64.6)	24 (38.1)	13 (21.0)	<0.001
Gastric	104 (92.0)	52 (82.5)	46 (74.2)	0.006
Liver	80 (70.8)	30 (47.6)	17 (27.4)	<0.001
Pancreas	86 (76.1)	30 (47.6)	22 (35.5)	<0.001
Renal	98 (86.7)	43 (68.3)	41 (66.1)	0.002
Colorectal	111 (98.2)	59 (93.7)	58 (93.5)	0.206
Rectum	108 (95.6)	52 (82.5)	49 (79.0)	0.002
Cervical	93 (82.3)	44 (69.8)	43 (69.4)	0.074
Ovarian	97 (85.8)	44 (69.8)	44 (71.0)	0.017

Appendix 13. Adjusted mortality rate across hospital facility level

Adjusted mortality rates were calculated using generalised estimating equations (GEE) to account for clustering of patients in hospital and for potential confounders (WB tertile, age, sex, cancer type, ECOG performance status, ASA grade, disease stage, and operative urgency). Confidence intervals (CIs) and a P value for trend were fitted using the multilevel logistic regression model with the number of hospital facilities and all confounders as covariates

	Hospital facility level	Hospital number (%)	Number of Patients (%)	Adjusted mortality (95% CI)	Odds ratio	P value
All cancers	5	113 (47.7)	5912 (66.6)	1 (0.7 to 1.2)	Ref	
	4	63 (26.6)	1787 (20.2)	1.5 (0.9 to 2)	1.49 (0.94 to 2.37)	0.092
	≤3	61 (25.7)	1169 (13.2)	3.7 (2.6 to 4.8)	3.85 (2.58 to 5.75)	<0.001
Colorectal and gastric cancer	5	105 (48.0)	2388 (63.8)	4.1 (3.8 to 4.3)	Ref	
	4	57 (26.0)	753 (20.1)	5.2 (4.6 to 5.8)	1.29 (0.88 to 1.89)	0.22
	≤3	57 (26.0)	602 (16.1)	6.9 (6 to 7.8)	1.73 (1.18 to 2.52)	0.006

Appendix 14. Adjusted mortality rate for colorectal and gastric cancer across hospital facility level

	Hospital facility level	Patient n (%)	Adjusted mortality (95% CI)
Colorectal	5	1877 (63.3)	3.5 (3.3 to 3.8)
	4	610 (20.6)	3.9 (3.4 to 4.5)
	≤3	478 (16.1)	8.8 (7.6 to 9.9)
Gastric	5	511 (65.7)	4.9 (4.3 to 5.5)
	4	143 (18.4)	4.2 (3.1 to 5.3)
	≤3	124 (15.9)	11.3 (8.8 to 13.8)

Adjusted mortality rates were calculated using generalised estimating equations (GEE) to account for clustering of patients in hospital and for potential confounders (WB tertile, age, sex, cancer type, ECOG performance status, ASA grade, disease stage, and operative urgency). Confidence intervals (CIs) fitted using the multilevel logistic regression model with the number of hospital facilities and all confounders as covariates

Appendix 15. Adjusted major complication rates across hospital facility level

Adjusted major complication rates were calculated using generalised estimating equations (GEE) to account for clustering of patients in hospital and for potential confounders (WB tertile, age, sex, cancer type, ECOG performance status, ASA grade, disease stage, and operative urgency). Confidence intervals (CIs) and a P value for trend were fitted using the multilevel logistic regression model with the number of hospital facilities and all confounders as covariates

	Hospital facility level	Hospital number (%)	Number of Patients (%)	Adjusted major complication rate (95% CI)	Odds ratio	P value
All cancers	5	113 (47.5)	5951 (66.7)	9.3 (9.1 to 9.5)	Ref	
	4	63 (26.5)	1789 (20.1)	9.6 (9.2 to 9.9)	1.03 (0.86 to 1.23)	0.746
	≤3	62 (26.0)	1175 (13.2)	11.8 (11.2 to 12.3)	1.30 (1.06 to 1.58)	0.011
Colorectal and gastric cancer	5	105 (47.7)	2405 (63.8)	13.5 (13.2 to 13.8)	Ref	
	4	57 (26.0)	755 (20.1)	15.9 (15.2 to 16.6)	1.21 (0.97 to 1.52)	0.105
	≤3	58 (26.3)	608 (16.1)	18.0 (17.1 to 18.8)	1.40 (1.11 to 1.78)	0.007

Appendix 16. Sensitivity analysis using imputed dataset

Adjusted mortality rate

Adjusted mortality rates were calculated using generalised estimating equations (GEE) to account for clustering of patients in hospital and for potential confounders (WB tertile, age, sex, cancer type, ECOG performance status, ASA grade, disease stage, and operative urgency). Confidence intervals (CIs) and a P value for trend were fitted using the multilevel logistic regression model with the number of hospital facilities and all confounders as covariates

	Hospital facility level	Hospital number	Patient number	Adjusted mortality rate (95% CI)	Odds ratio	P value
All cancers	5	215	9617	1.5 (1.4 to 1.6)	Ref	
	4	140	3736	1.9 (1.8 to 2.1)	1.29 (0.97 to 1.72)	0.079
	≤3	72	1302	4.8 (4.2 to 5.3)	3.29 (2.43 to 4.46)	<0.001
Colorectal and gastric cancer	5	205	4407	3.0 (2.9 to 3.2)	Ref	
	4	127	1932	3.5 (3.2 to 3.8)	1.15 (0.86 to 1.56)	0.350
	≤3	66	670	9.1 (8 to 10.1)	3.22 (2.35 to 4.41)	<0.001

Major complication rates

Adjusted major complication rates were calculated using generalised estimating equations (GEE) to account for clustering of patients in hospital and for potential confounders (WB tertile, age, sex, cancer type, ECOG performance status, ASA grade, disease stage, and operative urgency). Confidence intervals (CIs) and a P value for trend were fitted using the multilevel logistic regression model with the number of hospital facilities and all confounders as covariates

	Hospital facility level	Hospital number	Patient number	Adjusted major complication rate (95% CI)	Odds ratio	P value
All cancers	5	215	9669	9.7 (9.6 to 9.8)	Ref	
	4	140	3742	10.0 (9.8 to 10.3)	1.04 (0.91 to 1.18)	0.560
	≤3	73	1311	11.5 (11.1 to 12.0)	1.21 (1.01 to 1.46)	0.043
Colorectal and gastric cancer	5	205	4434	13.7 (13.5 to 13.9)	Ref	
	4	127	1938	14.9 (14.5 to 15.3)	1.11 (0.95 to 1.29)	0.196
	≤3	67	676	17.8 (17 to 18.6)	1.38 (1.11 to 1.71)	0.005

Adjusted capacity to rescue rates

Adjusted mortality rates after major complication were calculated using generalised estimating equations (GEE) to account for clustering of patients in hospital and for potential confounders (WB tertile, age, sex, cancer type, ECOG performance status, ASA grade, disease stage, and operative urgency). Confidence intervals (CIs) and a P value for trend were fitted using the multilevel logistic regression model with the number of hospital facilities and all confounders as covariates

	Hospital facility level	Hospital number	Patient number	Adjusted capacity to rescue (95% CI)	Odds ratio	P value
All cancers	5	175	940	84.7 (83.6 to 85.9)	Ref	
	4	97	377	81.0 (78.9 to 83.0)	0.77 (0.56 to 1.05)	0.100
	≤3	49	148	57.9 (53.3 to 62.5)	0.25 (0.17 to 0.36)	<0.001
Colorectal and gastric cancer	5	156	599	78.0 (76.5 to 79.5)	Ref	
	4	93	289	76.9 (74.6 to 79.2)	0.94 (0.67 to 1.31)	0.730
	≤3	40	122	50.0 (45.5 to 54.5)	0.28 (0.19 to 0.42)	<0.001

Appendix 17. Sensitivity analysis - adjusted outcome rates across all eleven hospital facilities

All eleven hospital facilities were included within a sensitivity analysis, with hospitals categorised into different facility levels by patient distribution. Adjusted mortality rates were calculated using generalised estimating equations (GEE) to account for clustering of patients in hospital and for potential confounders (WB tertile, age, sex, cancer type, ECOG performance status, ASA grade, disease stage, and operative urgency). Confidence intervals (CIs) and a P value for trend were fitted using the multilevel logistic regression model with the number of hospital facilities and all confounders as covariates

	Hospital facility level	Hospital number	Patient number	Adjusted mortality (95% CI)	Odds ratio	P value
All cancers	10-11	54	4009	0.9 (0.6 to 1.2)	Ref	
	8-9	87	2665	1.2 (0.8 to 1.6)	1.3 (0.8 to 2.1)	0.320
	≤7	96	2194	2.7 (2.1 to 3.4)	3.1 (2.05 to 4.71)	<0.001
Colorectal and gastric cancer	10-11	50	1443	4 (3.7 to 4.3)	Ref	
	8-9	81	1325	4.3 (3.9 to 4.7)	1.07 (0.74 to 1.56)	0.775
	≤7	88	975	6.5 (5.8 to 7.1)	1.65 (1.14 to 2.38)	0.008

Appendix 18. Adjusted major complication rates across hospital facility level

Adjusted major complication rates were calculated using generalised estimating equations (GEE) to account for clustering of patients in hospital and for potential confounders (WB tertile, age, sex, cancer type, ECOG performance status, ASA grade, disease stage, and operative urgency). Confidence intervals (CIs) and a P value for trend were fitted using the multilevel logistic regression model with the number of hospital facilities and all confounders as covariates

	Hospital facility level	Hospital number	Patient number	Adjusted major complication rate (95% CI)	Odds ratio	P value
All cancers	10-11	54	4038	8.6 (8.4 to 8.8)	Ref	
	8-9	87	2676	10.3 (10.1 to 10.6)	1.23 (1.04 to 1.45)	0.014
	≤7	97	2201	10.8 (10.4 to 11.1)	1.29 (1.08 to 1.53)	0.005
Colorectal and gastric cancer	10-11	50	1452	13.8 (13.4 to 14.2)	Ref	
	8-9	81	1334	14.6 (14.2 to 15.1)	1.07 (0.87 to 1.33)	0.550
	≤7	89	982	16.4 (15.7 to 17.0)	1.23 (0.98 to 1.54)	0.081

Appendix 19. Capacity to rescue patients following major complication following case-mix adjustment

Adjusted mortality rates after major complication were calculated using generalised estimating equations (GEE) to account for clustering of patients in hospital and for potential confounders (WB tertile, age, sex, cancer type, ECOG performance status, ASA grade, disease stage, and operative urgency). Confidence intervals (CIs) and a P value for trend were fitted using the multilevel logistic regression model with the number of hospital facilities and all confounders as covariates

	Hospital facility level	Hospital number	Patient number	Adjusted capacity to rescue (95% CI)	Odds ratio	P value
All cancers	10-11	43	366	84.7 (81 to 88.4)	Ref	
	8-9	65	283	72.8 (67.6 to 78)	0.48 (0.33 to 0.71)	<0.001
	≤7	62	227	75.3 (69.7 to 81)	0.55 (0.36 to 0.84)	0.005
Colorectal and gastric cancer	10-11	34	189	71.5 (68.6 to 74.4)	Ref	
	8-9	61	195	71.2 (68.4 to 74)	0.99 (0.64 to 1.55)	1
	≤7	53	165	60.4 (56.6 to 64.2)	0.62 (0.39 to 0.96)	0.033

Appendix 20. Absolute risk for 30-day mortality associated with four or more hospital facilities within each income group stratified by cancer type and sex

Estimates for age 60 years, performance status 1, ASA grade 2, cancer stage III, and elective surgery.

Hospital facility level	WB income (tertile)	Cancer type	Sex	Age (years)	ECOG performance status	ASA	Stage	Urgency	Predicted probability of death	Absolute risk difference
>3	Low/lower middle	Colorectal	Male	60	1	II	III	Elective	0.036 (0.020 to 0.055)	-
≤3	Low/lower middle	Colorectal	Male	60	1	II	III	Elective	0.060 (0.034 to 0.095)	0.024 (0.005 to 0.051, p=0.011)
>3	Upper middle	Colorectal	Male	60	1	II	III	Elective	0.018 (0.009 to 0.029)	-
≤3	Upper middle	Colorectal	Male	60	1	II	III	Elective	0.031 (0.015 to 0.053)	0.012 (0.002 to 0.029, p=0.009)
>3	High	Colorectal	Male	60	1	II	III	Elective	0.009 (0.004 to 0.015)	-
≤3	High	Colorectal	Male	60	1	II	III	Elective	0.015 (0.007 to 0.028)	0.006 (0.001 to 0.015, p=0.010)
>3	Low/lower middle	Colorectal	Female	60	1	II	III	Elective	0.028 (0.016 to 0.044)	-
≤3	Low/lower middle	Colorectal	Female	60	1	II	III	Elective	0.048 (0.025 to 0.076)	0.019 (0.004 to 0.040, p=0.010)
>3	Upper middle	Colorectal	Female	60	1	II	III	Elective	0.014 (0.007 to 0.023)	-
≤3	Upper middle	Colorectal	Female	60	1	II	III	Elective	0.024 (0.011 to 0.042)	0.010 (0.002 to 0.023, p=0.011)
>3	High	Colorectal	Female	60	1	II	III	Elective	0.007 (0.003 to 0.012)	-
≤3	High	Colorectal	Female	60	1	II	III	Elective	0.012 (0.005 to 0.022)	0.005 (0.001 to 0.012, p=0.013)
>3	Low/lower middle	Gastric	Male	60	1	II	III	Elective	0.044 (0.024 to 0.070)	-
≤3	Low/lower middle	Gastric	Male	60	1	II	III	Elective	0.074 (0.041 to 0.117)	0.028 (0.006 to 0.060, p=0.009)
>3	Upper middle	Gastric	Male	60	1	II	III	Elective	0.022 (0.011 to 0.038)	-
≤3	Upper middle	Gastric	Male	60	1	II	III	Elective	0.038 (0.018 to 0.069)	0.015 (0.003 to 0.036, p=0.016)
>3	High	Gastric	Male	60	1	II	III	Elective	0.011 (0.005 to 0.020)	-
≤3	High	Gastric	Male	60	1	II	III	Elective	0.019 (0.008 to 0.037)	0.007 (0.001 to 0.020, p=0.014)
>3	Low/lower middle	Gastric	Female	60	1	II	III	Elective	0.035 (0.018 to 0.057)	-
≤3	Low/lower middle	Gastric	Female	60	1	II	III	Elective	0.059 (0.030 to 0.093)	0.023 (0.005 to 0.048, p=0.008)
>3	Upper middle	Gastric	Female	60	1	II	III	Elective	0.017 (0.008 to 0.030)	-
≤3	Upper middle	Gastric	Female	60	1	II	III	Elective	0.030 (0.014 to 0.053)	0.011 (0.002 to 0.028, p=0.015)
>3	High	Gastric	Female	60	1	II	III	Elective	0.009 (0.004 to 0.016)	-
≤3	High	Gastric	Female	60	1	II	III	Elective	0.015 (0.006 to 0.029)	0.006 (0.001 to 0.016, p=0.014)

Appendix 21. Absolute risk for 30-day mortality in hospitals with more than three facilities within each income group stratified by cancer type and sex

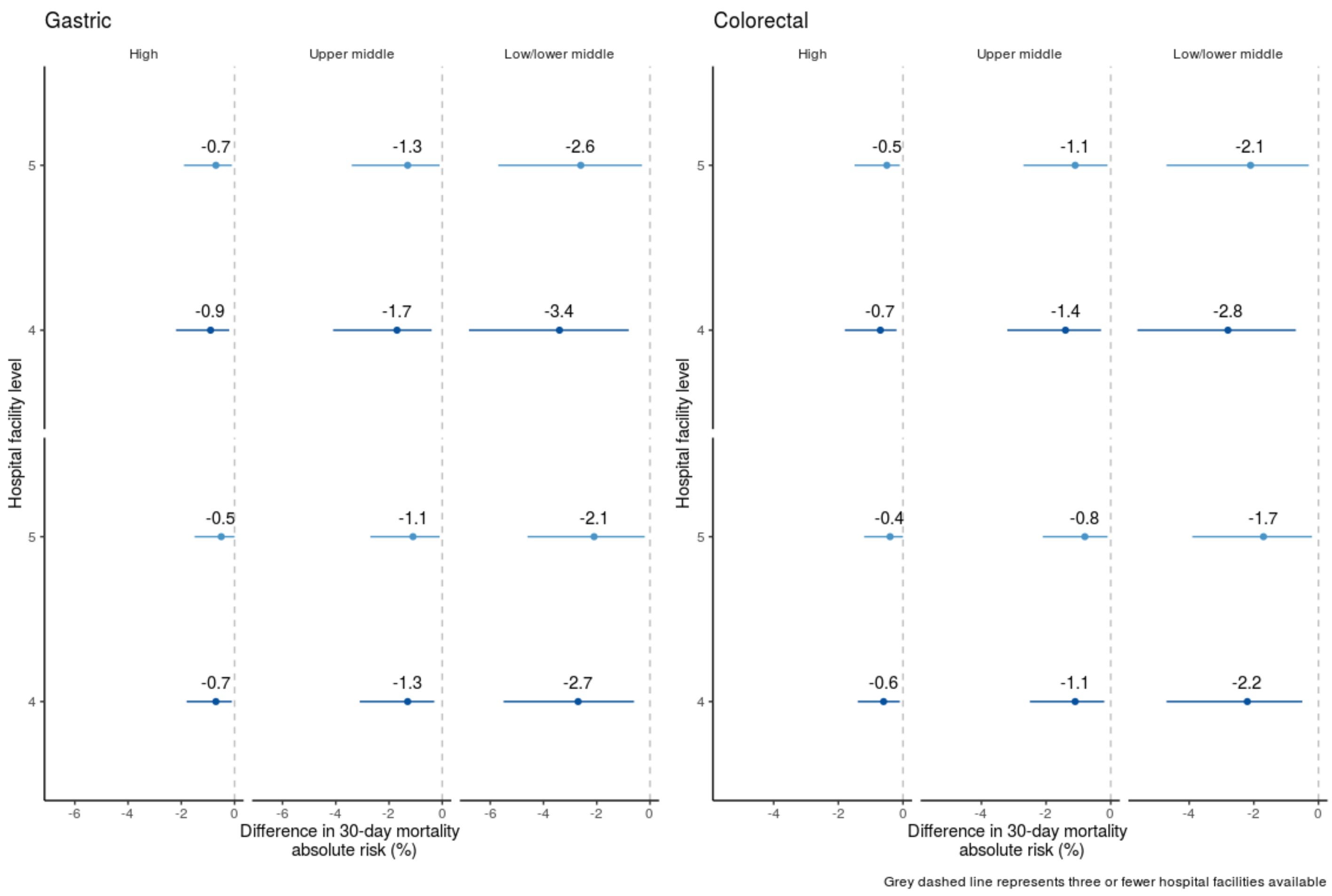

Estimates for age 60 years, performance status 1, ASA grade 2, cancer stage III, and elective surgery.

Appendix 22. Absolute risk for 30-day mortality in hospitals with more than three facilities within each income group stratified by cancer type and sex
Estimates for age 60 years, performance status 1, ASA grade 2, cancer stage III, and elective surgery.

Hospital facility level	WB income (tertile)	Cancer type	Sex	Age (years)	ECOG performance status	ASA	Stage	Urgency	Predicted probability of death	Absolute risk difference
5	Low/lower middle	Colorectal	Male	60	1	II	III	Elective	0.037 (0.021 to 0.058)	-
4	Low/lower middle	Colorectal	Male	60	1	II	III	Elective	0.030 (0.015 to 0.052)	-0.007 (-0.022 to 0.009, p=0.368)
≤3	Low/lower middle	Colorectal	Male	60	1	II	III	Elective	0.060 (0.033 to 0.095)	0.022 (0.003 to 0.049, p=0.025)
5	Upper middle	Colorectal	Male	60	1	II	III	Elective	0.019 (0.010 to 0.030)	-
4	Upper middle	Colorectal	Male	60	1	II	III	Elective	0.015 (0.007 to 0.028)	-0.003 (-0.011 to 0.004, p=0.378)
≤3	Upper middle	Colorectal	Male	60	1	II	III	Elective	0.030 (0.014 to 0.052)	0.011 (0.001 to 0.027, p=0.030)
5	High	Colorectal	Male	60	1	II	III	Elective	0.009 (0.005 to 0.016)	-
4	High	Colorectal	Male	60	1	II	III	Elective	0.008 (0.003 to 0.014)	-0.002 (-0.006 to 0.002, p=0.375)
≤3	High	Colorectal	Male	60	1	II	III	Elective	0.015 (0.007 to 0.029)	0.005 (0.001 to 0.016, p=0.023)
5	Low/lower middle	Colorectal	Female	60	1	II	III	Elective	0.029 (0.016 to 0.046)	-
4	Low/lower middle	Colorectal	Female	60	1	II	III	Elective	0.024 (0.011 to 0.041)	-0.005 (-0.018 to 0.007, p=0.368)
≤3	Low/lower middle	Colorectal	Female	60	1	II	III	Elective	0.047 (0.025 to 0.076)	0.017 (0.002 to 0.039, p=0.024)
5	Upper middle	Colorectal	Female	60	1	II	III	Elective	0.015 (0.007 to 0.024)	-
4	Upper middle	Colorectal	Female	60	1	II	III	Elective	0.012 (0.005 to 0.021)	-0.003 (-0.009 to 0.003, p=0.348)
≤3	Upper middle	Colorectal	Female	60	1	II	III	Elective	0.024 (0.011 to 0.041)	0.008 (0.001 to 0.022, p=0.031)
5	High	Colorectal	Female	60	1	II	III	Elective	0.007 (0.004 to 0.012)	-
4	High	Colorectal	Female	60	1	II	III	Elective	0.006 (0.002 to 0.011)	-0.001 (-0.005 to 0.002, p=0.362)
≤3	High	Colorectal	Female	60	1	II	III	Elective	0.012 (0.005 to 0.023)	0.004 (0.000 to 0.012, p=0.029)
5	Low/lower middle	Gastric	Male	60	1	II	III	Elective	0.046 (0.025 to 0.073)	-
4	Low/lower middle	Gastric	Male	60	1	II	III	Elective	0.038 (0.017 to 0.067)	-0.008 (-0.027 to 0.011, p=0.361)
≤3	Low/lower middle	Gastric	Male	60	1	II	III	Elective	0.073 (0.039 to 0.116)	0.026 (0.003 to 0.057, p=0.025)
5	Upper middle	Gastric	Male	60	1	II	III	Elective	0.023 (0.011 to 0.040)	-
4	Upper middle	Gastric	Male	60	1	II	III	Elective	0.019 (0.007 to 0.037)	-0.004 (-0.014 to 0.006, p=0.377)

≤3	Upper middle	Gastric	Male	60	1	II	III	Elective	0.037 (0.017 to 0.066)	0.013 (0.001 to 0.034, p=0.030)
5	High	Gastric	Male	60	1	II	III	Elective	0.012 (0.005 to 0.021)	-
4	High	Gastric	Male	60	1	II	III	Elective	0.009 (0.004 to 0.019)	-0.002 (-0.008 to 0.003, p=0.364)
≤3	High	Gastric	Male	60	1	II	III	Elective	0.019 (0.008 to 0.036)	0.007 (0.000 to 0.019, p=0.033)
5	Low/lower middle	Gastric	Female	60	1	II	III	Elective	0.036 (0.019 to 0.059)	-
4	Low/lower middle	Gastric	Female	60	1	II	III	Elective	0.030 (0.013 to 0.054)	-0.006 (-0.022 to 0.009, p=0.393)
≤3	Low/lower middle	Gastric	Female	60	1	II	III	Elective	0.058 (0.031 to 0.096)	0.021 (0.002 to 0.047, p=0.028)
5	Upper middle	Gastric	Female	60	1	II	III	Elective	0.018 (0.009 to 0.031)	-
4	Upper middle	Gastric	Female	60	1	II	III	Elective	0.015 (0.006 to 0.028)	-0.003 (-0.011 to 0.005, p=0.370)
≤3	Upper middle	Gastric	Female	60	1	II	III	Elective	0.029 (0.013 to 0.053)	0.010 (0.001 to 0.027, p=0.026)
5	High	Gastric	Female	60	1	II	III	Elective	0.009 (0.004 to 0.016)	-
4	High	Gastric	Female	60	1	II	III	Elective	0.007 (0.003 to 0.015)	-0.001 (-0.006 to 0.002, p=0.377)
≤3	High	Gastric	Female	60	1	II	III	Elective	0.015 (0.006 to 0.030)	0.005 (0.000 to 0.016, p=0.029)

Appendix 19. Absolute risk for 30-day mortality associated with four or more hospital facilities within each income group stratified by cancer type and sex for higher risk surgical patients (ASA grade ≥3)

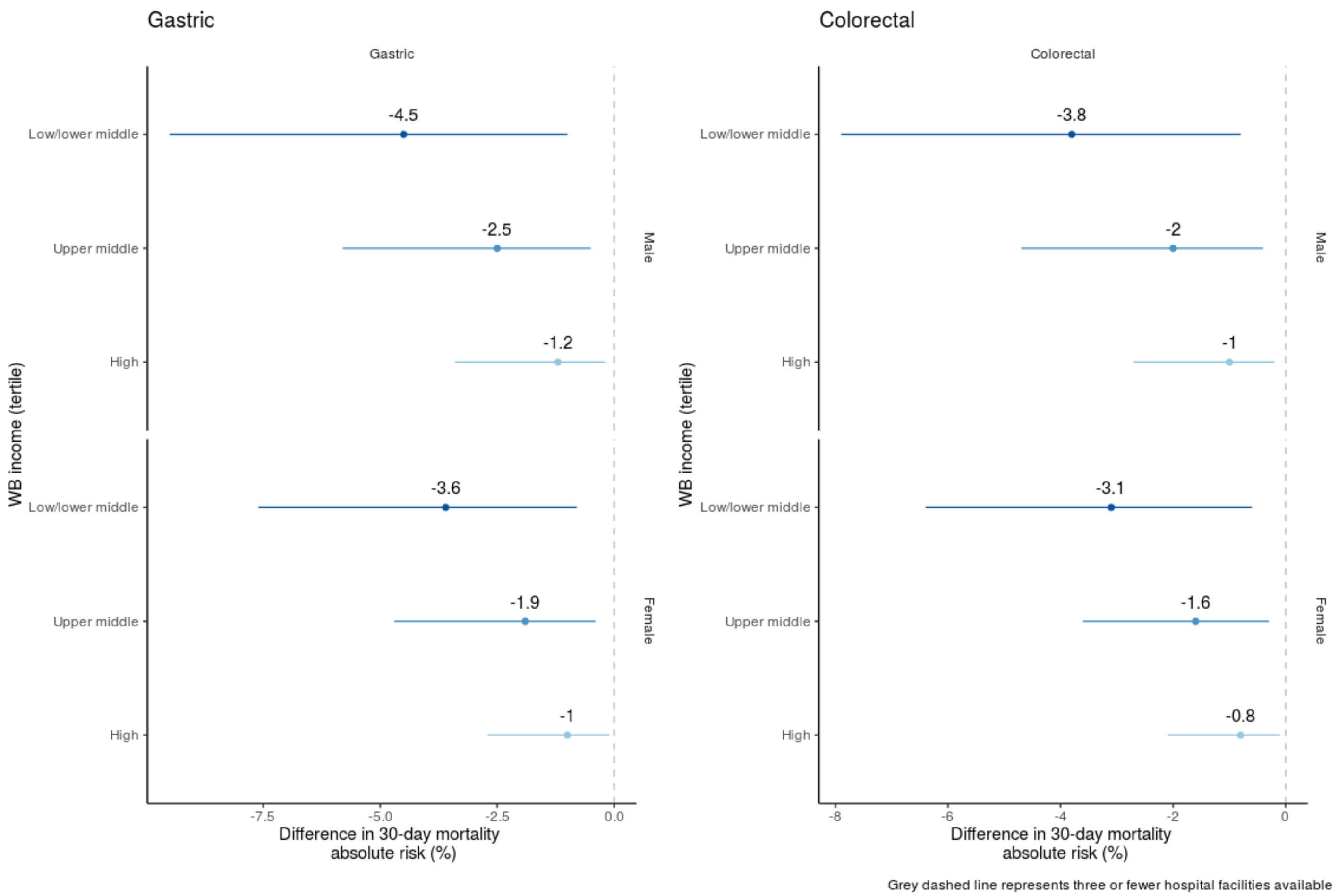

Estimates for age 70 years, performance status 3 or 4, ASA grade ≥3, cancer stage III, and elective surgery.

Appendix 20. Absolute risk for 30-day mortality associated with four or more hospital facilities within each income group stratified by cancer type and sex for higher risk surgical patients (ASA grade ≥3)

Estimates for age 70 years, performance status 3 or 4, ASA grade ≥3, cancer stage III, and elective surgery.

Hospital facility level	WB income (tertile)	Cancer type	Sex	Age (years)	ECOG performance status	ASA (≥3)	Stage	Urgency	Predicted probability of death	Absolute risk difference
>3	Low/lower middle	Colorectal	Male	70	3/4	Yes	III	Elective	0.323 (0.183 to 0.485)	-
≤3	Low/lower middle	Colorectal	Male	70	3/4	Yes	III	Elective	0.418 (0.251 to 0.594)	0.093 (-0.005 to 0.185, p=0.061)
>3	Upper middle	Colorectal	Male	70	3/4	Yes	III	Elective	0.201 (0.107 to 0.329)	-
≤3	Upper middle	Colorectal	Male	70	3/4	Yes	III	Elective	0.275 (0.147 to 0.430)	0.069 (-0.002 to 0.151, p=0.058)
>3	High	Colorectal	Male	70	3/4	Yes	III	Elective	0.109 (0.054 to 0.183)	-
≤3	High	Colorectal	Male	70	3/4	Yes	III	Elective	0.156 (0.072 to 0.277)	0.044 (-0.001 to 0.113, p=0.058)
>3	Low/lower middle	Colorectal	Female	70	3/4	Yes	III	Elective	0.271 (0.147 to 0.423)	-
≤3	Low/lower middle	Colorectal	Female	70	3/4	Yes	III	Elective	0.359 (0.205 to 0.534)	0.084 (0.000 to 0.171, p=0.050)
>3	Upper middle	Colorectal	Female	70	3/4	Yes	III	Elective	0.164 (0.085 to 0.272)	-
≤3	Upper middle	Colorectal	Female	70	3/4	Yes	III	Elective	0.228 (0.116 to 0.371)	0.061 (-0.003 to 0.141, p=0.061)
>3	High	Colorectal	Female	70	3/4	Yes	III	Elective	0.087 (0.043 to 0.153)	-
≤3	High	Colorectal	Female	70	3/4	Yes	III	Elective	0.126 (0.057 to 0.232)	0.037 (-0.000 to 0.097, p=0.054)
>3	Low/lower middle	Gastric	Male	70	3/4	Yes	III	Elective	0.379 (0.218 to 0.551)	-
≤3	Low/lower middle	Gastric	Male	70	3/4	Yes	III	Elective	0.478 (0.294 to 0.650)	0.096 (-0.000 to 0.192, p=0.050)
>3	Upper middle	Gastric	Male	70	3/4	Yes	III	Elective	0.243 (0.125 to 0.406)	-
≤3	Upper middle	Gastric	Male	70	3/4	Yes	III	Elective	0.326 (0.174 to 0.516)	0.080 (-0.001 to 0.171, p=0.053)
>3	High	Gastric	Male	70	3/4	Yes	III	Elective	0.135 (0.064 to 0.237)	-
≤3	High	Gastric	Male	70	3/4	Yes	III	Elective	0.191 (0.086 to 0.343)	0.054 (0.000 to 0.134, p=0.048)
>3	Low/lower middle	Gastric	Female	70	3/4	Yes	III	Elective	0.322 (0.176 to 0.494)	-
≤3	Low/lower middle	Gastric	Female	70	3/4	Yes	III	Elective	0.417 (0.245 to 0.594)	0.093 (-0.003 to 0.188, p=0.054)
>3	Upper middle	Gastric	Female	70	3/4	Yes	III	Elective	0.201 (0.101 to 0.335)	-
≤3	Upper middle	Gastric	Female	70	3/4	Yes	III	Elective	0.274 (0.139 to 0.438)	0.071 (-0.001 to 0.155, p=0.052)
>3	High	Gastric	Female	70	3/4	Yes	III	Elective	0.109 (0.050 to 0.204)	-
≤3	High	Gastric	Female	70	3/4	Yes	III	Elective	0.155 (0.068 to 0.298)	0.043 (-0.001 to 0.119, p=0.054)

Authorship

Writing group

Stephen R Knight, Kathryn Chu, Marie Carmela Lapitan, Anna J Dare, Catherine A Shaw, Riinu Pius, Thomas M Drake, Lisa Norman, Adesoji O Ademuyiwa, Adewale O Adisa, Maria Lorena Aguilera, Sara W Al-Saqqa, Ibrahim Al-Slaibi, Aneel Bhangu, Bruce M Biccard, Peter Brocklehurst, Ainhoa Costas-Chavarri, Muhammed Elhadi, Cameron J Fairfield, Faustin Ntirenganya, J Edward Fitzgerald, Dhruv Ghosh, James Glasbey, Mark I. van Berge Henegouwen, J.C Allen Ingabire, T Peter Kingham, Ismaïl Lawani, Bettina Lieske, Richard Lilford, Kenneth A Mclean, Rachel Moore, Dion Morton, Dmitri Nepogodiev, Francesco Pata, Thomas Pinkney, Ahmad Uzair Qureshi, Antonio Ramos-De la Medina, Aya Riad, Hosni Khairy Salem, Joana Simões, Richard Spence, Neil Smart, Stephen Tabiri, Hannah Thomas, Thomas G Weiser, Malcolm West, John Whitaker, Ewen M Harrison

National Leads

Albania: Arben Gjata; **Argentina**: Maria Marta Modolo; **Australia**: Sebastian King, Erick Chan; **Bangladesh**: Sayeda Nazmun Nahar; **Barbados**: Ade Waterman; **Belgium**: Dominique Vervoort; **Benin**: Ismaïl Lawani; **Botswana**: Alemayehu Ginbo Bedada; **Brazil**: Bernardo De Azevedo, Ana Gabriela Figueiredo; **Bulgaria**: Manol Sokolov; **Burundi**: Venerand Barendegere; **Cameroon**: Gerald Ekwen; **Canada**: Arnav Agarwal, Anna Dare; **China**: Qinyang Liu; **Congo, Dem. Rep.**: Kalisya Luc Malemo, Jacques Bake; **Croatia**: Jakov Mihanovic; **Czech Republic**: Kamila Kunčarová, Julius Orhalmi; **Egypt**: Hosni Salem; **Estonia**: Jyri Teras; **Finland**: Aristotelis Kechagias; **France**: Alexis P Arnaud; **Germany**: Judith Lindert; **Ghana**: Stephen Tabiri; **Greece**: Vasileios Kalles; **Guatemala**: Maria-Lorena Aguilera-Arevalo, Gustavo Recinos; **Hungary**: Zsolt Baranyai; **India**: Basant Kumar, Harish Neelamraju Lakshmi, Sanoop Koshy Zachariah, Philip Alexander, Sunil Kumar Venkatappa, C Pramesh; **Indonesia**: Radhian Amandito; **Ireland**: Christina Fleming; **Italy**: Francesco Pata, Gianluca Pellino; **Jordan**: ahmed M. Altibi, Ibrahim Nour; **Kenya**: Intisar Hamdun; **Libya**: Muhammed Elhadi, Ali M. Ghellai; **Lithuania**: Donatas Venskutonis, Tomas Poskus, Justas Zilinskas; **Madagascar**: John Whitaker; **Malawi**: Precious Malemia; **Malaysia**: Yong Yong Tew; **Malta**: Elaine Borg, Sarah Ellul; **Mexico**: Antonio Ramos-De la Medina; **Morocco**: Fatima Zahraa Wafqui; **Namibia**: David W Borowski; **Netherlands**: Anne Sophie van Dalen; **New Zealand**: Cameron Wells; **Niger**: Harissou Adamou; **Nigeria**: Adesoji Ademuyiwa, Adewale Adisa; **Norway**: Kjetil Søreide; **Pakistan**: Ahmad Uzair Qureshi; **Palestine**: Ibrahim Al-Slaibi, Sara Al Saqqa, Osaid Alser, Haya Tahboub; **Paraguay**: Helmut Alfredo Segovia Lohse; **Peru**: Sebastian Shu Yip; **Philippines**: Marie Carmela Lapitan; **Poland**: Piotr Major; **Portugal**: Joana Simões, António Sampaio Soares; **Romania**: Matei Razvan Bratu; **Russian Federation**: Andrey Litvin, Armen Vardanyan; **Rwanda**: JC Allen Ingabire, Ainhoa Costas-Chavarri; **Saudi Arabia**: Ahmad Gudal, Naif Albati; **Serbia**: Jovan Juloski; **Singapore**: Bettina Lieske; **Slovenia**: Miran Rems; **South Africa**: Sarah Rayne, Stephanie Van Straten, Yoshan Moodley, Kathryn Chu, Rachel Moore; **Spain**: Irene Ortega Vázquez, Jaime Ruiz-Tovar; **Sri Lanka**: Kithsiri Janakantha Senanayake, Sujeewa Priyantha Bandara Thalgaspitiya; **Sudan**: Omer Abdelbagi Omer, Anmar Homeida; **Sweden**: Yucel Cengiz; **Switzerland**: Daniel Clerc; **Syrian Arab Republic**: Muhammad Alshaar; **Tunisia**: Hanen Bouaziz; **Turkey**: Yuksel Altinel; **Uganda**: Matthew Doe; **Ukraine**: Maryna Freigofer; **United Kingdom**: Ella Teasdale, Rakan Kabariti, Joshua Michael Clements, Stephen Richard Knight, Ahsan Ashfaq; **United States**: Ijeoma Azodo; **Uruguay**: Gabriela Wagner, Ivan Trostchansky; **Zambia**: Mayaba Maimbo, David Linyama.

Local Collaborators

* denotes Hospital Lead

Albania: Helidon Nina, Amanda Zeko (University Hospital Center Nene Tereza).

Argentina: Claudio Gabriel Fermani, Maria Marta Modolo, Santiago Villalobos (Hospital Luis Lagomaggiore); Federico Carballo, Pablo Farina, Sebastian Guckenheimer (Ignacio Pirovano).

Australia: Marilla Dickfos (Bundaberg Base Hospital); Ankit Ajmera, Chester Chong, Ralph Gourlay, Sikandar Hussaini, Yi Jia Lee, Adeeb Majid, Peter Martin, Rebecca Miles, Owen James Morris, Jamie Phua, William Ridley, Tarunpreet Saluja, Ryan Renxin Tan, Jen Teh, Anna Wells (Calvary Mater

Newcastle); Bharti Arora, Qaasim Dollie, Debbie Ho*, Yanru Ma, Omattage Mahasha Perera, Anthony Truong (Gold Coast University Hospital); Amanda Caroline Dawson*, Bryan Lim, Upuli Pahalawatta, Jacqueline Phan, Xiao-Ming Sarah Woon-Shoo-Tong, Andrea Yeoh (Gosford Hospital); Lillian Charman, Andrew Drane, Sharon Laura, Charmaine Chu Wen Lo, Amy Mozes, Rita Poon*, Hao Han Tan, Ellen Wall (Gosford Private Hospital); Prakshi Chopra, Jasmine De Giovanni, Bal Dhital, Brian Draganic, Alexander Duller, Jonathan Gani, Yao Kuan Goh, Jun Young Jeong, Brendan McManus, Prakash Nagappan, Peter Pockney, Anya Rugendyke, Mahsa Sarrami, Stephen Smith, Vanessa Wills, Hsu Ven Wong, Geoffrey Ye, Geoffrey Zhang (John Hunter Hospital); Ethan Brooker, Daniel Feng, Bonnie Lau, Carlin Ngai (Manning Base Hospital); Sarah Birks, David Gyorki, Jaime Otero de Pablos (Peter MacCallum Cancer Centre); Ali Abbosh, Chris Gillespie, Ahmed Mahmoud* (Princess Alexandra Hospital); Bianca Kwan, Joshua Lawson, Andrea Warwick (Redcliffe Hospital); Janne Bingham, Andrew J Cockbain, Nagendra Naidu Dudi-Venkata, Jordan Ellaby-Hall, Ben Finlay, Emily Humphries, Jade Pisaniello, Monique Pisaniello, Salma Salih, Tarik Sammour* (Royal Adelaide Hospital); Haidar Hadri Abd Wahab, April De Silva, Nicola Hayward, Kartik Iyer, Guy Maddern*, Gian Andrea Prevost (The Queen Elizabeth Hospital); Naga Annapureddy, Krishna Pranathi Settipalli, Jeremy Yeo (The Wesley Hospital); Lucy Hempenstall, Lily Pham, Shaun Purcell (Toowoomba Hospital); Cherry Talavera, Ashish I Vaska (University Hospital Geelong); Gurpreet Chaggar, Phillip Chrapko, Annelise Cocco, Sarah Michelle Crystal Jade Coulter-Nile, Grahame Ctercteko, James French, Houchen Gong, Martijn Gosselink, Thuvarahan Jegathees, Ivan Jin, Michelle Kalachov, Kathryn Kiefhaber, Katherine Lee, Jason Luong, Steven Phan, Henry Pleass, Kelly Veale, Zhi Zeng (Westmead Hospital); Angela Au, Ashe DeBiasio, Idy Deng, Jananee Myooran, Amrita Nair, Peter Stewart* (Wyong Public Hospital).

Austria: Anton Stift, Lukas Walter Unger, Kerstin Wimmer (General Hospital of Vienna).

Bangladesh: Nabila Ahmed, Syed Hasan, Saber Rahman (Bangladesh Medical College Hospital).

Barbados: Margaret O'Shea, Greg Padmore, Adrian Peters (Queen Elizabeth Hospital).

Belgium: Pietro Perduca, Guenda Pulcina, Nicolas Tinton (Grand Hopital de Charleroi - Site Saint-Joseph); Frederic Buxant, Elsa Dabin, Giulia Garofalo (Hôpitaux Iris Sud - Etterbeek-Ixelles).

Benin: Francis Dossou, Ismaïl Lawani (Centre Hospitalier Universitaire et Departemental Oueme Plateau); Freddy Houehanou Rodrigue Gnangnon, Yacoubou Imorou Souaibou (Centre National Hospitalier et Universitaire Hubert Koutoukou Maga).

Botswana: Alemayehu Ginbo Bedada*, Pako Motlaleselelo, Omphile Tlhomelang (Princess Marina Hospital).

Brazil: Igor Lima Buarque, Gustavo Mendonça Ataíde Gomes, Aldo Vieira Barros (Hospital Santa Casa de Misericordia de Maceio).

Bulgaria: Ilia Batashki, Nikolai Damianov, Vladislav Stoyanov (Medical Institute of Ministry of Interior); Dragomir Dardanov, Svilen Maslyankov, Plamen Petkov, Manol Sokolov, George Todorov, Evgeni Zhivkov (University Hospital Alexandrovska); Aygulya Akisheva, Miguel Angel Castilla Moreno, Geno Genov, Ivelina Ilieva, Tsvetomir Ivanov, Martin Karamanliev, Azhar Khan, Emil Mitkov, Tsanko Yotsov (University Hospital Dr Georgi Stranski); Boyko Atanasov, Nikolay Belev, Mihail Slavchev (University Hospital Eurohospital).

Burundi: Carlos Nsengiyumva (Kamenge Military Hospital).

Cambodia: Elgan Jones, Simon Stock (World Mate Emergency Hospital).

Cameroon: Gerald Ekwen, Steve Kyota (Baptist Hospital); James Brown, Tresor Mabanza K., Lemery Nigo Samuel, Chidi Otuneme, Ngwang Prosper, Franklin Umenze (Mbingo Baptist Hospital).

Canada: Marylise Boutros, Natasha Caminsky, Sinziana Dumitra, Richard Garfinkle, Dominique Morency, Ebram Salama (Jewish General Hospital); Alexander Banks, Lorenzo Ferri, Haitian He, Amit Katz, Alexander Sender Liberman, Sarkis Meterissian, Allison Pang, Elena Parvez (McGill University Health Center); Arnav Agarwal, Anna Dare, Usmaan Hameed, Fahima Osman, Sangita Sequeira (North York General Hospital); Natalie Coburn, Anna Dare, Alisha Jaffer, Paul Karanicolas (Sunnybrook Hospital); Matthew Mosseler, Reilly Musselman (The Ottawa Hospital).

China: Xinyuan Liu, Ching Wan Yip (Huashan Hospital affiliated to Fudan University).

Colombia: Juan Sebastian Garces-Otero, Carolina Guzman, Sebastian Sierra, Andres Uribe Valencia (CES Clinic); Paulo Andrés Cabrera Rivera, Saul Camelo, Andrea Gonzalez, Alejandro González-Orozco, Manuel Santiago Mosquera Paz, Carlos J- Perez Rivera (Fundacion Cardioinfantil-IC); Felipe Gonzalez, Andres Isaza-Restrepo, Laura Nino- Torres (Hospital Universitario Mayor Méderi); Natalia Arias Madrid, Maria Clara Mendoza Arango, Sebastian Sierra (Hospital Universitario San Vicente Fundacion).

Congo, Dem. Rep.: Jacques Bake, Justin Tsandiraki (HEAL Africa Hospital).

Croatia: Damir Jemendžić, Branislav Kocman, Oliver Šuman (Clinical Hospital Merkur); Renata Canic, Darko Jurišić, Ivana Karakas, Ana Krizanovic Rupcic, Vlatka Pitlovic, Josip Samardžić (General Hospital Dr. Josip Bencevic); Mario Kopljar (University Hospital Center Sestre milosrdnice); Ivan Bacic, Edgar Domini, Robert Karlo, Jakov Mihanovic*, Danijela Miljanić, Andrea Simic (Zadar General Hospital).

Czech Republic: Mariam Ahmed, Majdi Al Nassrallah, Rabiya Altaf, Talal Amjad*, Ruba Eltoum, Heba Haidar, Alhassan Hassan, Omar Khalil, Marwan Qasem, Rommel Ramesh, Gautham Sajith, Maham Wisal (Charles University Hospital); Jan Žatecký* (Slezská nemocnice v Opavě, p.o.); Michele Bujda, Katerina Jirankova, Ales Paclik (The General University Hospital in Prague).

Egypt: Aya Abdallah, Mariam Abdulgawad Almogy, Esraa Ayman El-sawy, Ahmed Moustafa ElFayoumy, Nourhan Elghareeb, Nourhan Ahmed Esmat, Ahmed Fadel, Abdullah Habater, Heba Hamdy, Amr Hefni, Marwa Kamal, Norhan Mohamed Abobakr, Ahmed Sayed, Nancy Shaker, Ehab Taha, Hoda Tharwat, Omar Zakaria (Ain Shams University Specialized Hospital); Ibrahem Abdelmotaleb, Ali Al-Dhufri, Hamza S. Al-Himyari, Enas El sheikh, Asmaa Eldmaty, Aya Elkhalawy, Ahmed M.Elkhashen, Kithara Magdy, Safa Mostafa, Habib Doutoum Sadia, Mohamed mahmoud Saleh, Dina Samir, Mohamed Yahia Mohamed Ali (Al-Tagamoh Hospital); Mahmoud A. Nassar, Samar Abdelhady, Aly Abdelrazek, Israa Abdelsalam, Aya El-Sawy, Eman Essam, Mohamed Gadelkarim, Khaled Ghaly, Mohamed Hassabalnaby, Rana Masarani, Nourhan Mohamed Shaaban, Ahmed Sabry, Menatalla Salem, Nourhan Akram Soliman, Diaaaldin Zahran (Alexandria Main University Hospital); Moustafa Ramadan Abou El.soud, Esraa Tarek Badr, Hala Borham, Nehal Elmeslemany, Mohammad Elsayed, Fawzia Elsherif, Sara Eslam, Gehad Gaber, Sondos Ibrahim, Yara Kamh, Abdelrahman Mahmoud, Shimaa gamal Mohamed, Eman Morshedy, Cinderella Omar, Fatima Salem Soliman (Alexandria Medical Research Institute); Shaza Abdelkawy, Naglaa Abdelmohsen, Mahmoud Abdelshakour, Ahmed Dahy, Norhan Gamal, Mohammed Gamal, Ahmad Hasan, Helal Hetta, Nehad Mousa, Mohamed Omar, Somia Rabie, Mahmoud Saad, Bakeer Saleh, Marwa Sayed Mohamed, Muhammad Shawqi (Assiut University Hospital); Heba Abdelhady Mousa, Mostafa Alnoury, Mohamed Elbealawy, Ahmed Elshafey, Muhammad Essam Ibrahim El Desouki Muhammad Ahmed, Mennatullah Ghonaim, Fawzy Hgag, Mohamed Ibrahim, Mahmoud Morsy, Mohamed Reda Loaloa, Ahmed Refaat, Hadeer Samir, Fatma Shahien, Mohamed Sobhy, Fathy Sroor (Banha University Hospital); Esraa Abdellatif, Marina Adel, Amr Abdelghani Afifi, Eman Afifi, Marco Antaky, Amr Dawoud, Naira El Zoghby, Amira El-remaily, Ali Abdelazez Elzanfaly, Ahmed Gadallah, Fatma Alzahraa Gamal, Omar Hashem, Shrouk Medhat Youssef, Aliaa Muhammad Attyah, Malak Munir, Omar Shazly, Esraa Taha, Karim Wilson (El Demerdash University Hospital); Sawsan Adel, Asmaa Ali, Esraa Eid, Esraa Elhelow, Marwa Elmahdy, Bassant Elshatby, Amany Hossam el-din Zakaria, Ahmad Hossny, Eman Ibrahim, Ahmed M.Yonis, Maram Metwalli, Basant Yousry, Esraa Zid (Gamal Abd El Nasser Hospital); Mina A Yacoub, Ahmed Abdelhakim, Nervana Abouelsoad, Mo'min Alkhatib, Ahmed Ashraf, Alaa Ashraf, Yasmin Elazab, Mahmoud Elfanty, Osama Elkabir, Mai Elsayed, Ahmed Elshimy, Hager Elsobky, John Eskander, Ahmed Gad, Ward Hamsho, Noura Khaled Abdelwahed, Menna Magdy, Dalia Moharam, Abeer Osama, Shereen Ramadan, Radwa Roum, Taqwa Sayed, Tarneem Shehada, Ahmed Mohy Zidan (Kasr Alainy Hospital, Faculty of Medicine, Cairo University); Khalid Abbas, Amr Ali, Mohamed Attia, Mohamed Balata, Ayman El Nakeeb, Mohamed Ibrahim Elsayed Elewaily, Ahmed Elfallal, Hossam Elfeki, Ahmed Elkhadragy, Sameh Emile, Helmy Ezzat, Hasnaa Hosni, Islam Mansour, Waleed Omar, Gehad Othman, Kareem Sadek, Mostafa Shalaby, Noura Shehab-Eldeen (Mansoura University Hospital); Rawda Anas khalifa, Helmy Badr, Mostafa Eldeep, Ahmed Eldeep, Amany Eldoseuky mohammed, Salwa Khallaf, Eman Magdy Hegazy, Rokia Mahmoud, Pola Mikhail, Mahmoud Morsi, Sara Mowafy, Dina Raafat, Amina Safy, Marwa Sera, Ahmed shible Sera (Menofiya University Hospital); Mostafa Salim Mohamed AbdAllah, Muhammad Abdelkader, Abdulrahman Osama Abdou, Ahmedgaber Ahmed, Shireen Gaafar, Fatma Ibrahim negm, Mina Lapic, Ahmed Maher, Hagar Mahmoud, Ahmed Mostafa, Mohamed Samir, Fatma Samy, Nourhan Semeda, Hind I. Shalaby (National Cancer Institute); Alaa El-taweel, Ahmed Galal Elnagar, Ahmed Gamal Hemidan, Mohamed Hussein, Ahmed.A. Kandil, Mf Moawad, Ayat Allah Nasser Hamamah, Mostafa Soliman (National Institute of Diabetes and Endocrinology Hospital); Mohamed Abdelkhalek, Noura Abdelmaksoud Tawakel, Ahmed Mohamed Abdelwahed, Alrawy Abdou, Khalid Atallah, Mohammed Yasser Elsherbeny, Eman Emara, Mohamed Hamdy, Omar Hamdy, Amira Haron, Salma Ismail, Islam Hany Metwally, Nihal

Mohamed Hamed Elgaml, Ahmed Nassar, Basel Refky, Mirna Sadek, Mahmoud Saleh, Asmaa Yunes, Mai Zakaria, Mohammed Zuhdy (Oncology Center Mansoura University); Notila Fayed, Mohammed Mustafa Hassan Mohammed (Zagazig University Hospitals).

Estonia: Sander Kütner, Priit Melnik, Indrek Seire, Jyri Teras, Toomas Ümarik (The North Estonia Medical Centre).

Finland: Eppu Ainoa, Verner Eerola, Hanna Koppatz, Laura Koskenvuo, Ville Sallinen, Sini Takala (Helsinki University Hospital); Jevgeni Katunin, Aristotelis Kechagias, Arto Turunen (Kanta-Hame Central Hospital).

France: Niki Christou, Muriel Mathonnet (CHU Limoges); Vincent Lavoue, Krystel Nyangoh Timoh, Lucie Soulabaille (CHU Rennes - Breast Surgery); Romain Lesourd, Aude Merdrignac, Laurent Sulpice (CHU Rennes - General Surgery); Benoît André, Elodie Chantalat, Charlotte Vaysse (CHU Toulouse); Bertrand Dousset, Sebastien Gaujoux, Gregory Martin (Hôpital Cochin - APHP).

Germany: Octavian Clonda, Domantas Juodis, Klaus Kienle, Andras Mravik, Samuel Palmer, Gabor Szabadhegyi (Rottal-Inn-Kliniken).

Ghana: Anita Eseenam Agbeko, Solomon Gyabaah, Frank Enoch Gyamfi, Nuhu Naabo, Atta Owusu senior, Joseph Yorke (Komfo-Anokye Teaching Hospital); Frank Owusu (St. Patrick's Hospital); Francis Abantanga, Theophilus Teddy Kojo Anyomih, Abdul-Jalilu Mohammed Muntaka, Emmanuel Owusu Abem, Mohammed Sheriff, Stephen Tabiri, Paul M. Wondoh (Tamale Teaching Hospital).

Greece: Dimitrios Balalis*, Dimitrios Korkolis (Agios Savvas Anticancer Hospital); Georgios Gkiokas, Eirini Pantiora*, Theodosios Theodosopoulos (Aretaieion Hospital); Argyrios Ioannidis*, Konstantinos Konstantinidis, Sofia Konstantinidou (Athens Medical Center); Nikolaos Machairas, Anna Paspala, Anastasia Prodromidou* (Attikon University General Hospital); Christos Chouliaras, Konstantinos Papadopoulos* (General Hospital of Nikaia); Ioannis Baloyiannis, Ioannis Mamaloudis, George Tzovaras* (General University Hospital of Larissa); Ioanna Akrida, Maria-Ioanna Argentou, Stylianos Germanos, Evangelos Iliopoulos, Ioannis Maroulis, George Skroubis, George Theofanis (General University Hospital of Patras); Christos Chatzakis, Orestis Ioannidis*, Lydia Loutzidou (George Papanikolaou General Hospital of Thessaloniki); Vasileios Kalles, Panagiotis Karathanasis, Nikolaos Michalopoulos, Charalampos Theodoropoulos, Dimitrios Theodorou, Tania Triantafyllou* (Hippocratio General Hospital); Zoe Garoufalia, Natasha Hasemaki, Michalis Kontos, Gregory Kouraklis, Stylianos Kykalos, Theodore Liakakos, Eustratia Mpaili, Alexandros Papalampros, Dimitrios Schizas, Athanasios Syllaios, Ekaterini Christina Tampaki*, Antonios Tsimpoukelis (Laiko University Hospital); Maria Ioanna Antonopoulou, Eirini Deskou, Dimitrios K. Manatakis*, Dimitrios Papageorgiou, Menelaos Zoulamoglou (Naval And Veterans Hospital); Christos Anthoulakis, Michalis Margaritis, Nikolaos Nikoloudis* (Serres General Hospital).

Guatemala: Veronica Campo, André Ceballos, Mario-Andrés Flores, Waleska Giron, Donghyun Ko, Gabriel Martinez, Gustavo Recinos*, Verónica Rivera Lara, Nataly Rueda, Andres Sanchez, Jorge Carlos Guillermo Tejeda Garrido (Hospital General De Enfermedades); Maria-Lorena Aguilera-Arevalo, Alvaro Eduardo Alvarez Rivera, Elvis Benjamin Bamaca Ixcajoc, Lilian Elizabeth Barreda Zelaya, Patricia Chacòn-Herrera, Ligia Margarita Corea Ruiz, Guillermo Echeverria-Davila, Mario Garcia, Danilo García, Edgar Fernando Gutiérrez Mayen, Noriega José, Nery Mazariegos, Diego Méndez, Michael Paniagua Espinoza (Hospital General San Juan De Dios).

Hungary: Zsolt Baranyai, David Bardos, Marton Benke, Kristof Illes, Balint András Kokas, Réka Szabó (1st Department of Surgery - Semmelweis University).

India: Akhila Appukuttan, Anjitha Asok, Vijaykumar D.k (Amrita Institute of Medical Sciences Hospital); Kapil Malik, Praveen Ravishankaran, Ritesh Tapkire (Cachar Cancer Hospital and Research Centre); Guru Moorthy, Joyner Abraham, Ramesh Muthuvel (Government Rajaji Hospital); John Alapatt, Abhay Kattepur, Nizamudheen Pareekutty (Malabar Cancer Centre); Mebanshanbor Garod, Caleb Harris, Cliff Wanniang (North Eastern Indira Gandhi Regional Institute of Health and Medical Sciences (NEIGRIHMS)); Ashish Gupta, Deepak Nehra, Sanjeev Parshad (Pandit Bhagwat Dayal Sharma Post Graduate Institute of Medical Sciences); Rajgopal Acharya, Rajendra Badwe, Manish Bhandare, Urvashi Jain, Karishma Kirti, Nita Nair, Shailesh Shrikhande, Purvi Thakkar (Tata Memorial Centre); Premkumar Anandan, Archana C S, Arun Holenarasipur Narasannaiah, Tejaswi Jagarlamudi, Sunil Kumar Venkatappa, Rashmi M R, Mallikarjuna Manangi, Abhishek Raghavendra, K. Seshagiri Rao, Vinay S, Vinay Sajjan, Aneesh Shenoy, Santhosh Shivashankar Chikkanayakanahalli, Kavya Tharanath, Sushmita V (Victoria Hospital).

Indonesia: Peter Adidharma, Raksheeth Agarwal, Radhian Amandito, Phebe Anggita Gultom, Ghafur Rasyid Arifin, Matthew Billy, Zatira Elfizri, Alessa Fahira, Devi Felicia, Triana Hardianti Gunardi*, Nadya Johanna, Nadia Rahmadiani Nugrahadi, Sonar Soni Panigoro, Siti Rahmayanti, Retta Catherina Sihotang (Dr Cipto Mangunkusumo National General Hospital); Santi Yuanita Brata, Hadi Winoto (Mardi Rahayu Hospital).

Iran, Islamic Rep.: Nastaran Barati, Manoochehr Karami, Hamidreza Khorshidi, Homa Naderifar (Besat Hospital).

Iraq: Mazin A. Abdulla (Basra Teaching Hospital).

Ireland: Maggie Coleman, Ronan J Doherty, Rob Hannon (Beacon Hospital); Brenda Murphy, Aine Stakelum, Des Winter (St Vincent's University Hospital); Lylas Aljohmani, Richard Farnan, Yeshey Seldon, Tanna Tan, Shriya Varghese (St. James's Hospital); Mohammad Alherz, Muaaz Ather, Mohammad Bajilan, Vivien Graziadei, Isobel Pilkington, Omar Quidwai, Paul Ridgway, Haaris Shiwani*, Abd al-Rahman Tahir (Tallaght Hospital); Eimear Blunnie, Daniel Burke, Niall Kennedy, Kate Macdonagh, Maeve O'Neill, Siobhan Rooney (University Hospital Galway).

Italy: Giuseppe Falco, Guglielmo Ferrari, Simone Mele, Gabriela Elisa Nita, Lara Ugoletti, Maurizio Zizzo (Arcispedale Santa Maria Nuova); Gianmaria Confalonieri, Giovanni Pesenti, Fulvio Tagliabue (ASST di Lecco - P.O di Lecco); Gianluca Baronio, Deborah Ongaro, Giacomo Pata (ASST Spedali Civili, Ospedale di Brescia); Bruno Compagnoni, Renato Salvadori, Lucio Taglietti (ASST Valcamonica Ospedale di Esine); Nicola D'Alessandro, Pierpaolo Di Lascio, Giovanni Pascale (Azienda Ospedaliera Regionale 'San Carlo'); Luca Bortolasi, Tommaso Campagnaro, Massimo Carlini, Giorgio Lisi, Davide Lombardi, Corrado Pedrazzani, Domenico Spoletini, Giulia Turri, Paola Violi (Azienda Ospedaliera Universitaria Integrata di Verona); Donato Francesco Altomare, Fabrizio Aquilino, Nicola Musa, Vincenzo Papagni, Arcangelo Picciariello, Leonardo Vincenti (Azienda Ospedaliero Universitaria Consorziale Policlinico Di Bari); Dario Andreotti, Savino Occhionorelli, Matteo Tondo (Azienda Ospedaliero-Universitaria Di Ferrara); Stefano Maria Massimiliano. Basso, Paolo Ubiali (Azienda Per L'assistenza Sanitaria N. 5 Friuli Occidentale); Riccardo Cirelli, Marco Enrico Mario Maino, Guglielmo Niccolò Piozzi (Casa di Cura Igea); Emanuele Picone, Rosa Scaramuzzo, Giovanni Sinibaldi (Fatebenefratelli Isola Tiberina); Alfonso Amendola, Lorenzo Anastasio, Luigi Bucci, Emanuele Caruso, Antonio Castaldi, Sara Di Maso, Vincenza Paola Dinuzzi, Giovanni Esposito, Maria Gaudiello, Mariano Cesare Giglio, Paola Antonella Greco, Gaetano Luglio, Andrea Manfreda, Ester Marra, Federica Mastella, Gianluca Pagano, Roberto Peltrini, Vincenzo Pepe, Michele Sacco, Viviana Sollazzo, Giovanni Spiezio (Federico II University of Naples); Ettore Cianchetti, Nunzia Menduni (Hospital G.Bernabeo); Michele Maria Carvello, Francesca Di Candido, Antonino Spinelli (Humanitas Research Hospital); Fabio Corsi, Luca Sorrentino (ICS Maugeri); Fabio Marino (IRCCS 'Saverio de Bellis'); Emanuele Luigi Giuseppe Asti, Luigi Bonavina, Emanuele Rausa (IRCCS Policlinico San Donato); Martina Asta, Andrea Belli, Francesco Bianco, Carmela Cervone, Paolo Delrio, Armando Falato, Andrea Fares Bucci, Rita Guarino, Ugo Pace, Daniela Rega (Istituto Nazionale Tumori Fondazione, Pascale-I.R.C.C.S.); Emilia De Luca, Gaetano Gallo, Giuseppe Sammarco, Giuseppe Sena, Giuseppina Vescio (Mater Domini University Hospital); Letizia Santandrea, Giampaolo Ugolini, Davide Zattoni (Ospedale degli Infermi di Faenza); Nicola Chetta, Gaetano Logrieco, Serafino Vanella (Ospedale Generale Regionale F. Miulli); Gianluca Garulli, Nicola Zanini (Ospedale Infermi di Rimini); Andrea Bondurri, Francesco Cammarata, Francesco Colombo, Diego Foschi, Giulia Maria Beatrice Lamperti, Anna Maffioli, Gianluca Matteo Sampietro, Al'ona Yakushkina, Gloria Zaffaroni (Ospedale Luigi Sacco Milano); Luca Ansaloni, Enrico Cicuttin, Maria Grazia Sibilla (Ospedale M. Bufalini); Harmony Impellizzeri, Marco Inama, Gianluigi Moretto (Ospedale Pederzoli); Sylvie Mochet, Elisa Ponte, Antonella Usai (Ospedale Regionale Umberto Parini); Stefano Mancini, Andrea Sagnotta, Luigi Solinas (Ospedale San Filippo Neri); Elisa Bolzonaro, Nicolò Tamini (Ospedale San Gerardo); Gianluca Curletti, Raffaele Galleano, Michele Malerba (Ospedale Santa Corona, Pietra Ligure (SV)); Sofia Campanella, Gianfranco Cocorullo, Francesco Colli, Paolino De Marco, Nicolò Falco, Tommaso Fontana, Leonel jospin Kamdem Mambou, Antonella La Brocca, Leo Licari, Brenda Randisi, Giovanna Rizzo, Giulia Rotolo, Giuseppe Salamone, Roberta Tutino, Paolina Venturelli (Policlinico Paolo Giaccone di Palermo); Stefano Malabarba, Alessandro Sgrò, Ivan Vella (Policlinico San Matteo); Bruno Cirillo, Daniele Crocetti, Giorgio De Toma, Pierfrancesco Lapolla, Andrea Mingoli, Paolo Sapienza (Policlinico Umberto I); Angela Belvedere, Stefania Bianchini, Margherita Binetti, Arianna Birindelli, Valeria Tonini (S.Orsola-Malpighi Hospital); Mauro Podda, Fabio Pulighe (San Francesco Hospital); Michele De Rosa (San Giovanni Battista Hospital); Lorenzo Bono, Felice Borghi, Paolo Geretto, Maria Carmela Giuffrida, Corrado Lauro, Alessandra Marano, Luca Pellegrino, Paola Salusso, Diego Sasia (Santa Croce and Carle Hospital); Michela Campanelli, Alberto Realis Luc, Mario Trompetto (Santa Rita Clinic, Vercelli); Roberto Cardia, Nicola Cillara, Antonio Nicola Giordano (Santissima Trinità - ATS Sardegna); Antonio Costanzo, Mario Alessandro Giovilli, Luca Turati (Treviglio Hospital); Silvestro Canonico, Gianluca Pellino, Guido Sciaudone, Francesco Selvaggi, Lucio Selvaggi (Universitá della Campania 'Luigi Vanvitelli', Naples).

Jordan: Nader Albsoul, Ahmad AlBsoul, Ala'a Aldeen Alkhatib, Osama Alsallaq, Justin Z. Amarin, Rami Ayoub, Isam Bsisu*, M S El Muhtaseb, Mohammad Jabaiti, Jamal Melhem, Ibrahim Nour, Yasmeen Z. Qwaider, Mohammad Hasan Salameh, Ahmad Suleihat, Haya H. Suradi (Jordan University Hospital); Mohammad Alammarin, Almoutuz Aljaafreh, Mohammad Bani hani*, Zeina Bani hani, Farah Bani Hani, Toqa Fahmawee, Shadi Hamouri, Cyrine Katanani, Ra'fat Tawalbeh, Tamara Tawalbeh, Hassan Zawahrah (King Abdullah University Hospital); Mohamad K. Abou Chaar, Lana Abusalem, Mahmoud Al-Masri*, Hani Al-Najjar, Lutfi Barghuthi (King Hussein Cancer Center).

Kenya: Zahra Ahmed, Adnan Maulana, Omar Ngotho (Coast Provincial General Hospital); Charbel Kamau, Aruyaru Stanley Mwenda (Consolata Hospital); Fridah Bosire, Elizabeth Mwachiro, Robert Parker, Ian Simel, Kimutai Sylvester (Tenwek Hospital).

Libya: Abdulmunem Ahmed Mustafa Althini, Sofian Elbarouni, Aya Elseed Elbeshina, Ahmed Gwea, Ans Malek, Wedad Albashir Masoud Farag (Alkhadra Hospital); Abdulwahab Abdalei, Abu Baker Abdel Malik*, Areej Abo-khammash, Ma'aly Abuhlaiga, Nour Adnan, Marwa Albaggar, Asma Alfitory, Asma Aljanfi, Fakhruddin Almuzghi, Zohoor Altumei, Fatima Alzabti, Hana Ashoushan, Mohamed Assalhi, Joma Azzubia, Sondos Bnhameıda, Malik Delhen, Houssein Elshafei, Hana Elteir, Fatima Esbaga, Abdel Aziz Gobbi, Fatma Hamouda, Hamdan Hilan, Rania Ismail, Fieruz Jebran, Muataz Kasbour, Galia Maderi, Saja Mohammad, Burooj Mohammed, Habib Murtadi, Hamassat Mustafa, Mohamed Rajab, Sarah Trenba, Mariam Wafaa (Misurata Cancer Center); Eman Al Sagheir, Alabas Almigheerbi, Ahmed Alzahaf, Sumayyah Ghayth Bahroun*, Najah Ben Dallah, Mahmoud Elshaibani, Haitem Eswaye, Maha Karar, Samah Omar, Eman Younes, Maha Younes, Dafer Zreeg (National cancer institute); Saleh Abujamra, Firas Ashour, Mala Elgammudi, Wesal Omar F. Aljadidi, Enas Saddouh, Randa Sharif (New Bridge Hospital); Aya Alabuzidi, AbdulMawlay Alwerfally, Sarra Aribi, Fatma Bibas, Taha Elfaituri, Yasmine Elhajjaji, Ala Khaled, Wegdan Khalil, Tesneem Layas, Enas Soula, Ahmed Tarek (Tripoli Central Hospital); Muad fathi khalleefah Abu hallalah, Saleh Abujamra, Hazem Abdelkarem Ahmed*, Tagwa Alsharef, Abdulsalam Ali Ben Saoud, Tasnim El Gharmoul, Ahmed Elhadi, Safa Elrais, Abdulhalim Shebani, Heba Zarti, Asaid Zeiton (Tripoli Medical Center).

Lithuania: Marijus Ambrazevicius*, Nerijus Kaselis, Migle Stakyte (Klaipeda Republic Hospital); Oleg Aliosin, Agne Cizauskaite, Sarunas Dailidenas, Vitalijus Eismontas, Migle Kybransiene, Vitalija Nutautiene, Narimantas Samalavicius, Dainius Simcikas*, Algirdas Slepavicius, Albinas Tamosiunas, Nerijus Ubartas, Paulius Zeromskas (Klaipeda University Hospital); Saulius Bradulskis, Edvinas Dainius, Juozas Juočas, Egle Kubiliute, Juozas Kutkevičius, Aurimas Opolskis, Audrius Parseliunas, Andrejus Subocius, Donatas Venskutonis, Egle Virbickaite, Diana Zuikyte (Lithuanian University of Health Sciences Kaunas Clinical Hospital); Algirdas Bogusevicius, Kristina Buzaite, Daiva Čepulienė, Ieva Cesleviciene, Vaidotas Cesna, Jolanta Gribauskaite, Povilas Ignatavicius, Mantas Jokubauskas, Monika Liugailaitė, Ernest Margelis, Ruta Mazelyte, Lina Pankratjevaitė, Matas Pažusis, Agne Rackeviciute, Justina Saladyte, Monika Škimelytė, Vygintas Šlenfuktas, Monika Sudeikyte, Algimantas Tamelis, Tomas Vanagas*, Žygimantas Žumbakys (Lithuanian University of Health Sciences Kaunas Clinics); Aivaras Atkociunas, Audrius Dulskas, Justas Kuliavas (National Cancer Institute); Justas Birutis*, Sigitas Paškevičius, Mindaugas Šatkauskas (Republican Siauliai County Hospital); Donatas Danys, Matas Jakubauskas, Lina Jakubauskiene, Marius Kryzauskas, Vytautas Lipnickas, Gabija Makūnaitė (Vilnius University Hospital).

Madagascar: Fanjandrainy Rasoaherinomenjanahary, Herizo Rasolofonarivo, Luc Hervé Samison* (Joseph Ravoahangy Andrianavalona Hospital).

Malawi: Bitiel Banda, Precious Malemia, Vanessa Msosa (Kamuzu Central Hospital).

Malaysia: Ahmad Imran Ahmad Izzuddin, Andre Das (Hospital Kajang); Ying Yee Gan, Tan Shong Sheng, Jia yng Siaw (Hospital Sibu); Mohd Fadliyazid Ab Rahim, Dyg Zahratul Hamrak Abang Jamari, Nurfariza Che Husin, Muhd Yusairi Kamarulzaman, Yi Ping Lim, Nil Amri Mohamed Kamil, Mohd Razeen Mohd Hassan, Saidah Mohd Sahid*, Johari Mustafa, Elaine Hui Been Ng, Wan Khamizar Wan Khazim (Hospital Sultanah Bahiyah); Ng Chang Ern, P.g. Lingeshan, Syariz Ezuan Sulaiman (Hospital Sultanah Nora Ismail); Sue Ean Ang, Muhammad Navid Bin Mohamad Sithik, Yih Jeng Cheong, Mahadevan Deva Tata, Law Jia Xian, Aravinthan Kadravello, I-Ern Koh, Li-Yen Ng, Yuki Julius Ng We Yong, Kandasami Palayan, Chi Xuan Sam, Phuah Siow Jin, Jeremy Tan Ern Hwei, Yita Tang, Alvin Zubin Ter (Hospital Tuanku Ja'afar); Michael Pak-Kai Wong, Andee Dzulkarnaen Zakaria, Zaidi Zakaria (Hospital Universiti Sains Malaysia); Fitjerald Henry, Thyivya Kalaiselvan (Selayang Hospital); Muhammad Fairuz Shah Abd Karim, Mohamed Rezal Abdul Aziz, Nora Abdul Aziz, Tak Loon Khong, Peng Choong Lau, Hiong Chin Lim, April Camilla Roslani*, Jonathan Chen Ken Seak, Sui-Weng Wong, Lai Fen Wong, Leow Yeen Chin (University Malaya Medical Centre).

Malta: Mercy Chinemerem Anyanwu, Elaine Borg, Zachary Busuttil, Thomas Calleja, Kurt Lee Chircop, Ruth Cutajar, Andrew Michael Dimech, Sarah Ellul, Joseph Galea, Kiara Gascon Perai, Ruth Gatt, Lisa Kelman, Elizabeth Micallef, Favour Nwolu, Kim Sammut, Joanna Thompson, Sean Warwicker, Matthew Zammit (Mater Dei Hospital).

Mexico: Fernando Cordera, Efraín Cruz González, Jorge Sánchez-García (ABC Medical Center); Francisco José Barbosa Camacho, Francisco Javier Barrera López, Carlos Jose Zuloaga Fernandez del Valle (ANKER Oncologia Global Especializada); Eric Acosta, Iván Romarico González Espinoza, Perla Moreno (Hospital Angeles Puebla); Ana Olivia Cortes-Flores, Clotilde Fuentes Orozco, Alejandro Gonzalez Ojeda (Hospital de Especialidades, CMNO-IMSS); Samantha Corro Díaz González, Laura Martinez, Antonio Ramos-De la Medina (Hospital Español Veracruz); Bonifacio Mosqueda Amador, Armando Novoa, Dennet Arturo Olazo Espejo (Hospital Regional de Alta Especialidad); Alejandro Jimenez, Federico Lopez Rosales, Elva Gabriela Vanoye (Hospital Regional de Alta Especialidad de la Península de Yucatán); Luis Alberto Garcia Gonzalez, Roberto Carlos Miranda-Ackerman, Manuel Solano-Genesta (Hospital San Javier); Alethia Alvarez-Cano, Hector Hugo Romero-Garza (Hospital Universitario Dr José Eleuterio González); Heriberto Medina-Franco, Lorelí Mejía-Fernández, Noel Salgado-Nesme, Omar Vergara-Fernandez (Instituto Nacional de Ciencias Médicas y Nutrición 'Salvador Zubirán'); Guadalupe Montserrat Gutiérrez-Mota, Francisco Xavier Hernandez Vera, Anabella Llantada Lopez, Gilberto Morgan Villela, Felipe de Jesus Ramirez Padilla, Walezka Tapia Marin (Morgan Oncología Soluciones Integrales); Mónica Martínez Maldonado, Ramses Sánchez Suárez, José Manuel Troche (Unidad de Medicina de Alta Especialidad 'Adolfo Ruiz Cortines').

Morocco: Chaymae Benyaiche, Oumaima Outani (Centre Hospitalier Universitaire Ibn Sina Rabat); Souadka Amine, Amine Benkabbou, Anass Mohammed Majbar, Raouf Mohsine, Ali Rafik (Institut National d'Oncologie).

Myanmar: Thida Oung, Moe Moe Tin (North Okkalapa General Hospital).

Namibia: David W Borowski, Philipp Plarre (Mediclinic Cottage Hospital); David W Borowski, Philipp Plarre (Welwitschia Hospital).

Netherlands: Anna Alberga, Nina Sluiter, Jurriaan Tuynman (Amsterdam UMC VUmc); Robin Blok, Didem Cömert, Roel Hompes, Marianne Kalff, Merel Elisabeth Stellingwerf, Pieter Tanis, Mark van Berge Henegouwen, Elise Maria van Praag, Daan Wisselink (Amsterdam UMC, University of Amsterdam); Michael Gerhards, Josephine Lopes Cardozo, Emma Westerduin (Onze Lieve Vrouwe Gasthuis); Joske de Jonge, Aaw van Geloven, Kaz van Schilt (Tergooi Hospital); Frank den Boer, Simone Stoots, Stijn Vlek (Zaans Medisch Centrum).

New Zealand: Jamie Adams, Ibrahim S. Al-Busaidi, Gabrielle Budd, Seung il Choi, Michael Jen Jie Chu, Anurag Ganugapati, Lucy McKinstry, Rebecca Pascoe, Simon Richards, Kenrick Rosser, Annie Stevenson, Rebecca White (Christchurch Hospital); Shebani Farik, Jin Kwun, Ahmed Murad (North Shore Hospital); Sarah Cowan, Timothy Hall, Michael Hayton (Taranaki Base Hospital).

Niger: Laminou Malam Sani (Agadez Hospital); Souleymane Oumarou Garba* (Zinder Mother Child Center); Harissou Adamou, Ibrahim Amadou Magagi*, Oumarou Habou (Zinder National Hospital).

Nigeria: Halima Aliyu, Muhammad Daniyan, Tunde T. Sholadoye (Ahmadu Bello University Teaching Hospital); Lawal Abdullahi, Lofty-John Anyanwu*, Aminu Mohammad Mohammad, Abubakar Bala Muhammad, Abdurrahman Abba Sheshe, Ibrahim Suleiman (Aminu Kano Teaching Hospital); Alaba Adesina, Ajibola Awolowo, Clement Onuoha, Omotayo Salami, Ogechukwu Taiwo*, Agboola Taiwo (Babcock University Teaching Hospital); Stephen Kache, Jerry Godfrey Makama, Danjuma Sale (Barau Dikko Teaching Hospital); Olajide Abiola, Akinlabi Ajao, Anthony Ajiboye (Bowen University Teaching Hospital); Amarachukwu Etonyeaku, Julius Olaogun* (Ekiti State University Teaching Hospital); Ademola Adebanjo, Opeoluwa Adesanya (Federal Medical Centre); Michael Olatunji Afolayan, Olanrewaju Balogun, Ayomide Makanjuola, Samuel Nwokocha, Rufus Wale Ojewola, Thomas Olagboyega Olajide* (Lagos University Teaching Hospital); Adewale Aderounmu, Abdul-Rashid Adesunkanmi, Adewale Adisa*, Augustine Agbakwuru, Adeleke Akeem Aderogba, Olusegun Isaac Alatise, Olukayode Arowolo, Oladejo Lawal, Tajudeen Mohammed, Chinedu Ndegbu, Olalekan Olasehinde, Funmilola Wuraola (Obafemi Awolowo University Teaching Hospitals Complex); Akinbolaji Akinkuolie, Amarachukwu Etonyeaku, Arinzechukwu Mosanya (Obafemi Awolowo University Teaching Hospitals Complex Wesley Guild Hospital Unit); Omobolaji Ayandipo, Peter Elemile, Taiwo Akeem Lawal (University College Hospital); Samuel Ali SANI, Stephen Garba, Rebecca Hauwa SANI, Samson Olori*, Henry Onyebuashi, Ifeanyi Umoke

(University of Abuja Teaching Hospital); Adedire Adenuga, Ademola Adeyeye*, Olufemi Habeeb, Bashir Lawal, Abdulrasheed Nasir (University of Ilorin Teaching Hospital).

Norway: Eirik Kjus Aahlin, Didrik Kjønås, Elisabeth Myrseth (University Hospital Of North Norway).

Pakistan: Jibran Abbasy, Abdul Alvi, Omair Saleem (Aga Khan University); Asma Afzal, Anam Nazir (Ganga Ram Hospital); Muhammad Farooq, Ayesha Liaqat, Syed Asghar Naqi*, Ali Raza, Muzna Sarfraz, Muhammad Sarwar (King Edward Medical University, Mayo Hospital, Lahore); Muntaha Banglani, Ambreen Munir, Rahmat Sehrish (Liaquat University of Medical & Health Sciences); Bushra Ayub, Raza Sayyed (Patel Hospital); Amna Altaf, Saima Ayub, Ahmad Uzair Qureshi, Komal Saeed, Bilal Syed (Services Hospital Lahore); Sana Amir Akbar, Abdul Wahid Anwer, Ruqayya Naheed Khan*, Amina Iqbal Khan, Shahid Khattak, Sameen Mohtasham, Muhammad Asad Parvaiz, Aamir Ali Syed (Shaukat Khanam Memorial Cancer Hospital and Research Centre); Abdul Basit Ansari, Noman Shahzad (Sindh Institute of Urology and Transplantation); Tanwir Khaliq, Isbah Rashid, Shahzad Hussain Waqar (The Pakistan Institute of Medical Sciences).

Palestine: Hasan Abu Al-saleem, Amjad Abu Alqumboz, Mohammad Alqadi, Adham Amro, Rawan Assa, Eman Awesat, Rawan Ayyad, Mohammed Hammad, Ayat Haymony, Bassel Hijazi, Bara Hmeidat, Rowaa Lahaseh, Aseel Qawasmi, Alaa Rajabi, Mohammed Shehada, Sundus Shkokani, Yasmine Yaghi, Nadine Yaghi (Al Makassed Islamic Charitable Society Hospital Jerusalem); Mohammad AlZohour, Mohammad Farid, Yousef Mahmoud Habes, Wesam Juba, Yanal Nubani, Abdelrahman Rabee, Mohammad Sa'deh (Al-Ahli Hospital); Saeed Abed, Iyad Al basos, Mohammad Alswerki, Dina Ashour, Israa Awad, Samar Diab, Alaa El Jamassi, Sahar El-Kahlout, Somaya Elhout, Ahmed N K Hajjaj, Doaa Hasanain, Baraa Nabil hajjaj, Mohammed Obaid, Eman Saikaly, Ahmed Salhi (Al-Shifa Hospital); Hiba Al-Tammam, Murad Almasri, Muath Baniowda, Doha Beshtawi, Ali Horoub, Rami Misk, Bayan Mohammad, Rami Qasrawi, Tasnim Sholi (An-Najah National University Hospital); Samar Abu-Nimeh, Abrar Abu-srour, Sadi A. Abukhalaf, Samer Adawi, Barah Alsalameh, Kholoud Ayesh, Muawiyah Elqadi, Ahmad Hammouri, Fatima Karim Mustafa, Natalie Marzouqa, Shatha Melhem, Dima Miqdad, Balqees Mohamad, Mhammed Rawhi (Beit Jala Governmental Hospital (Al Hussein)); Ayman B. Abu Ahammala, Ahmed Abu Ataya, Israa Abu Jayyab, Samar Al-Shwaikh, Othman Alagha, Mohammed Alasttal, Haneen Awadallah, Mahmood Elblbessy, Jehad Fares, Akram Jarbou, Ibtisam Mahfouz, Moath A. Albahnasawi (European Gaza Hospital); Asmaa' Abo mahadi, Hasan Abuelhatal, Ayham Abuelqomboz, Abdelrahman Almoqayyad, Abdallah Alwali, Reem Balaawi, Mahmoud Hamouda, Mohammed Humeid, Abdullah Jedyan, Tasneem Mahmoud Abu hamam, Ghadeer Matar, ALi Salem, Tahani Samra, Nureddin Shaheen, Karam Shihada (Indonesian Hospital); Ayoob A.Nemer, Mahmoud Abu Al Amrain, Abdulwhhab Abu Alamrain, Najlaa Abu Jamie, Mohammed R. Abu-Rous, Nada Alfarra, Mohammed AlTaweel, Noor Alwhaidi, Ramadan Hamed, Bader Saqqa, Ahmad Shaheen (Nasser Hospital); Dana Aljaber, Loay Aljaberi, Malak Alwaheidi, Assef Jawaada, Hani Khaldi, Rami Qahoush, Jalil Qari, Rana Saadeh, Ahlam Salim, Aseel Yacoub (Palestine Medical Complex); Abbas Abbas, Rana Abu shua`ib, Baraa Abu Zainah, Mahmoud AbuSirrees, Basheer Babaa, Ola Barhoush, Asef Belal qadomi, Laith Daraghmeh, Reema Haji, Alaa Khatatbeh, Lana Khatib, Salsabeel Qarariah, Yara Quzmar, Khalil Safadi, Roqaya Salameh (Rafidia Hospital); Mohammad Hassan, Shifaa Herzallah, Loai Massad, Ahmed Nazzal, Ranin Nazzal (The Martyr Dr. Khalil Sulaiman Hospital (Jenin Governmental Hospital)).

Paraguay: Dennis Escobar, Gustavo Miguel Machain V, Agustin Rodriguez Gonzalez (Hospital de Clínicas, II Cátedra de Clínica Quirúrgica, Universidad Nacional de Asunción).

Peru: Jorge Emerson Chachaima Mar, Nathaly Olga Chinchihualpa Paredes, Vicente Cuba, Walter Lopez, Maria Milagros Niquen Jimenez*, Nestor Alberto Sanchez Bartra, Olenka Sapallanay Ojeda, Diego Sequeiros, Andrea Toscano Pacheco, María Vergara (Arzopispo Loayza National Hospital); Sol Abarca, Rodrigo Alcorta, Giuliano Borda-Luque, Ivan Edward Eusebio Zegarra, Claudia Luján López, Mirella Marrufo, Cinthya Mogrovejo, Andrea Nomura, Yamile Rodríguez Angeles, Maitza Rosario Vidal Meza, Gabriela Zavala* (Cayetano Heredia National Hospital); José Neiser Castillo Arrascue, Jomara Caroline Hidrogo Cabrera, José Julio Mariano Larrea vera, Miguel Osorio, Edgar Alcides Ylatoma Díaz* (Hospital Nacional Almanzor Aguinaga Asenjo).

Philippines: Mark Anthony Fontanilla, Joseph Roy Fuentes, Anna Leah Salazar (José R. Reyes Memorial Medical Center); Genieve Dominguez, Marc Paul Lopez, Shiela Macalindong, Mark Augustine Onglao, Arjel Ramirez, Marie Dione Sacdalan, Mayou Martin Tampo, Gemma Leonora Uy (Philippine

General Hospital, University Of The Philippines Manila); Jeremiah Mangahas, Kenneth Yabut (Quirino Memorial Medical Center); Joannes Paul Cañete, Bernalynn Eris Cansana, Ernes John Castro, Maria Kaiserin Lipana, Manuel Francisco Roxas, Vlu Jean Zara (The Medical City).

Poland: Maciej Chroł, Paula Franczak, Michał Orłowski (Ceynowa Hospital); Piotr Budzyński, Andrzej Budzyński, Pawel Bury, Agata Czerwińska, Jadwiga Dworak, Jacek Dziedzic, Michał Kisielewski, Jan Kulawik, Anna Lasek, Piotr Major, Piotr Małczak, Marcin Migaczewski, Michał Pędziwiatr, Magdalena Pisarska, Dorota Radkowiak, Mateusz Rubinkiewicz*, Anna Rzepa, Tomasz Skoczylas, Maciej Stanek, Katarzyna Truszkiewicz, Mateusz Wierdak, Marek Winiarski, Piotr Zarzycki, Anna Zub-Pokrowiecka (Jagiellonian University Medical College); Piotr Kowalewski, Rafał Roszkowski, Maciej Walędziak (Military Institute Of Medicine).

Portugal: Miguel Tomé, Sara Patrocinio, Ines Guerreiro* (Centro Hospitalar Barreiro Montijo, EPE); Filipe Almeida, Xavier de Sousa*, Nuno Monteiro (Centro Hospitalar de Setúbal); Maria Teresa Costa Santos*, Daniela de Oliveira, Marta Lopes Serra, Daniela Morgado, Christian Neves, Ana Carolina Oliveira, Alice Pimentel, Sofia Silva (Centro Hospitalar do Baixo Vouga); Márcia Carvalho* (Centro Hospitalar do Medio Ave); Lúcia Carvalho, Joana Magalhães, Leonor Matos* (Centro Hospitalar Entre o Douro e Vouga); Tânia Monteiro, Carlota Ramos*, Vanessa Santos (Centro Hospitalar Lisboa Norte); José Barbosa, Jose Costa-Maia, Vítor Devezas, Ana Fareleira, Cristina Fernandes, Diana Gonçalves, Henrique Mora, Marina Morais*, Fabiana Silva de Sousa (Centro Hospitalar Sao Joao); Sara Catarino Santos*, Ana Logrado, André Tojal (Centro Hospitalar Tondela-Viseu); Edgar Amorim, Miguel F. Cunha*, Ana Fazenda, João Pedro Melo Neves, Inês Isabel Sampaio da Nóvoa Gomes Miguel, Diogo Veiga (Centro Hospitalar Universitario do Algarve); José Azevedo, Hugo Cardoso Louro*, Mariana Leite (Centro Hospitalar Vila Nova de Gaia/Espinho); José Azevedo*, Maria Bairos Menezes, Bárbara Gama (Hospital da Horta, E.P.E.); Diana Brito, Marta Cristina Cruz Martins, André Graça e Magalhães, Ana Catarina Longras*, Rita Lourenço, Diana Matos (Hospital da Senhora da Oliveira); Luis Castro, Filipa Policarpo, Joana Romano* (Hospital de Egas Moniz); Mariana Leite, Cristina Monteiro*, Diogo Pinto (Hospital de Santa Luzia); Marina Duarte, Sónia Fortuna Martins*, Mariline Oliveira (Hospital de Santarem); Diogo Galvão, Lisandra Martins, Anaisa Silva, Viorel Taranu, Bárbara Vieira* (Hospital de Santo Espirito da ilha Terceira); Jessica Neves*, Simone Oliveira, Hugo Ribeiro (Hospital Distrital da Figueira da Foz); Margarida Cinza, Rosa Felix, Arnaldo Machado, Joana Oliveira, Joana Patrício*, Rita Pedroso de Lima, Mário Pereira, Miguel Rocha Melo, Cristina Velez (Hospital do Espirito Santo); Alberto Abreu da Silva, Mariana Claro*, Daniel Costa Santos, Andreia Ferreira (Hospital do Litoral Alentejano); Hugo Capote, Daniela Rosado, Filipa Taré (Hospital Doutor José Maria Grande); Oriana Nogueira, Miguel Ângelo, José Miguel Baiao, Andreia Guimarães, João Marques, Miguel Nico Albano*, Marta Silva, Ana Valente da Costa, Teresa Vieira Caroço (Hospital Geral - Centro Hospitalar de Coimbra); Sara Almeida Braga, Ines Capunge, Marta Fragoso*, João Guimarães, Bruno Pinto, João Ribeiro (Hospital Prof. Doutor Fernando Fonseca, E.P.E.); Miguel Angel, Guilherme Fialho*, Monica Guerrero (Hospital Santa Luzia Elvas); Filipa Campos Costa, Diogo Cardoso, Vasco Cardoso (Hospital Sao Francisco Xavier); Magda Alves, Inês Estalagem, Tiago Louro, Cláudia Marques*, Rita Martelo, Miguel Morgado (Hospital Vila Franca de Xira); Rita Canotilho, Ana Margarida Correia, Pedro Martins, Mariana Peyroteo* (IPO Porto); João Gomes, Rita Monteiro, Manuela Romano* (Unidade Local de Saúde de Castelo Branco); Daniela Macedo Alves, Rita Peixoto, Catarina Quintela* (Unidade Local de Saude de Matosinhos - Hospital Pedro Hispano); Maria João Jervis, Débora Melo, André Pacheco, Valter Paixão, Vera Pedro, Joana Pimenta, João Pimenta de Castro*, Ana Rocha (Unidade Local de Saude do Baixo Alentejo).

Romania: Mircea Beuran, Matei Razvan Bratu, Cezar Ciubotaru, Bogdan Diaconescu, Sorin Hostiuc, Ionut Negoi, Bogdan Stoica, NA (Emergency Clinical Hospital Bucharest).

Russian Federation: Evgeny Anokhin, Georgy Kuznetsov, Giorgi Oganezov, Fedor Paramzin, Ekaterina Romanova, Valeryan Rutkovskii, Vasilii Rutkovskii, Mikhail Shushval, Mikhail Zabiyaka (Kaliningrad Regional Hospital); Khasan Dzhumabaev, Valerii Ivanov, Zaman Mamedli (N.N.Blokhin Russian Cancer Research Center); Sergey Achkasov, Artem Balkarov, Elnur Nabiev, Marat Nagudov, Evgeny Rybakov, Karina Saifutdinova, Oleg Sushkov, Armen Vardanyan* (State Scientific Centre of Coloproctology).

Rwanda: Ainhoa Costas-Chavarri, Lule Joseph, Isaac Ndayishimiye (Rwanda Military Hospital); Jc Allen Ingabire, Ntirenganya Faustin, Alphonse Zeta Mutabazi, Jean Paul Mvukiyehe, Vizir J.P Nsengimana, Carine Uwakunda (University Teaching Hospital of Kigali).

Saudi Arabia: Mohammad Monir Abbas, Nouf Akeel, Murad Aljiffry, Kholoud Awaji, Ali Farsi, Ghader Jamjoum, Ahmad Khoja, Ashraf Maghrabi, Nadim Malibary, Mohammed Nassif, Abdulaziz Saleem, Abdullah Sultan, Wail Tashkandi, Hanaa Tashkandi, Nora Trabulsi (King Abdulaziz University Hospital).

Senegal: Mouhamadou Bachir Ba, Adja Coumba Diallo, Abdourahmane Ndong (Hopital Aristide Le Dantec).

Serbia: Vladica Cuk, Uroš Janković, Jovan Juloski (Zvezdara University Medical Center).

Singapore: Sharon Zhiling Koh, Frederick Koh, Kuok Chung Lee, Kai Yin Lee, Sean Lee, Wei Qi Leong, Bettina Lieske, Su Ann Lui, Prajwala Prakash (National University Hospital).

Slovenia: Jan Grosek, Gregor Norcic, Ales Tomazic (University Medical Centre).

South Africa: Nicolas Fitchat, Robert Jaich, Devorah Wineberg (Chris Hani Baragwanath Academic Hospital); Modise Zacharia Koto (Dr George Mukhari Academic Hospital); Daniella Baiocchi, Damian Clarke, Christina Johanna Steenkamp, Stephanie Van Straten (Greys hospital); Sharon Bannister, Adam Boutall, Galya Chinnery, Anna Coccia, Angela Dell, Parveen Karjiker, Christo Kloppers, Nicholas Loxton, Tumi Mabogoane, Francois Malherbe, Eugenio Panieri, Shreya Rayamajhi, Richard Spence, Tirsa van Wyngaard, Claire Warden (Groote Schuur Hospital); T E Madiba, Yoshan Moodley, Nivashen Pillay* (Inkosi Albert Luthuli Central Hospital); Savannah Brooks, Charlise Kruger, Lisa Hannah Van Der Merwe (Kalafong Academic Hospital); Ferhana Gool, Maahir Kariem (Mitchell's Plain District Hospital); Heather Bougard, Kathryn Chu, Nazmie Kariem, Fazlin Noor, Reantha Pillay, Leandi Steynfaardt (New Somerset Hospital).

Spain: Lucía González González, José Miguel Marín Santos, Paula Martín-Borregón, Javier Martínez Caballero, Cristina Nevado García, Pastora Rodriguez Fraga (12 de Octubre University Hospital); Gonzalo De Castro Parga, Maria Pilar Fernández Veiga, Lucía Garrido López, Hugo Infante Pino, Irene Lages Cal, Marta López Otero, Manuel Nogueira Sixto, Marta Paniagua García Señorans, Laura Rodríguez Fernández, Alejandro Ruano Poblador, Erika Rufo Crespo, Raquel Sanchez-Santos, Vincenzo Vigorita (Álvaro Cunqueiro Hospital); Ester Alonso Batanero, Dorisme Asnel, Isabel Cifrian Canales, Elisa Contreras Saiz, Irene De Santiago Alvarez, Tamara Díaz Vico, Sebastian Fernandez Arias, Daniel Fernández Martínez, Carmen García Bernardo, Luis Joaquín García Flórez, Carmen Garcia Gutierrez, Manuel García Munar, Carlos Alberto Márquez Zorrilla Molina, Marta Merayo, José Luis Michi Campos, Maria Moreno Gijon, Jorge L. Otero-Diez, Jose Luis Rodicio Miravalles, Lorena Solar-Garcia, Aida Suárez Sánchez, Nuria Truan (Central University Hospital Of Asturias); Cristina Alejandre Villalobos, Yurena Caballero Díaz, Marta Jimenez, Dacil Montesdeoca, Antonio Navarro-Sánchez*, Victor Vega (Complejo Hospitalario Universitario Insular-Materno Infantil); Juan Beltrán de Heredia, Zahira Gómez, Carlos Jezieniecki, Ana Patricia Legido Morán, Mario Montes-Manrique*, Mario Rodriguez-Lopez, María Ruiz Soriano, Jeancarlos Trujillo Díaz, Andrea Vazquez Fernandez (Hospital Clínico Universitario de Valladolid); Nuria Argudo, Miguel Pera, Laia Torrent Jansà (Hospital del Mar); Melody García Domínguez, Ignacio Goded, Marta Roldón Golet, Issa Talal El-Abur, Alejandra Utrilla Fornals, Vanesa Zambrana Campos (Hospital General San Jorge); Maria Del Mar Aguilar Martinez, Marina Bosch, Luis García-Catalá, Luis Sánchez-Guillén (Hospital General Universitario de Elche); Eva Artigau, Nuria Gomez Romeu, David Julià Bergkvist (Hospital Universitari de Girona Dr. Josep Trueta); Beatriz Espina Perez, Olga Morató, Carles Olona (Hospital Universitari de Tarragona Joan XXIII); Beatriz Diéguez, Alexander Forero-Torres, Manuel Losada (Hospital Universitario del Sureste); Segundo Gomez-Abril, Paula Gonzálvez, Rosario Martinez, Sergio Navarro Martínez, Carmen Payá-Llorente, Álvaro Pérez Rubio, Sandra Santarrufina Martinez, Juan Carlos Sebastián Tomás, Ramon Trullenque Juan (Hospital Universitario Doctor Peset); Alberto Gegúndez Simón, Paloma Maté, Maria Isabel Prieto-Nieto, Ines Rubio-Perez, Aitor Urbieta, Marina Vicario Bravo (Hospital Universitario la Paz); David Abelló, Matteo Frasson, Alvaro Garcia-Granero (Hospital Universitario y Politécnico La Fe); Alfredo Abad Gurumeta, Ane Abad-Motos, Elena Lucena-de Pablo, Beatriz Nozal, Javier Ripollés-Melchor, Rut Salvachúa (Infanta Leonor University Hospital); Esther Ferrero, Luis Garcia-Sancho Tellez, Irene Ortega Vázquez, Antonio L. Picardo, Jose Alberto Rojo López, Laura Patricia Zorrilla Matilla (Infanta Sofía University Hospital); Carmen Cagigas Fernandez*, Sonia Castanedo Bezanilla, José Estevez Tesouro, Maria Jose Fernandez-Diaz, Juan García Cardo, Marcos Gomez Ruiz, Erik Gonzalez-Tolaretxipi, Jaime Jimeno Fraile, Cristobal Poch, Montserrat Rodriguez-Aguirre, Noemí Troche Pesqueira, Maria Soledad Trugeda-Carrera (Marqués de Valdecilla University Hospital); Javier de la Torre, Ruth Blanco-Colino, Eloy Espin-Basany, Martin Espinosa-Bravo, Clara Morales Comas, Eduardo Reyes Afonso, Joaquín Rivero Déniz, Christian Siso Raber, Mireia Verdaguer Tremolosa (Vall d'Hebron University Hospital).

Sri Lanka: Pramodh Chandrasinghe, Sumudu Kumarage, Nimeshi Wijekoon Arachchilage (North Colombo Teaching Hospital); Kithsiri Janakantha Senanayake (Teaching Hospital Anuradhapura).

Sudan: Ahmed Abdalla Ahmed Elkamel (Ibrahim Malik Teaching Hospital); Mohammed A. Adam (Soba University Hospital); Mahmoud Saleh (University of Gezira Hospital).

Sweden: Nina Blomme, Anders Thorell, Fredrik Wogensen (Ersta Hospital); Andreas Älgå*, Dhirar Ansarei, Fuat Celebioglu, Göran Heinius, Linda Nigard, Emil Pieniowski (South General Hospital); Sandra Ahlqvist, Ida Björklund, Yucel Cengiz, Andreas Frånberg, Martina Håkansson (Sundsvall Hospital); Karin Adamo, Oskar Franklin, Malin Sund, Rebecca Wiberg (Umea University Hospital); Yvette Andersson, Abbas Chabok, Maziar Nikberg (Västmanlands Hospital Västerås); Alexander Kugelberg (Vrinnevi Hospital).

Switzerland: Claudia Canonica, Dimitrios Christoforidis*, Fabrizio Fasolini, Paolo Gaffuri, Mauro Giuliani, Francesco Meani, Sotirios Georgios Popeskou, Silvia Pozza, Wiebke Wandschneider (Ente Ospedaliero Cantonale); Lorenz Peterer, Lukas Werner Widmer*, Bernd Zimmermann (Kantonsspital Graubunden); Panagiotis Bakoleas, Iris Chanousi, Lydia Charalampidou, Lukasz Filip Grochola, Franziska Heid, Sotirios Ntaoulas, Michail Outos, Georgios Peros*, Hanna Podolska-Skoczek, Katharina Beate Reinisch, Christian Zielasek (Kantonsspital Winterthur); Daniel Clerc*, Nicolas Demartines, Jérôme Gilgien, Amaniel Kefleyesus, Pénélope St-Amour, Arnaud Toussaint (Lausanne University Hospital CHUV).

Syrian Arab Republic: Maryam Alhimyar, Bayan Alsaid, Amr Alyafi (Al-Assad University Hospital); Ahmad Alkhaledi, Basel Kouz, Ahmad Omarain (Al-Bairouni University Hospital); Yusra Al-Sabbagh, Haya Alkhatib, Samer Sara (Al-Mouwasat University Hospital); Ahmad Alhaj, Aghyad Danial, Lama Kadoura (Aleppo University Hospital); Sarah Maa Albared, Yamen Monawar, Louei Nahas (Damascus Hospital); Barook Abd, Ahmad Saad, Habib Wakkaf (Tishreen University Hospital).

Tunisia: Hanen Bouaziz, Hatem Bouzaiene, Montassar Ghalleb (Institut Salah Azaiez).

Turkey: Elif Akaydin, Ata Cem Akbaba, Onur Atakul, Ege Baltaci, Sevval Besli, Gökçen Burgu, Ulukan Cenal, Cansu de Muijnck, Hasan Can Demirkaya, Alper Dogruoz, Zeynep Ipek Gezer, Yasemin Gündoğdu, Merve Kara, Hasan Kürşad Korkmaz, Gökalp Kağan Kurtoğlu, Volkan Ozben, Berk Baris Ozmen, Ahmet Murat Pektaş, Eda Kübra Sel, Nilüfer Yenidünya (Acibadem Atakent Hospital); Fuat Baris Bengur, Berke Mustafa Oral, Tahir Koray Yozgatli (Acibadem Maslak Hospital); Seymur Abdullayev, Mehmet Emin Gunes, Nuri Alper Sahbaz (Bakirkoy Dr. Sadi Konuk Training And Research Hospital); Tuba Banaz, Kübra Kargıcı, Omer Faruk Kuyumcu, Erkan Yanıkoğlu, Merve Yeşilsancak, Duygu Yılmaz (Cerrahpasa Medical Faculty Istanbul University); Melik Kagan Aktas, Ahmet Rencuzogullari (Cukurova University Faculty of Medicine); Arda Isik (Erzincan University Hospital); Sezai Leventoğlu, Ali Yalçınkaya, Osman Yüksel (Gazi University Medical Faculty Hospital); Mustafa U Kalaycı, Yasin Kara, Inanc Samil Sarici (Kanuni Sultan Suleyman Training and Research Hospital); Alp Akin, Gökçe nur Alemdağ, Ekin Arslan, Bahadir Emre Baki, Muhammed Selim Bodur, Adnan Calik, Bahar Candas Altinbas, İrem Cihanyurdu, Oğuz Erkul, Burak Gül, Ali Guner, Beyza Köse, Anil Semiz, Şule Sevim, Serkan Tayar, Kadir Tomas, Ozan yavuz Tüfek, Serdar Türkyılmaz, Mehmet Uluşahin, Arif Usta, Reyyan Yildirim (Karadeniz Technical University Farabi Hospital); Sertaç Ata Güler, Ozan Can Tatar, Ecenur Varol (Kocaeli University Teaching Hospital); Busenur Kirimtay, Muhammed Uysal, Alp Yildiz (Memorial Ankara Hospital); Emin Kose (Okmeydanı Training And Research Hospital); Ahmet Burak Ciftci, Elif Çolak, Huseyin Eraslan, Gultekin Ozan Kucuk, Kürşat Yemez (Samsun Training and Research Hospital).

Uganda: Herman Lule* (Fort Portal Regional Referral Hospital); Mumbere Bienfait, Herman Lule* (Kampala International University Teaching Hospital); Emmanuel Bua, Matthew Doe*, Noella Okalany (Mbale Regional Referral Centre); Arianna Birindelli* (St. Kizito Hospital).

Ukraine: Maksym Basarab*, Oleksii Bielosludtsev, Maryna Freigofer, Kateryna Kolhanova, Kateryna Perepelytsia, Kateryna Romanukha, Dmytro Savenkov, Stanislav Siryi, Maksym Tereshchenko, Nezamai Viacheslav, Anton Volovetskyi (Dnepropetrovsk Regional Clinical Oncology Center); Andrey Kebkalo, Yegor Tryliskyy, Volodimir Tyselskiy (Kyiv Regional Clinical Hospital).

United Kingdom: Eilidh Bruce, Bing Lun Chow, Emma Iddles, Sarah McGuckin, Nicola Newall, George Ramsay, Parivrudh Sharma, Caitlin Stewart, Jeremy Wong (Aberdeen Royal Infirmary); Abdul Badran, Michael Bath*, Fanny Belais, Eman Butt, Kaustuv Joshi, Milan Kapur, Mike Shaw, Adam Townson, Christopher Yee Khang Williams (Addenbrooke's Hospital); Timothy Gray, Robert Greig, Mansoor Husain, Elspeth Murray, Ahmed Mustafa

(Borders General Hospital); Ashar Asif, Arya Gokul, Max Shah (Bristol Royal Infirmary); Mabel Temisanren Akitikori, Alexandros Charalabopoulos (Broomfield Hospital); Sophie Davidson, Sinead McNally, Shamil Rupani (Causeway Hospital); Fatema Juma, Sarah Catherine Mills, Laura Muirhead, Kate Sellars, Una Walsh, Oliver Warren (Chelsea and Westminster Hospital); Alice Chambers, Richard Hunt, Ella Teasdale (Cheltenham General Hospital); Stephen Boyce, Hannah Cornwall, Isabel Tol (Churchill Hospital); Eleftherios Orestis Argyriou, Nicola Eardley, Meical Povey (Countess of Chester Hospital); Joanna M S Aithie, Ahmer Irfan, Mari-Claire McGuigan, Robert Starr, Craig Russell Warren (Gartnavel General Hospital); Jess Archibald, Georgia Kirby, Ivan Kisyov (Gloucestershire Royal Hospital); Chun Kheng Khoo, Rachel Lee, Dana Photiou (Grantham and District Hospital); Rowan Davis, Uday Prasad, P Zichu Yang (Hairmyres Hospital); Jonathan Bird, Edmund Leung, Virginia Summerour (Hereford County Hospital); Chelise Currow, Jianshen Kiam, Gerald Jack Soon Tan (Ipswich Hospital); Anitha Muthusami, Ibifunke Pegba-Otemolu, Tomas Urbonas (John Radcliffe Hospital); Joseph Nunoo-Mensah, Edgaras Smolskas (King's College Hospital); Alex Boddy, Gianpiero Gravante, David Hunter (Leicester Royal Infirmary); David Andrew, Amanda Koh, Amari Thompson (Lincoln County Hospital); Lawrence Adams, Hollie A Clements, Kasun De Silva, Ogbonnia Ekpete, Seraj Haque, Scott Henderson, Bilal Ibrahim, Thummini Jayasinghe, Jennifer Livie, Keir Mailley, Gopikrishnan Nair, Daniel Tan (Ninewells Hospital); Caitlin Baggaley, Aleksander Dawidziuk, Bartosz Szyszka (Northwick Park Hospital); Charlotte Barter, Nirav Gandhi, Karen Hassell, Samantha Hitchin, Jennett Kelsall, Eva Nagy, Ashrafun Nessa, Lisa Whisker, Fady Yanni (Nottingham City Hospital); Mahmoud Ali, Deeksha Arora, Sunanda Hediwattege, Navam Kumarasinghe, Munir Rathore, Athula Tennakoon (Pilgrim Hospital); Syed Mustafa Ali Ahmad, Oreoluwa Bajomo, Fahema Nadira (Princess Alexandra Hospital); Valerio Celentano (Queen Alexandra Hospital); Aneel Bhangu, James Glasbey, Ewen Griffiths, Rama Santhosh Karri, Jason Kei Chak Mak, Dmitri Nepogodiev, Michelle Pipe (Queen Elizabeth Hospital Birmingham); Muhammad Iqbal Bhatti, Mohamed Rabie (Queen Elizabeth Hospital King's Lynn); Connor Boyle, David Hamilton, Aishath Mihuna, James Chean Khun Ng, Gary Nicholson, Agata Oliwa, Robert Pearson, Anna Rose, Shun Qi Yong (Queen Elizabeth University Hospital); Catherine Boereboom, Michael Hanna, Catherine Walter (Queens Medical Centre); Thomas Samuel Greensmith, Rachel Mitchell, Eimear Monaghan (Raigmore Hospital Inverness); James Crawford, Susan Moug (Royal Alexandra Hospital); James Blackwell, Hannah Boyd-Carson, Philip Herrod (Royal Derby Hospital); Omar Al-Allaf, Miriam Beattie, Cameron Bullock, Shivang Burman, Gemma Clark, Nicolas Flamey, Oliver Flannery, Alexander Harding, Ben Kodiatt, Samuel Lawday*, Shivani Mahapatra, Navin Mukundu Nagesh, Michael Ng, Dupinderjit Rye, Andrel Yoong (Royal Devon and Exeter Hospital); Laura Clark, Chris Deans, Monisha Edirisooriya, Cameron Fairfield, Ewen M Harrison (Royal Infirmary of Edinburgh); Emma Victoria Carrington, Tsz Lun Ernest Wong, Baasil Yusuf (Royal London Hospital); Carla Chamberlain, Kathryn Duke, Elizabeth Kmiotek (Royal Surrey County Hospital); Azel Botes, Natalie Condie*, Timothy Schrire, Reena Shah, Iolo Thomas-Jones, Charlotte Yates (Southmead Hospital); Natasha Anthony, Edward Matthews, Kapil Sahnan, James Tankel, Sally Tucker, Jasmine Winter Beatty, Paul Ziprin (St Mary's Hospital); William Duggan, Anastasia Kantartzi, Shruthi Sridhar (St Thomas' Hospital); Rachel Alys Khaw, Prakhar Srivastava, Charlotte Underwood (The Christie Hospital); Homero Alves do Canto Brum, Sharat Chopra, Laura Davis (University Hospital of Wales); Rebecca Hughes, Joshua Tulley (Walsall Manor Hospital); Justin Alberts, Thomas Athisayaraj, Mojolaoluwa Olugbemi (West Suffolk Hospital); Kasim Ahmad, Claudia Chan, Gavin Chapman, Hannah Fleming, Benjamin Fox, Julia Grewar, Kate Hulse, Duncan Rutherford, Mackay Sinead, Scott Smith, Doug Speake*, Peter G Vaughan-Shaw (Western General Hospital); Natasha Christodoulides, Simrit Kudhail, Matthew Welch (Wexham Park Hospital); Syed Muhibullah Husaini, Simon Lambracos (Worthing Hospital).

United States: Chikamuche Anyanwu, Rishi Suresh, Jimmy Scott Thomas (Baylor Scott&White Medical Center); Elizabeth Gleeson, Rebecca Platoff, Areeba Saif (Hahnemann University Hospital); Zachary Enumah, Eric Etchill, Alodia Gabre-Kidan (Johns Hopkins Hospital); Mitchell Bernstein, Francesco Maria Carrano, Joseph Connors, Patricio Lynn, Marcovalerio Melis, Elliot Newman (NYU Langone Medical Center); Deshka S Foster, Kenneth Perrone, Ashley Titan, Thomas G Weiser (Stanford Health Care); Sarwat Ahmad, Andrea Chao M. D. Bafford, Marco Dal Molin, Nader Hanna, Syed Nabeel Zafar (University of Maryland Hospital); Mark Hemmila, Lena Napolitano, Jane J Wong (University Of Michigan Medical Center); Julia Chandler, Lauren Wood, Sherry Wren (VA Palo Alto Hospital); Taylor Ottesen, Lucia You, Kristin Yu (Yale New Haven Hospital).

Uruguay: María del pilar Arciénega Yañez, Martin Ferreira Fernandes, Daniel González (Cooperativa Medica de Florida); Santiago Cubas, María Catalina González, Vanessa Zubiaurre (Hospital De Clinicas); Rodrigo Demolin, Nicolas Giroff, Pablo Sciuto (Hospital Espanol); Maite Campos, Gabriela Rodríguez Cantera, Gabriela Wagner (Hospital Maciel).

Zambia: Garg Deepika, Mayaba Maimbo, Elliot Simuchimba (Kitwe Teaching Hospital); Anadi Bulaya, Chali Chibuye, Bright Chirengendure (Ndola Central Hospital); Mary-Rose Kabale, Kizito Kabongo, David Linyama, James Munthali, Oliver Mweso, Francis Pikiti (University Teaching Hospital).

Data Validators

Australia: James Otieno (Calvary Mater Newcastle); Erick Chan (Gold Coast University Hospital); Log Tung Lai (Gosford Hospital); Brighid Blackman (Gosford Private Hospital); Sophie Richards (John Hunter Hospital); Suren Subramaniam (Peter MacCallum Cancer Centre); Rafid Karim (Princess Alexandra Hospital); Nathan Kok (Redcliffe Hospital); Yanni Dion Lee (Royal Adelaide Hospital); Shabina Ali (The Queen Elizabeth Hospital); Aanjaneya Sinha (The Wesley Hospital); Robert Corrigan (Toowoomba Hospital); Nicole Barnes (University Hospital Geelong); Florence Wong (Westmead Hospital); Grace Dennis (Wyong Public Hospital).

Austria: Julia Jedamzik (General Hospital of Vienna).

Barbados: Emil Phillips (Queen Elizabeth Hospital).

Belgium: Wivine Piette (Grand Hopital de Charleroi - Site Saint-Joseph); Marie Van hentenryck (Hôpitaux Iris Sud - Etterbeek-Ixelles).

Benin: Houenoukpo Koco (Centre Hospitalier Universitaire et Departemental Oueme Plateau); Souliath Lawani (Centre National Hospitalier et Universitaire Hubert Koutoukou Maga).

Botswana: Mamo Woldu Kassa (Princess Marina Hospital).

Brazil: Tainá Santos Bezerra (Hospital Santa Casa de Misericordia de Maceio).

Bulgaria: Petar Gribnev (University Hospital Alexandrovska); Dobromir Dimitrov (University Hospital Dr Georgi Stranski); Panche Krastev (University Hospital Eurohospital).

Cambodia: Sovannarith Oum (World Mate Emergency Hospital).

Cameroon: Divine Tim Bonghaseh (Baptist Hospital).

Canada: Maryam Al Farsi (Jewish General Hospital); Nourah Alsharqawi (McGill University Health Center); Arnav Agarwal (Sunnybrook Hospital).

Colombia: Veronica Acevedo (CES Clinic); Andrea Carolina Castillo Barbosa (Fundacion Cardioinfantil-IC); Felipe Giron (Hospital Universitario Mayor Méderi); Jimmy Paul Leon Rodriguez (Hospital Universitario San Vicente Fundacion).

Croatia: Darko Kučan (Clinical Hospital Merkur); Damir Rosko (General Hospital Dr. Josip Bencevic); Neven Barsic (University Hospital Center Sestre milosrdnice); Domagoj Župan (Zadar General Hospital).

Czech Republic: Amgad Hegazi (Charles University Hospital); Vendula Trunčíková (Slezská nemocnice v Opavě, p.o.); Vladimir Fryba (The General University Hospital in Prague).

Egypt: Mostafa Mohamed (Ain Shams University Specialized Hospital); Ahmed Sultan (Al-Tagamoh Hospital); Ahmed Nagi (Alexandria Main University Hospital); Abdallah Rashad Temerik (Assiut University Hospital); Mohamed Elemam Elshawy (El Demerdash University Hospital); Moustafa Ibrahim Mahmoud (Gamal Abd El Nasser Hospital); Shrouk Omar (Kasr Alainy Hospital, Faculty of Medicine, Cairo University); Mohamed Anwar (Mansoura University Hospital); Tarek Rageh (Menofiya University Hospital); Aya Elmokadem (National Cancer Institute); Khaled Gaballa (Oncology Center Mansoura University).

Estonia: Sandra Teppo (The North Estonia Medical Centre).

Finland: Antti Turunen (Helsinki University Hospital); Pasi Pengermä (Kanta-Hame Central Hospital).

France: Quentin Ballouhey (CHU Limoges); Damien Bergeat (CHU Rennes - General Surgery); Ariane Weyl (CHU Toulouse); Elisabeth Hain (Hôpital Cochin - APHP).

Ghana: Adam Gyedu (Komfo-Anokye Teaching Hospital); Edwin Yenli (St. Patrick's Hospital); Dorcas Osei-Poku (Tamale Teaching Hospital).

Greece: Vaia-Aliki Rompou (Agios Savvas Anticancer Hospital); Athanasios Zoikas (Athens Medical Center); Apostolos Gaitanidis (Attikon University General Hospital); Georgios Koukis (General Hospital of Nikaia); Konstantinos Perivoliotis (General University Hospital of Larissa); Panagiotis Tavlas (General University Hospital of Patras); Konstantinos Galanos-Demiris (George Papanikolaou General Hospital of Thessaloniki); George Zografos (Hippocratio General Hospital); Ioannis Karavokyros (Laiko University Hospital); Georgia Xanthopoulou (Naval And Veterans Hospital); Eirini Iordanidou (Serres General Hospital).

Guatemala: Fernanda Ayau (Hospital General De Enfermedades); Allan Garcia (Hospital General San Juan De Dios).

Hungary: Pekli Damján (1st Department of Surgery - Semmelweis University).

India: Deepender Wason (Pandit Bhagwat Dayal Sharma Post Graduate Institute of Medical Sciences); Ashika B L (Victoria Hospital).

Indonesia: Ervandy Rangganata (Dr Cipto Mangunkusumo National General Hospital).

Ireland: Prerna Kamath (St. James's Hospital); Donal B O'Connor (Tallaght Hospital).

Italy: Margherita Pinto (Azienda Ospedaliera Regionale 'San Carlo'); Fabrizio Perrone (Azienda Ospedaliero Universitaria Consorziale Policlinico Di Bari); Francesca Paola Tropeano (Federico II University of Naples); Francesca Troilo (Hospital G.Bernabeo); Daniela Bossi (ICS Maugeri); Dario Scala (Istituto Nazionale Tumori Fondazione, Pascale-I.R.C.C.S.); Lucrezia Pulitanò (Mater Domini University Hospital); Marcella Carella (Ospedale Generale Regionale F. Miulli); Andrea Pietrabissa (Policlinico San Matteo); Alice Gori (S.Orsola-Malpighi Hospital); Giorgio Giraudo (Santa Croce and Carle Hospital); Veronica De Simone (Santa Rita Clinic, Vercelli); Alfio Alessandro Russo (Treviglio Hospital); Bartolomeo Braccio (Universitá della Campania 'Luigi Vanvitelli', Naples).

Jordan: Raed Al-Taher (Jordan University Hospital); Sarah Athamneh (King Abdullah University Hospital).

Kenya: Andrea Parker (Tenwek Hospital).

Libya: Adnan Sawiee (Alkhadra Hospital); Amina Kattia (Misurata Cancer Center); Malik Salem (National cancer institute); Osama Tababa (New Bridge Hospital); Zuhour Shaeeb (Tripoli Central Hospital).

Lithuania: Vilius Syminas (Klaipeda Republic Hospital); Jonas Jurgaitis (Klaipeda University Hospital); Gytė Damulevičienė (Lithuanian University of Health Sciences Kaunas Clinical Hospital); Saulius Svagzdys (Lithuanian University of Health Sciences Kaunas Clinics); Tomas Poskus (Vilnius University Hospital).

Madagascar: Narindra Njarasoa Mihaja Razafimanjato (Joseph Ravoahangy Andrianavalona Hospital).

Malaysia: Ling Chieng Loo (Hospital Sibu); Ing Ching Tiong (Hospital Sultanah Bahiyah); Wan Farahiyah Wan Muhmad (Hospital Tuanku Ja'afar); Harinthiran Vijeyan (Hospital Universiti Sains Malaysia); Teoh Li Ying (University Malaya Medical Centre).

Malta: Gabriella Grech (Mater Dei Hospital).

Mexico: Rodrigo Arrangoiz (ABC Medical Center); Vania Brickelia Jimenez Ley (ANKER Oncologia Global Especializada); Daniel Arizpe (Hospital Angeles Puebla); Vania Brickelia Jimenez Ley (Hospital de Especialidades, CMNO-IMSS); Elizabeth Lagunes Lara (Hospital Español Veracruz); Elizabeth Victoria Castro López (Hospital Regional de Alta Especialidad); Jose Eaazim (Instituto Nacional de Ciencias Médicas y Nutrición 'Salvador Zubirán').

Netherlands: Marije Gordinou de Gouberville (Amsterdam UMC VUmc); Vivian Bastiaenen (Amsterdam UMC, University of Amsterdam); Simone Rottier (Tergooi Hospital).

New Zealand: Fouad Nahab (North Shore Hospital); Maria Yeonhee Ji (Taranaki Base Hospital).

Nigeria: Mohammed Seyoji (Ahmadu Bello University Teaching Hospital); Callistus Nwachukwu (Aminu Kano Teaching Hospital); Okechukwu Emeghara (Babcock University Teaching Hospital); Sayyid Egbunu Muhammed (Barau Dikko Teaching Hospital); Ayodeji Idowu (Ekiti State University Teaching Hospital); Olamiposi Sowemimo (Obafemi Awolowo University Teaching Hospitals Complex); Olakayode Ogundoyin (University College Hospital); Oluwatosin Akande (University of Ilorin Teaching Hospital).

Norway: Alexander Lott (University Hospital Of North Norway).

Pakistan: Maliha Nadeem (Ganga Ram Hospital); Ahsan Ali Laghari (Liaquat University of Medical & Health Sciences); Asif Loya (Shaukat Khanam Memorial Cancer Hospital and Research Centre); Hassan Mushtaq (Sindh Institute of Urology and Transplantation); Muhammad Tariq Abdullah (The Pakistan Institute of Medical Sciences).

Palestine: Baseel Abuhilal (Al Makassed Islamic Charitable Society Hospital Jerusalem); Mohammad Atawneh (Al-Ahli Hospital); Hamdan Hamdan (Beit Jala Governmental Hospital (Al Hussein)); Belal Alhabil (Indonesian Hospital); Abedelrahman Srour (Palestine Medical Complex); Ibrahim Mousa (Rafidia Hospital).

Paraguay: Luis Da Silva Medina (Hospital de Clínicas, II Cátedra de Clínica Quirúrgica, Universidad Nacional de Asunción).

Philippines: Marie Dione Sacdalan (José R. Reyes Memorial Medical Center); Marie Carmela Lapitan (Philippine General Hospital, University Of The Philippines Manila); Marie Dione Sacdalan (Quirino Memorial Medical Center); Marie Dione Sacdalan (The Medical City).

Poland: Katarzyna Bartosiak (Military Institute Of Medicine).

Portugal: Pedro Ferreira (Centro Hospitalar de Setúbal); Vítor Francisco (Centro Hospitalar do Baixo Vouga); Ricardo Lemos (Centro Hospitalar do Medio Ave); Luísa Frutuoso (Centro Hospitalar Entre o Douro e Vouga); Sara Fernandes (Centro Hospitalar Lisboa Norte); Telma Fonseca (Centro Hospitalar Sao Joao); Jorge Pereira (Centro Hospitalar Tondela-Viseu); Juan Rachadell (Centro Hospitalar Universitario do Algarve); Ana Torre (Centro Hospitalar Vila Nova de Gaia/Espinho); Filipe Madeira Martins (Hospital da Horta, E.P.E.); Ana Cristina Carvalho (Hospital da Senhora da Oliveira); Joana Rodrigues Ferreira (Hospital de Egas Moniz); Bruno Ribeiro da Silva (Hospital de Santa Luzia); Helena Devesa (Hospital de Santarem); Ana Vieira (Hospital de Santo Espirito da ilha Terceira); Inês Mónica (Hospital Distrital da Figueira da Foz); Margarida Amaro (Hospital do Espirito Santo); Diogo Sousa (Hospital do Litoral Alentejano); Marta Reia (Hospital Doutor José Maria Grande); João Louro (Hospital Geral - Centro Hospitalar de Coimbra); Ana Martins (Hospital Prof. Doutor Fernando Fonseca, E.P.E.); Joaquina Dominguez (Hospital Santa Luzia Elvas); Inês Santos (Hospital Sao Francisco Xavier); Nuno Miguel Freitas Oliveira (Hospital Vila Franca de Xira); José Carlos Pereira (IPO Porto); Pedro Silva-Vaz (Unidade Local de Saúde de Castelo Branco); Ligia Freire (Unidade Local de Saude de Matosinhos - Hospital Pedro Hispano); Ricardo Escrevente (Unidade Local de Saude do Baixo Alentejo).

Romania: Valentina Madalina Negoita (Emergency Clinical Hospital Bucharest).

Russian Federation: Dmitry Shakhmatov (State Scientific Centre of Coloproctology).

Rwanda: Yves Nezerwa (Rwanda Military Hospital).

Serbia: Radosav Radulovic (Zvezdara University Medical Center).

South Africa: Rachel Moore (Chris Hani Baragwanath Academic Hospital); Gareth Obery (Groote Schuur Hospital); Francois Viljoen (Inkosi Albert Luthuli Central Hospital); Tome Mendes (Mitchell's Plain District Hospital).

Spain: Antonio Suarez (12 de Octubre University Hospital); Enrique Moncada (Álvaro Cunqueiro Hospital); Maria Fernandez-Hevia (Central University Hospital Of Asturias); Carolina Curtis Martínez (Hospital General Universitario de Elche); Julia Maria Gil Garcia (Hospital Universitari de Girona Dr. Josep Trueta); Mariana González Zunzarren (Infanta Sofía University Hospital).

Sudan: Tarig Idris (Ibrahim Malik Teaching Hospital).

Sweden: Karolina Eklöv (South General Hospital); Oskar Grahn, Leila Amin (Umea University Hospital); Malin Blomqvist (Vrinnevi Hospital).

Switzerland: Costanza Ajani (Ente Ospedaliero Cantonale); Rebecca Kraus (Kantonsspital Graubunden); Nico Seeger (Kantonsspital Winterthur); Melissa Willemin (Lausanne University Hospital CHUV).

Syrian Arab Republic: Fadi Rayya (Al-Assad University Hospital); Mohammad Ayash (Al-Bairouni University Hospital); Raneem Msouti (Al-Mouwasat University Hospital); Israa Kannas (Aleppo University Hospital); Eias Abazid (Damascus Hospital); Asil Esper (Tishreen University Hospital).

Tunisia: Skander Slim (Institut Salah Azaiez).

Turkey: Akil Serdar Kavcar (Acibadem Atakent Hospital); Erman Aytac (Acibadem Maslak Hospital); Ahmet Cem Dural (Bakirkoy Dr. Sadi Konuk Training And Research Hospital); Ayse Ilker (Cerrahpasa Medical Faculty Istanbul University); Ismail Cem Eray (Cukurova University Faculty of Medicine); Eray Kurnaz (Erzincan University Hospital); Saygin Altiner (Gazi University Medical Faculty Hospital); Mustafa Deniz Tepe (Karadeniz Technical University Farabi Hospital); Can Şahin (Memorial Ankara Hospital); Evrim Savli (Samsun Training and Research Hospital).

Uganda: Aryon Innocent (Fort Portal Regional Referral Hospital); Lilian Babirye (Kampala International University Teaching Hospital).

Ukraine: Andrii Diachenko (Dnepropetrovsk Regional Clinical Oncology Center); Vladislav Hordoskiy (Kyiv Regional Clinical Hospital).

United Kingdom: Heather Curry (Aberdeen Royal Infirmary); Charlene Yat Che Chau (Addenbrooke's Hospital); Harry Robertson (Bristol Royal Infirmary); Arin Mahmoud (Broomfield Hospital); Hannah Lennon (Countess of Chester Hospital); Lynette Loi (Gartnavel General Hospital); Emily Kirkham (Gloucestershire Royal Hospital); Cameron McCann (Hairmyres Hospital); Daniel Watts (John Radcliffe Hospital); Binay Gurung (Leicester Royal Infirmary); Michael Wilson (Ninewells Hospital); Thomas Tribedi (Nottingham City Hospital); Eleonora Garofalo (Queen Alexandra Hospital); Baryab Zahra (Queen Elizabeth University Hospital); Scott MacDonald (Royal Alexandra Hospital); Ian Daniels (Royal Devon and Exeter Hospital); Nathan Ng (Royal Infirmary of Edinburgh); Shivun Khosla (Royal Surrey County Hospital); James Olivier (Southmead Hospital); Sum Yu Pansy Yue (St Thomas' Hospital); Gayathri Suresh (The Christie Hospital); Jack Wellington (University Hospital of Wales); Emmanuel Lorejo (West Suffolk Hospital); Mafdi Mossaad (Wexham Park Hospital); Yegor Tryliskyy (Worthing Hospital).

United States: Madison Crutcher (Hahnemann University Hospital); Marjan Alimi (NYU Langone Medical Center); Ioana Baiu (Stanford Health Care); Hossam Abdou (University of Maryland Hospital); Alison Conway (VA Palo Alto Hospital); Connor Peck (Yale New Haven Hospital).

Uruguay: Gabriela Wagner (Hospital De Clinicas); Mauro Andres Perdomo Perez (Hospital Espanol); Ivan Trostchansky (Hospital Maciel).

Zambia: Stanley Zulu (Kitwe Teaching Hospital); Mildred Nakazwe (University Teaching Hospital).
